# miRNA375-3p/rapamycin mediates the mTOR pathway by decreasing PS1, enhances microglial cell activity to regulate autophagy in Alzheimer's disease

**DOI:** 10.1016/j.heliyon.2024.e37589

**Published:** 2024-09-19

**Authors:** Yuxiang Wang, Zixuan Xiao, Hanlan Yin, Zhichao Ren, Xueting Ma, Yibo Wang, Yan Zhang, Xueqi Fu, Fuqiang Zhang, Linlin Zeng

**Affiliations:** a Key Laboratory for Molecular Enzymology and Engineering of Ministry of Education, School of Life Science, Jilin University, Changchun, 130012, China; b Scientific Research Centre of China-Japan Union Hospital, Jilin University, Changchun, 130033, China

**Keywords:** Alzheimer's disease, microRNA-375-3p, Autophagy, Microglia activation

## Abstract

The clinical prevention, diagnosis, treatment, and drug development of Alzheimer's disease (AD) require urgent detection of novel targets and methods. Autophagy and microglia are significantly associated with the pathogenesis of early AD. This study indicated that microRNA-375-3p can inhibit autophagy by promoting mTOR phosphorylation in normal physiological conditions, while microRNA-375-3p promoted autophagy and enhanced neural repair by inhibiting the expression of presenilin 1 in early AD pathogenesis. Furthermore, co-treatment of rapamycin, and microRNA-375-3p can synergistically promote the autophagy and microglial activation in a neuroprotective manner, clear Aβ accumulation, repair nerve damage, and alleviate cognitive dysfunction and memory defects in APP/PS1 TG mice. This research revealed the impact and mechanism of miR375-3p on the early stage of AD through *in vivo* and *in vitro* experiments and provides new ideas and directions for the early treatment of AD.

## 1 Introduction

Alzheimer's Disease is a neurodegenerative disorder that most commonly affects the elderly population. Currently, the specific mechanisms underlying neural damage and cognitive impairment in AD remain unclear [1].Furthermore, there are many challenges in Clinical drug development, such as poor target specificity, strong side effects, and poor absorption through the blood-brain barrier [[2], [3], [4], [5], [6]]. Therefore, the identification of new targets and the development of innovative therapies is urgently required.

Presenilin 1 (PS1) is primarily located in the endoplasmic reticulum and Golgi apparatus, and as a transmembrane protein, its well-known role in AD is as a component of the γ-secretase complex that mediates the cleavage of Amyloid Precursor Protein (APP) [7]. In the γ-secretase complex, PS1 is the gene with the most mutations discovered so far, and current research has found that the mutations in PS1 that cause severe AD are often completely dominant, despite PS1 mutations being one of the main causes of AD. However, the absence of PS1 would also have serious consequences, as it would result in the absence of the Notch signaling pathway [8], thus effective regulation of PS1 in AD may be a suitable therapeutic approach.

The literature has indicated that the normal occurrence of autophagy is crucial for the clearance of brain Amyloid β (Aβ) [8,9], and microglia activation participates in the clearance of extracellular Aβ oligomerss [10]. Futhermore, mTORC1 complex has been indicated to inhibit the formation of downstream ULK1 complexes, preventing autophagy in AD mice [11]. When autophagy is downregulated, intracellular and extracellular Aβ loads increase, exacerbating AD pathology [12,13]. Choi et al. revealed that in AD models, autophagy can stimulate glial cells, thereby accelerating Aβ clearance [14]. Amin et al. found that inducing autophagy prevents brain glial cell aging in AD mice, restoring cognitive abilities [15].

Microglia are the primary line of defense in the immune response of the nervous system, and significantly protect the central nervous system against external invasion [16]. Activation of microglia typically results in two phenotypes: M1 and M2, where M1 polarization causes an inflammatory response and M2 polarization promotes neural repair [17,18]. In AD, there are alterations in the proportion of M1 and M2 microglia as the disease progresses. Studies have demonstrated that early-stage AD patients exhibit a predominant accumulation of M2 microglia around Aβ plaques in their brains. In contrast, Chakrabarty et al. used an M2 overexpression AD mouse model and revealed that overexpression of M2-type microglia alone did not relieve AD but instead exacerbated Aβ deposition [19]. Therefore, judicious activation of microglia may represent a promising therapeutic approach for treating AD.

microRNAs (miRNAs) are 18–25 nucleotides long, widely present in organisms, and are essentially associated with neurodegenerative diseases [20,21]. miRNAs function by binding to their target mRNAs, either inhibiting mRNA translation or promoting mRNA degradation [22]. Furthermore, miRNAs can potentially treat AD as it has the advantage of penetrating through the blood-brain barrier and acting through endogenous pathways. Although miRNAs have limited inhibition of a single target, they can regulate multiple molecular pathways simultaneously [23]. Moreover, they are highly stable within the body, and can resist various adverse conditions [24]. Zhai et al. that exosomes containing miRNA-22 improved memory and motor skills in AD mice by suppressing apoptosis and reducing the release of inflammatory factors [25]. Mai et al. intranasally administered miR-146a agomir through intranasal delivery in AD mice, rescuing cognitive impairments through the inhibition of glial cell activation, suppression of neuroinflammation, and promotion of Aβ clearance [26].

This study screened miR375-3p, which has been indicated to be associated with AD, autophagy regulation, and glial cell activation. To investigate the potential value of miR375-3p in AD, its *in vitro* and *in vivo* therapeutic effects and mechanisms were assessed. It was revealed that in APP/PS1 Tg mice, rapamycin combined with miR375-3p-agomir significantly improved cognitive impairment, memory deficits, and reduced Aβ deposition in the model mice. Overall, this study indicated the potential role of miR375-3p in AD, providing evidence for its therapeutic use for AD treatment.

## 2 Material and methods

### 2.1 Screening process of miRNA related to AD, autophagy and microglia activation

We utilized a total of 63 gene sets for miRNA screening, including 20 pathways related to AD, 17 pathways related to autophagy, and multiple pathways related to microglial cell activation, comprising 7 JNK-related pathways, 9 MAPK-related pathways, 9 NF-κB-related pathways, and the PI3K pathway. These pathways were sourced from the Gene Set Enrichment Analysis (GSEA) database (https://www.gsea-msigdb.org/gsea/index.jsp) (https://www.pnas.org/doi/10.1073/pnas.0506580102), (https://www.nature.com/articles/ng1180). For miRNA interaction prediction, we employed three different mRNA-miRNA interaction databases: ENCORI, miRTarBase, and RNA22. During the prediction process, we utilized R packages such as ggsci, dplyr, and data.table for assistance in data analysis. We conducted a quantitative analysis of the predicted miRNAs and sorted them based on their frequency of occurrence. The top 100 miRNAs by frequency were selected for subsequent analysis.To predict genes that interact with miR-375, we utilized the TargetScan database (https://www.targetscan.org/mamm_31/) [DOI.10.1126/science.aav1741]. The results of the prediction were ranked in descending order based on the Aggregate PCT score, and the top 580 genes from the list were selected for further validation. For functional enrichment analysis, we employed DAVID (https://david.ncifcrf.gov/home.jsp) [https://doi.org/10.1093/nar/gkac194, DOI.10.1038/nprot.2008.211], using official gene symbols as identifiers and selecting Homo sapiens as the species. We performed Gene Ontology (GO) and Kyoto Encyclopedia of Genes and Genomes (KEGG) pathway enrichment analysis. Genes were filtered based on a P-value threshold of ≤0.5, and the top 18 genes were sorted in ascending order for presentation.

### 2.2 Cell culture

SH-SY5Y cells were purchased from Neogene Biochemical Technology Co., LTD (Liaoning, China) (Cat No.CBR-130335). HMC3 cells were purchased from Warner Biotechnology Co., LTD (Wuhan, China)(Cat No.WN-10265). The cells were cultured in DMEM high-glucose medium containing 10 % fetal bovine serum, supplemented with 100 IU/mL of penicillin and 100 μg/mL of streptomycin. All cells were maintained at 37 °C in a humidified atmosphere containing 5 % CO_2_.

### 2.3 Extraction of whole protein from cells and brain tissue samples

Cell lysis buffer [containing 50 mM Tris-HCl (pH 6.8), 8 M urea, 0.1 mM DTT, 2 % (w/v) SDS, 1 % (v/v) protease inhibitor, 1 % (v/v) PMSF, 1 % (v/v) phosphatase inhibitor] was added when harvesting the samples, and the cells were scraped and incubated on ice.

For mouse brain tissue samples, the frontal and prefrontal lobes were collected after liquid nitrogen freeze-drying and then added to cell lysis buffer, followed by incubation on ice.

The total protein extract of cells and mice in each group was collected, and the concentration of total protein extract was determined by BCA protein concentration assay kit (Meilunbio, Cat No. MA0186).

### 2.4 Western Blot

The amount of each sample was adjusted according to the protein concentration to ensure that the total protein of each sample was 20 μg. The electrophoresis conditions were set: 90 V, 20 min in the first stage; The second stage is 120 V 70 min. After SDS-PAGE, proteins were transferred to a PVDF membrane (100 Ma, 2 h, 4 °C). The membrane was blocked with 5 % skim milk at room temperature with gentle agitation for 1 h and then incubated with the primary antibody overnight at 4 °C. After washing the membrane, it was incubated with an HRP-conjugated secondary antibody at room temperature with gentle agitation for 1 h. Finally, ECL detection was performed, and the results were analyzed using ImageJ software (Details of the antibodies are provided in Table 1.).

**Table 1:** List of antibody usage.

Name	dilution rate	Source	Cat. No
Anti-PS1	WB 1:1000	Abclone	A19103
Anti-Syn	WB 1:2000	Abcom	ab32127
Anti-ATG14	WB 1:1000	Proteintech	19491-1-AP
Anti-GAPDH	WB 1:5000	Bioss	bsm-33033M
Anti-Beclin1	WB 1:1000，IF 1:200，IHC 1:200	Abclone	A22361
Anti-LC3B	WB 1:2000，IF 1:200	Abclone	A19665
Anti-Mfn2	WB 1:1000	Abclone	A12771
Anti-Grp75	WB 1:5000	Bioworld	BS1153
Anti-APP	WB 1:1000	Servicebio	GB14006
Anti-APP-CTFβ	WB 1:1000	Biolegend	803001
Anti-PARP	WB 1:1000	Cell Signaling Technology	9542
Anti-Caspase-3	WB 1:1000	Cell Signaling Technology	9665
Anti-BDNF	WB 1:2000，IF 1:200	Novus	NBP2-67410
Anti-Aβ	IF 1:200，IHC 1:200	ThermoFisher	71–5800
Anti-β-actin	WB 1:1000	Santa Cruz	sc-47778
Anti-p-mTOR	WB:1:2000	Proteintech	67778-1-IG
Anti-p-p70S6K	WB:1:2000	Abclone	AP1389
Anti-p-ULK1	WB:1:1000	Abclone	Abclone
Anti-GFAP	WB:1:1000	Cell Signaling Technology	3670
Anti-IBA-1	WB:1:500，IF:1:200，IHC:1:1000	Affinity Biosciences	DF6442
Anti-CD80	WB:1:1000，IF:1:200，IHC:1:200	Proteintech	66406-1-IG
Anti-CD68	WB:1:1000，IF:1:200	Abclone	A15037
Anti-CD206	IHC 1:500	Abcam	ab300621
Goat Anti-Rabbit IgG H&L antibody	WB 1:2000	Bioss	bs-0295G
Goat Anti-Mouse IgG H&L antibody	WB 1:2000	Bioss	bs-0296G

### 2.5 Immunofluorescence staining

Immunofluorescence staining is a method for staining specific proteins in cells and tissues using the antibody-antigen reaction, which has the characteristics of high specificity and sensitivity. Cells were fixed with 4 % paraformaldehyde at room temperature, followed by permeabilization with 0.1 % Triton X-100. Fresh tissues were fixed with 4 % paraformaldehyde, embedded, and sectioned into paraffin slices. Paraffin sections were deparaffinized by immersing them in xylene at room temperature, followed by another immersion in fresh xylene. Xylene was then removed, and sections were rehydrated sequentially with anhydrous ethanol, 90 % ethanol, 80 % ethanol, and 70 % ethanol. For antigen retrieval, each section was treated with 50 μL–100 μL of proteinase K and incubated at 37 °C. Afterward, cells were incubated with 5 % goat serum at room temperature. Next, the primary antibody (diluted at 1:200) was applied and incubated overnight at 4 °C. Subsequently, cells were incubated with FITC- or PE-conjugated IgG antibodies (diluted at 1:300) at room temperature, protected from light. Finally, cells were mounted with anti-fading mounting medium and observed using laser confocal microscopy.

### 2.6 JC-1 staining

In this experiment, a mitochondrial membrane potential detection kit (JC-1) was used (Solarbio, Cat No. M8650). The JC-1 probe accumulates and emits red fluorescence in normal mitochondrial matrices. When the membrane potential decreases, JC-1 cannot be retained in the mitochondrial matrix, resulting in monomers that emit green fluorescence. The specific steps were as follows: Cell samples of each group were prepared in advance in the 24-well plate. JC-1 staining working solution was prepared according to the instructions, and cells were incubated at 37 °C for 30 min. Subsequently, observations were made using a confocal microscope.

### 2.7 ROS detection experiment and tunel experiment

Elevated levels of reactive oxygen species (ROS) and the production of intracellular broken DNA are both landmark events in the development of AD. Here we use the corresponding probe for detection, the specific methods are as follows: Cell samples of each group were prepared in advance in the 24-well plate. Cells were separately treated with DCFH-DA probe (10 μM in serum-free culture medium for ROS detection) (Meilunbio, Cat No. MA0219) and Tunel detection solution (Meilunbio, Cat No. MA0223). They were then incubated at 37 °C in the dark for 30 min (ROS) and 60 min (Tunel). Subsequently, fluorescence was detected with excitation at 488 nm.

### 2.8 Total RNA extraction and reverse transcription

Total RNA was extracted from both cells (3 × 10^6^ cells per group) and mouse blood (200 μL) by adding 1 mL of TransZol Up (Transgen bio, Cat No. ER501-01-V2). The obtained total RNA was used as a template for cDNA synthesis using a reverse transcription kit (Transgen bio, Cat No. AU311-02).

### 2.9 Real-time PCR

In this study, Real-time PCR experiments were performed using BlasTaq™ 2X qPCR MasterMix (Abmbio,Cat No. G891), and data collection and analysis were carried out using the Applied Biosystems 7500 Real-time PCR system. Each sample was analyzed in triplicate. The 2-ΔΔCT method was employed to normalize the Actin cDNA. The primers used in this study are listed in Table 2.

**Table 2:** List of qRT-PCR primers.

Gene primer	Sequence (5′-3′)
mActin-s	TTCCAGCCTTCCTTCTTGGGTAT
mActin-as	GTTGGCATAGAGGTCTTTACGG
mAPP-s	CTACGGAAACGACGCTCTCA
mAPP-as	CAGAACCTGGTCGAGTGGTC
mBDNF-s	ACTGCAGTGGACATGTCTGG
mBDNF-as	CTGCAGCCTTCCTTGGTGTA
mSYP-s	CATCTTCGCCTTTGCTACGT
mSYP-as	GGTACTCAAATTCGACTTCGAT
mPS1-s	GTCATCCACGCCTGGCTTAT
mPS1-as	TAACGTAGTCCACGGCGACA
mPink-1-s	TTGACTACAGCAAAGCCGATACCT
mPink-1-as	CTCACCAGCCGTCTTGCCTC
mMfn2-s	GCGCCAGTTTGTGGAATACG
mMfn2-as	TCGGGTGATGTCAACTTGCT
mBeclin-1-s	CAATAATTTCAGACTGGGTCG
mBeclin-1-as	CCAGAACAGTATAACGGCAAC
mLC3B-s	CAAAGAGTGGAAGATGTCCG
mLC3B-as	GCTCATGTTCACGTGGTCAG
mCD68-s	GGGGCTCTTGGGAACTACAC
mCD68-as	GTACCGTCACAACCTCCCTG
mIba1-s	GAGCCAAAGCAGGGATTTGC
mIba1-as	GCTTCAAGTTTGGACGGCAG
mIL-6-s	GGGACTGATGCTGGTGACAA
mIL-6-as	TCTGCAAGTGCATCATCGTT
mCD80-s	TGGCCCGAGTATAAGAACCG
mCD80-as	TGTCTGCAGATGGGTTTCCA
miR-375-3p-s	AGCCGTCAAGAGCAATAACGAA
miR-375-3p-as	GTGCAGGGTCCGAGGT

### 2.10 AD model mice grouping and administration

The AD model mice used in this study were SPF-grade APP/PS1 transgenic mice, all of which were purchased from Shanghai Model Organisms Center, Inc. Wild-type mice used in this study were also SPF-grade and purchased from Beijing SPF Biotechnology Co., Ltd. All experimental animals were housed and cared for in accordance with the requirements of the Welfare Ethics Committee, College of Life Sciences, Jilin University. Ethics review acceptance number: YNPZSY2023005. IACUC Issue number: (2023) YNPZSY (0201). The experiments were conducted in compliance with the regulations of the People's Republic of China on the management of laboratory animals. Adequate measures were taken during the experiments to minimize pain and discomfort in the mice. All mouse strains used in this study were C57BL/6J (Male), and all were 6 months old. There were 20 mice in each group for both the APP/PS1 transgenic and wild-type mice. The APP/PS1 transgenic mice were divided into 4 groups, each consisting of 5 mice, while the wild-type mice were also divided into 4 groups, and the drug administration schedule was consistent with that of the APP/PS1 transgenic mice (see Table 3 for grouping details). The miR-375-3p-agomir and NC-agomir solutions used in this experiment were prepared with sterile DEPC water, and each injection was 2 nmol/kg, administered *via* tail vein injection. Rapamycin was used at a concentration of 2 mg/kg, and it was administered *via* intraperitoneal injection using a solution in 0.8 % physiological saline [[27], [28], [29], [30]].

**Table 3:** Grouping of animal experiments and method of administration.

Group	Injection cycle
WT Con group	5 numbers of wild type mice were injected with NC-agomir once every other day for 5 consecutive times. 0.8 % saline was injected once every other day for 7 consecutive injections.
WT + rapamycin group (n = 5)	wild type mice were injected with NC-agomir once every other day for 5 consecutive times. The rapamycin solution was injected every other day for 7 consecutive injections.
WT + miR375-3p group (n = 5)	wild type mice were injected with miR375-3p-mimic-agomir once every other day for 5 consecutive times. 0.8 % saline was injected once every other day for 7 consecutive injections.
WT + rapamycin + miR375-3p group (n = 5)	wild type mice were injected with miR375-3p-mimic-agomir once every other day for 5 consecutive times. The rapamycin solution was injected every other day for 7 consecutive injections.
AD Con group (n = 5)	APP/PS1 Tg mice were injected with NC-agomir once every other day for 5 consecutive times. 0.8 % saline was injected once every other day for 7 consecutive injections.
AD + rapamycin group (n = 5)	APP/PS1 Tg mice were injected with NC-agomir once every other day for 5 consecutive times. The rapamycin solution was injected every other day for 7 consecutive injections.
AD + miR375-3p group (n = 5)	APP/PS1 Tg mice were injected with miR-375-3p-mimic-agomir once every other day for 5 consecutive times. 0.8 % saline was injected once every other day for 7 consecutive injections.
AD + rapamycin + miR375-3p group (n = 5)	APP/PS1 Tg mice were injected with miR375-3p-mimic-agomir once every other day for 5 consecutive times. The rapamycin solution was injected every other day for 7 consecutive injections.

### 2.11 Behavioral testing

Morris Water Maze: The Morris water maze test was conducted after completing the injection cycle. Edible white paint was added to the water pool, which was divided into 4 quadrants: A1, A2, A3, and A4. The hidden platform was placed in quadrant A3. The water level in the pool was maintained 1–2 cm above the hidden platform, and a heating device was used to maintain the water temperature at 28–30 °C. The experiment spanned 10 days, with the first 3 days being the training phase and the subsequent 7 days being the experimental phase. Four trials were conducted each day, with mice released from quadrants A1, A2, A4, and the center of the pool, respectively. Mice were given 60 s to find the hidden platform. If they did not locate it within the allotted time, they were guided to it manually. After 10 s of adaptation, the trial was terminated, and the escape latency and path of the mice in finding the hidden platform were recorded [31].

Y-Maze Spontaneous Alternation Test: Prior to each trial, mice were first allowed to adapt to the Y-maze system for 5 min. After 1 h, the mice were placed in the center of the Y-maze, and their exploration of the 3 arms was recorded over a 10-min period. The proportion of times the mouse consecutively entered three different arms was calculated. The experimental environment was thoroughly cleaned after each trial.

Y-Maze Novel Arm Exploration Test: This test was conducted after the spontaneous alternation test. A partition completely blocked access to the novel arm, leaving only the starting arm and the other arms open. Mice were placed in the system and allowed to freely explore for 5 min. After 1 h, the partition blocking the novel arm was removed, and the mice were placed in the starting arm. The time spent entering the novel arm and the exploration distance within 3 min were recorded. The experimental environment was cleaned thoroughly after each trial [32].

### 2.12 *In vivo* fluorescence imaging

Fluorescence Experiment: One day prior to the fluorescence experiment, miR375-3p-mimic-agomir-cy3 (2 nmol/kg) and 0.8 % saline were injected separately. Before the experiment, APP/PS1 Tg mice were anesthetized using tribromoethanol (Meilunbio, Cat No. MA0478). Their back and head fur were removed using a depilatory device. The mice were then placed in a small animal live imaging system (FOBI) to observe the distribution of miR375-3p-mimic-agomir-cy3.

### 2.13 Mice sample treatment

After the live experiment was completed, the mice in each group were killed by carbon dioxide euthanasia device, and then blood was immediately let out (blood from eyeballs) and blood was collected by anticoagulant collection tube. After the experiment, the mice were dissected, and the five organs of the heart, liver, spleen, lung and kidney were fixed in 4 % paraformaldehyde for HE staining, and the brain tissues were taken for staining and western blot experiments.

### 2.14 Tissue embedding and slicing

Fresh tissues were fixed in 4 % paraformaldehyde for 24 h. Afterward, they were subjected to a gradient dehydration process. The paraffin-embedded tissues were then processed using an embedding machine. Molten paraffin was poured into an embedding frame, and the tissues were removed from the dehydration container and placed into the embedding frame before the paraffin solidified. The tissues were cooled at −20 °C, and once the paraffin solidified, it was removed and trimmed. Subsequently, paraffin sections were prepared using a microtome with a thickness of 4 μm.

### 2.15 Immunohistochemical staining

Immunohistochemistry is a technique that uses the antibody-antigen binding reaction to detect specific antigens within tissues and cells. Its advantage is that it can quantify and localize the target, as follows: Tissue sections were deparaffinized following the same procedure as described for tissue section fluorescent staining. Antigen retrieval was performed using antigen retrieval buffer (pH 6.0). The sections were washed with PBS. Subsequently, they were incubated with 3 % BSA at room temperature. After another PBS wash, the sections were incubated with the primary antibody at 4 °C overnight. Following primary antibody incubation, the sections were washed with PBS. Next, secondary antibodies corresponding to the species of the primary antibodies were applied to cover the tissue sections, and they were incubated at room temperature in the dark. The sections were washed with PBS. DAB chromogen was used for staining, followed by a single distilled water wash. The sections were counterstained with hematoxylin, subjected to 1 % hydrochloric acid alcohol differentiation, rinsed with distilled water, and blue counterstained with ammonia water. After a final wash with distilled water, the sections were observed under a microscope, and images were captured using CaseViewer software.

The following antibodies were used for immunohistochemical analysis: anti-Aβ (ThermoFisher, Cat No. 71–5800), anti-CD80 (Proteintech, Cat No.66406-1-IG), anti-Beclin-1 (Abclone, Cat No. A22361), anti-Iba1 (Affinity, Cat No. DF6442).

### 2.16 HE staining

HE staining is one of the most intuitive ways to observe tissue pathology. The specific method is as follows: Tissue sections were deparaffinized using the same procedure as described for tissue section fluorescent staining. The sections were then stained with Harris hematoxylin (Baiqiandu bio, Cat No. B1000), followed by rinsing in distilled water. Differentiation was achieved with 1 % hydrochloric acid alcohol for a few seconds, followed by rinsing in distilled water. Counterstaining was performed with 0.6 % ammonia water, followed by rinsing in distilled water. After dehydration and clearing, the sections were air-dried at room temperature, mounted with neutral resin, observed under a microscope, and images were captured using CaseViewer software.

### 2.17 Nissl body staining

The number of Nissl bodies is one of the indicators of neuronal damage in the brain. Tissue sections were deparaffinized following the same protocol as mentioned earlier for tissue section fluorescent staining. The sections were then stained with Niissl stain (Baiqiandu bio, Cat No. B1007), followed by rinsing in distilled water twice. Differentiation was carried out with 1 % acetic acid, followed by rinsing in distilled water to stop the reaction. After air-drying, the sections were cleared in xylene, mounted with neutral resin, and observed under a microscope. Images were captured using CaseViewer software.

### 2.18 Elisa

The ELISA analysis method utilized in this study is the double antibody sandwich technique, and the specific protocol is as follows. The samples in this study are all mouse brain tissues, and the specific methods are as follows:Weigh approximately 50 mg of brain tissue from each group of mice, which has been pre-frozen in liquid nitrogen. Rinse the tissue with pre-cooled PBS buffer. Then, Add PBS (including 1 % Cocktail and 1 % PMSF). The tissue was broken by ultrasonic crushing instrument, and the homogenate protein concentration was determined using BCA kit. Set up sample wells, standard wells, and blank wells according Elisa kit requirements (Lunchangshuo bio, Cat No. ED-20037, ED-20161, ED-28376), diluting sample solutions based on measured concentrations. The standard curve is calculated using data from standard product holesand blank holes,and then usedto calculatethe target protein contentofeachsample.

### 2.19 Statistical analysis

All animals in this study underwent behavioral tests. For subsequent Real-time PCR, Western Blot, and various staining experiments, we utilized three or more independent samples for analysis. Given that our study involved grouping into sets of five mice, we ensured the independence and randomness of the experiments. All mouse and cell studies were analyzed by two-tailed unpaired Student's *t*-test or one-way ANOVA test when more than two groups were compared. Statistical analyses in studies were performed using GraphPad Prism 8 software (Pearson's correlation test). All data points were used in statistical analyses. Data represent the mean ± SEM, with a statistically significant difference defined as a value of P < 0.05.

## 3 Results

### 3.1 miRNA-375-3p plays a crucial role in both Alzheimer's disease (AD) and autophagy

To further investigate the pathogenic mechanisms of AD and new drug targets 63 gene sets were downloaded from the GSEA database. Furthermore, various prediction websites were employed for cross-prediction. A total of 49 miRNAs present at the intersection of all gene sets were identified (Fig. 1A). Denk et al. analyzed differential miRNAs in the cerebrospinal fluid of AD patients [33] and based on their dataset, the predicted miRNAs were compiled and revealed that only miR375-3p was present in the intersection of the two sets (Fig. 1B). These data suggest that miR375-3p is related to AD and autophagy, and is significantly downregulated in AD patient samples.

**Fig. 1A:**
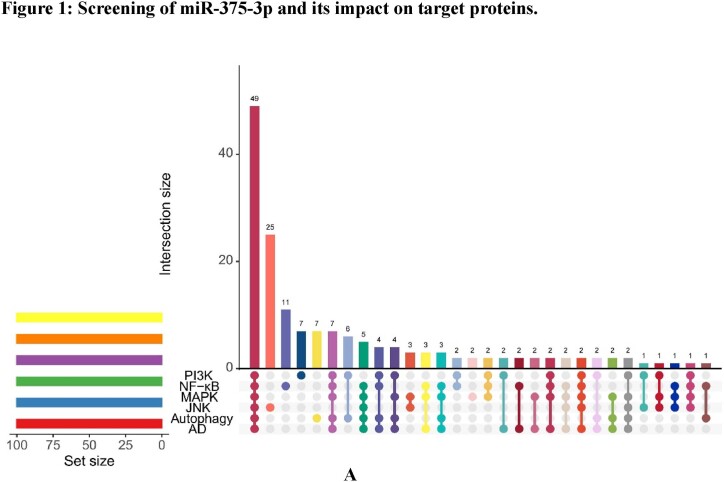
The mRNA-miRNA interactions were predicted using 63 gene sets related to Alzheimer's disease (AD), autophagy, and microglial cell activation.

**Fig. 1B:**
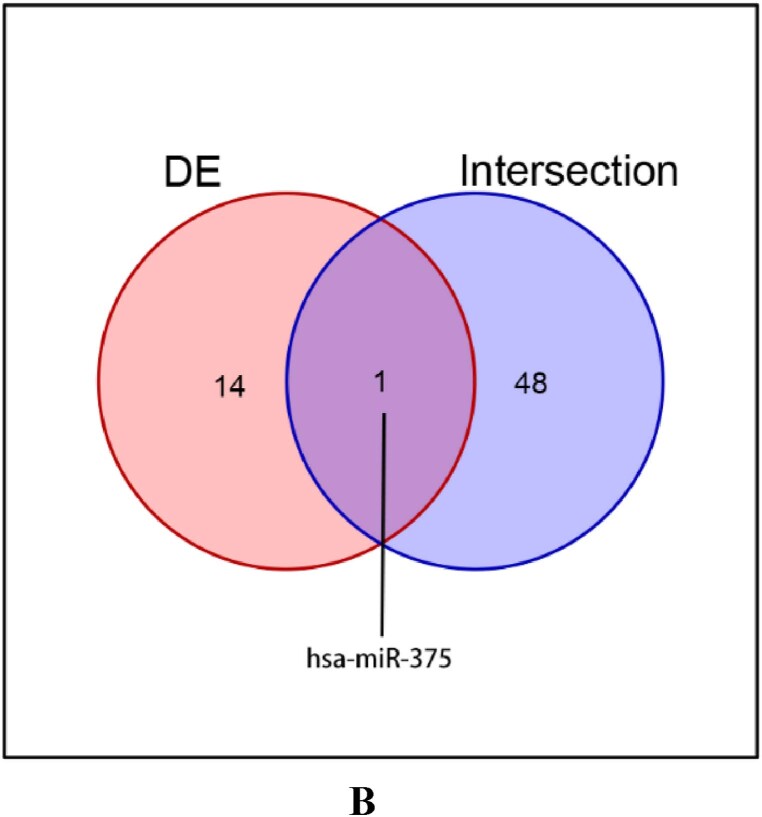
Venn diagram illustrating the independent and overlapping miRNAs between the predicted proteins in (A) and data sets published by Denk J and others.

The predicted target proteins of miR375-3p were predicted and enriched. Kyoto Encyclopedia of Genes and Genomes (KEGG) analysis indicated (Fig. 1C) that the target proteins of miR375-3p were mainly enriched in pathways related to AD as well as MAPK, mTOR, PI3K-AKT, and TGF-β signaling. Gene Ontology (GO) biological process analysis (Fig. 1D) revealed that miR375-3p target proteins were mainly involved in transcriptional regulation processes, cell proliferation, intracellular signal transduction, nervous system development, and protein phosphorylation, *etc*. Therefore, it was concluded that miR375-3p is associated with pathways related to AD, autophagy, and microglial cell activation. Furthermore, specific functions and molecular mechanisms of miR375-3p can be determined by further experiments.

**Fig. 1C and D:**
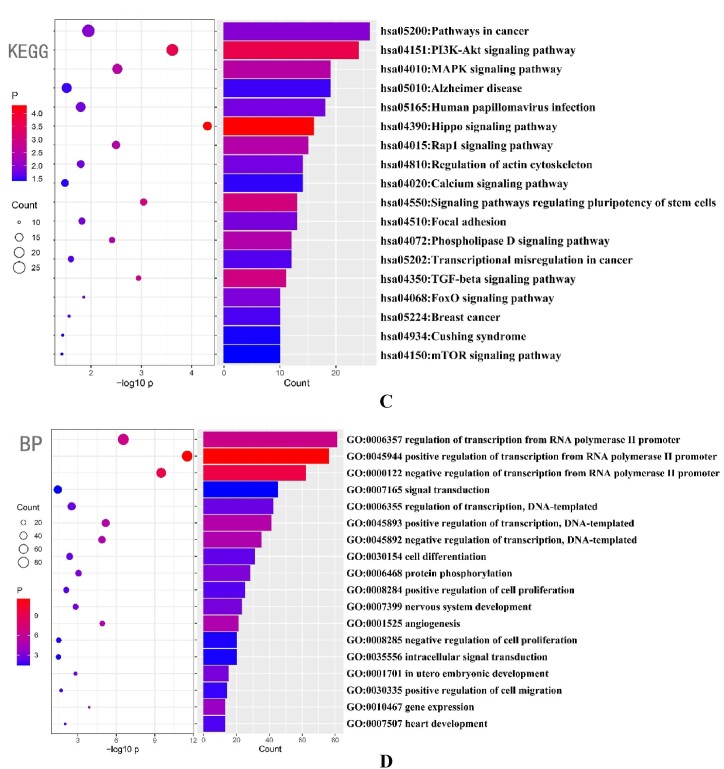
Bubble plots depicting the enrichment of miR-375-3p target proteins using KEGG (C) and GO biological processes. (D) The predicted results were sorted by the number of proteins in respective pathways.

Among the predicted target proteins of miR375-3p, several proteins closely related to AD and autophagy, including PS1, Synaptophysin (Syn), Brain-derived neurotrophic factor (BDNF), and ATG14 were identified (Fig. 1E). Among them, PS1, Syn and BDNF are classical markers of the pathogenesis of AD, and ATG14 plays a major role in regulating autophagy as a part of the PI3K complex in the autophagy pathway. It was revealed that after the transfection of SH-SY5Y cells with miR375-3p mimic, the expression levels of PS1, Syn, BDNF, and ATG14 proteins were significantly reduced compared to the NC group (Fig. 1F). Furthermore, after wild-type mice were treated with miR375-3p, hippocampus tissue samples indicated that the expression levels of PS1, Syn, BDNF, and ATG14 were all decreased compared with the control group (Fig. 1G). Moreover, the results from cell and tissue samples were consistent with the bioinformatics prediction results, indicating that miR375-3p mimic can regulate the expression of its target proteins *in vitro* and *in vivo*.

**Fig. 1E:**
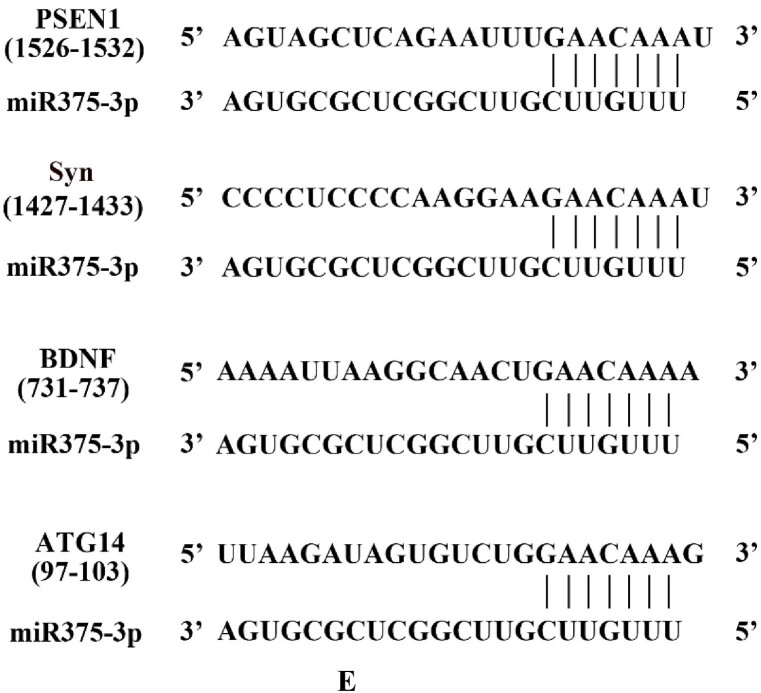
Prediction of miR-375-3p interaction sites with its target proteins.

**Fig. 1F_I:**
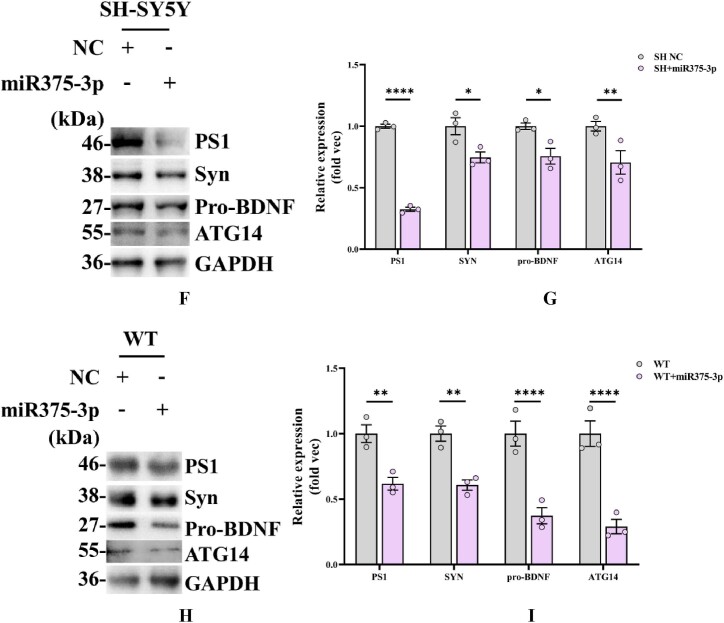
Western blot analysis of miR375-3p interacting proteins (results of three replicates). (F) Overexpression of NC or miR375-3p in SH-SY5Y cells and assessment of PS1, Syn, ATG14, and pro-BDNF protein expression levels. (G) Quantification of cell grayscale values using GAPDH as an internal reference *via* ImageJ. (H) The protein expression levels of PS1, Syn, ATG14, and pro-BDNF in the frontal and temporal lobes of wild-type mice injected with single-stranded NC-agomir or miR375-3p-mimic-agomir were assessed using ImageJ with GAPDH as an internal reference. (I) GAPDH was used as a normalized internal parameter (Fold vec) to analyze the gray values of each group. All data are presented as the mean ± SEM of at least three independent experiments and were compared using one-way ANOVA with Tukey's multiple comparisons test. *∗p < 0.05, ∗∗p < 0.01, ∗∗∗p < 0.001, ∗∗∗∗p < 0.0001*.

### 3.2 miR375-3p can inhibit Aβ_25-35_-induced apoptosis

Aβ_25-35_ is the neurotoxic peptide segment of amyloid beta protein, which is synthesized *in vitro* and used to construct cell models of AD. To determine when Aβ_25-35_ starts influencing SH-SY5Y cells, cells were treated for different durations (18, 24, and 48 h). Western blot results revealed that after exposure to Aβ_25-35_ for 18–48 h, the protein expression levels of PS1, Beclin1, and autophagy microtubule-associated protein 1 light chain 3β (LC3B) decreased over time, while the expression of glucose-regulated protein 75 (Grp75) began to decrease after 24 h. The expression of ATG14, LC3B, and mitofusin 2 (MFN2) was significantly reduced from 18 to 24 h but showed a reversal in trend thereafter. The experimental results demonstrate that Aβ_25-35_ induces cellular and mitochondrial autophagic damage. All six proteins detected in the first interval (0–24 h) exhibited a gradual decreasing trend. In the second interval (24–48 h), the protein expression levels of PS1, Beclin1, and Grp75 continued to decrease; ATG14 showed no significant change; and the expression levels of LC3B and MFN2 increased.We concluded that this may be related to the stress generated by SH-SY5Y cells themselves in response to long-term environmental changes, which involves more complex changes in macroautophagy and mitochondrial autophagy pathways. Therefore, according to the research purpose of this experiment, the induction time of Aβ_25-35_ in subsequent experiments was 24 h unless otherwise specified (Fig. 2A and B).

**Fig. 2A and B:**
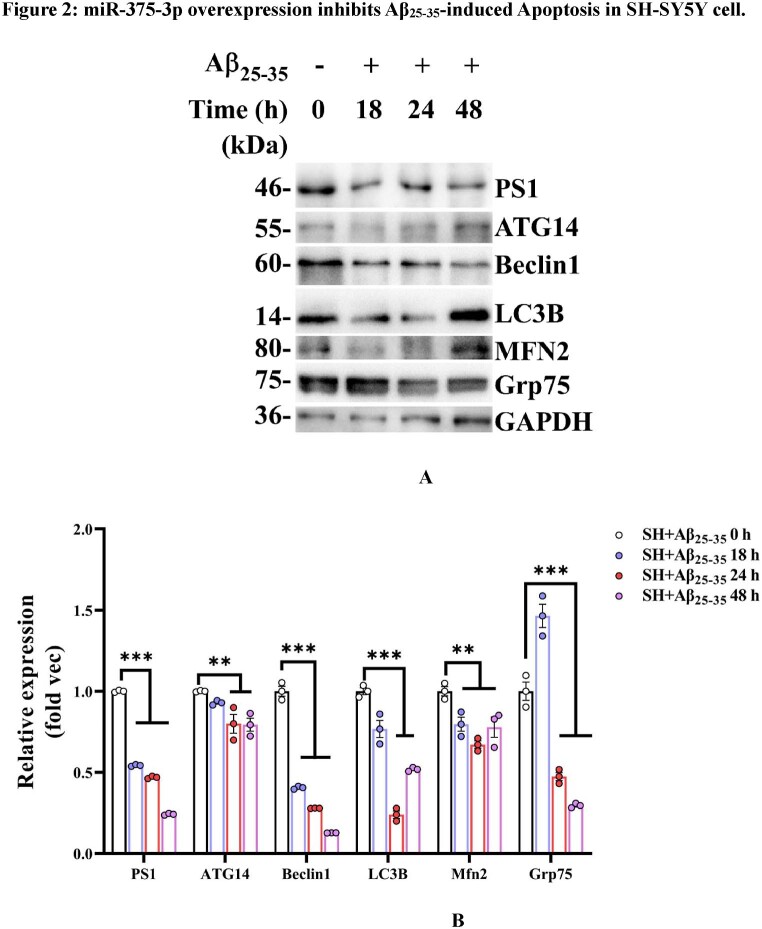
(A) Western blot analysis of PS1, ATG14, Beclin1, LC3B, MFN2, and Grp75 protein expression levels in SH-SY5Y cells exposed to various Aβ_25-35_ conditions at different time points. (B) The grayscale values for each group were quantified using ImageJ analysis. GAPDH was set as the internal reference.

PARP cleavage and up-regulation of caspase-3 expression indicate the occurrence of apoptosis, and the increase of APP-CTF fragment indicates the increase of the hydrolytic activity of APP. Western blot results indicated that miR375-3p inhibited PS1 expression, alleviated Aβ_25-35_-enhanced PS1 cleavage, and attenuated Aβ_25-35_-induced generation of APP-CTF and downregulation of Syn. Furthermore, miR375-3p overexpression mildly induced PARP cleavage and suppressed Beclin1 expression; however, completely blocked Aβ_25-35_-induced PARP cleavage and caspase-3 activation. Moreover, miR375-3p restoration reversed the Aβ_25-35_-induced reduced expression of the autophagic key protein Beclin1, while its effect on ATG14 was minimal (Fig. 2C and D). These data suggest that although miR375-3p overexpression mildly induces cell apoptosis, it significantly influences Aβ_25-35_-induced cell apoptosis, AD risk genes, and autophagic key proteins, indicating functional differences of miR375-3p in normal and AD conditions.

**Fig. 2C and D:**
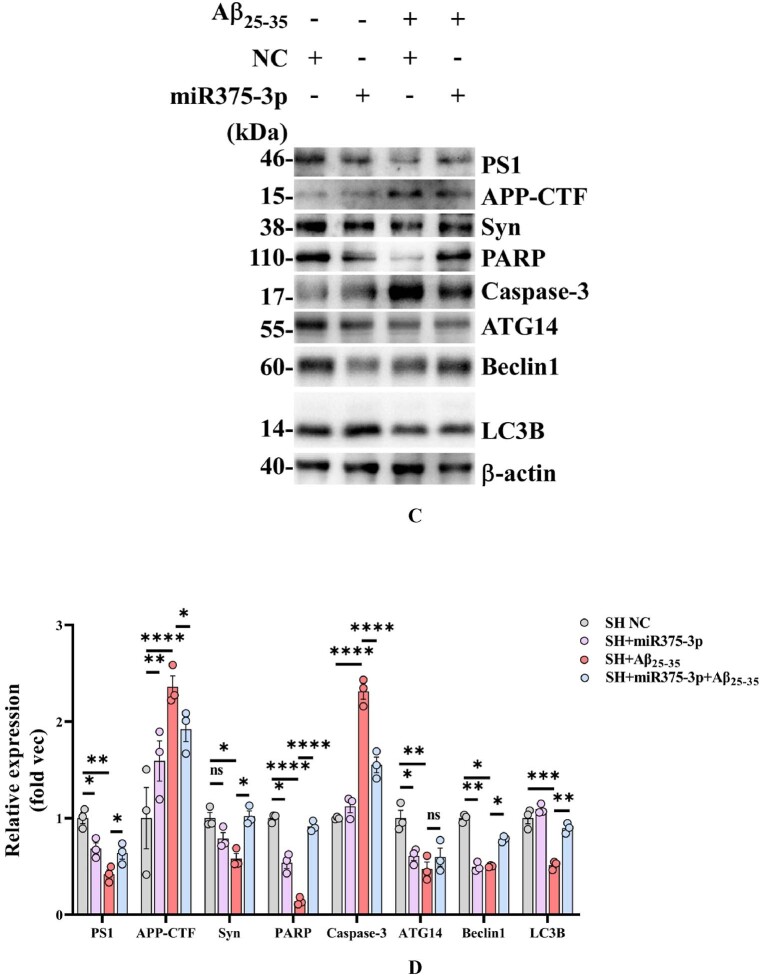
Western blot analysis of protein expression in Aβ_25-35_-induced SH-SY5Y cells after miR375-3p overexpression. (C) Western blot analysis was performed to detect the expression of AD and autophagy marker proteins in each group after SH-SY5Y cells were transfected with single-stranded NC or miR375-3p-mimic and treated with 20 μM Aβ_25-35_ for 24 h. (D) β-actin was used as an internal reference. For grayscale value quantification and analysis, ImageJ was employed.

The results of Tunel, reactive oxygen species (ROS), and JC-1 assays were consistent and indicated that miR375-3p-mimic attenuated Aβ_25-35_-induced DNA fragmentation in SH-SY5Y cells, reducing apoptosis from 28.35 ± 6.33 % to 9.6 ± 2.3 % (Fig. 2E and F). Intracellular ROS levels decreased from 23.97 ± 3.55 % to 5 ± 1.53 % (Fig. 2G and H) and the mitochondrial membrane potential showed significant recovery (Fig. 2I and J), indicating relief from ATP synthesis inhibition and mitochondrial damage. Moreover, the immunofluorescence staining results indicated that miR375-3p-mimic promoted phagocytic degradation pf Aβ in Aβ_25-35_-induced cells (Fig. 2K and L), alleviated the decreased expression of LC3B (Fig. 2K and N), but had no statistically significant effect on Beclin-1 (Fig. 2K and M).

**Fig. 2E and F:**
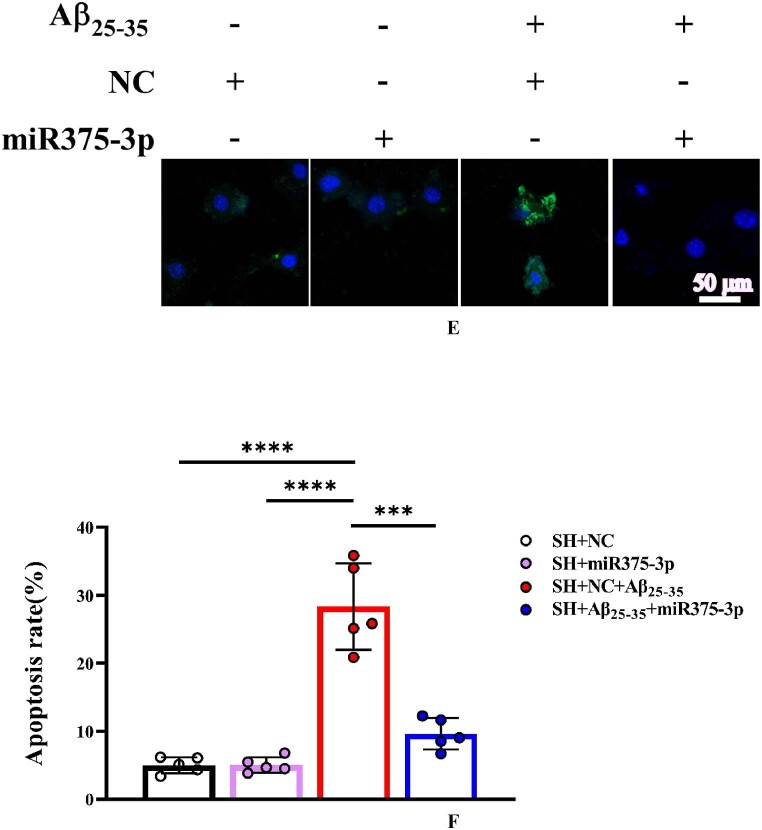
Laser confocal microscopy images of TUNEL staining in cells (400 × magnification) with a scale bar of 50 μm (E). Cell fluorescence intensity was quantified using Image-Pro Plus 6.0 to calculate the apoptosis rate (F).

**Fig. 2G and H:**
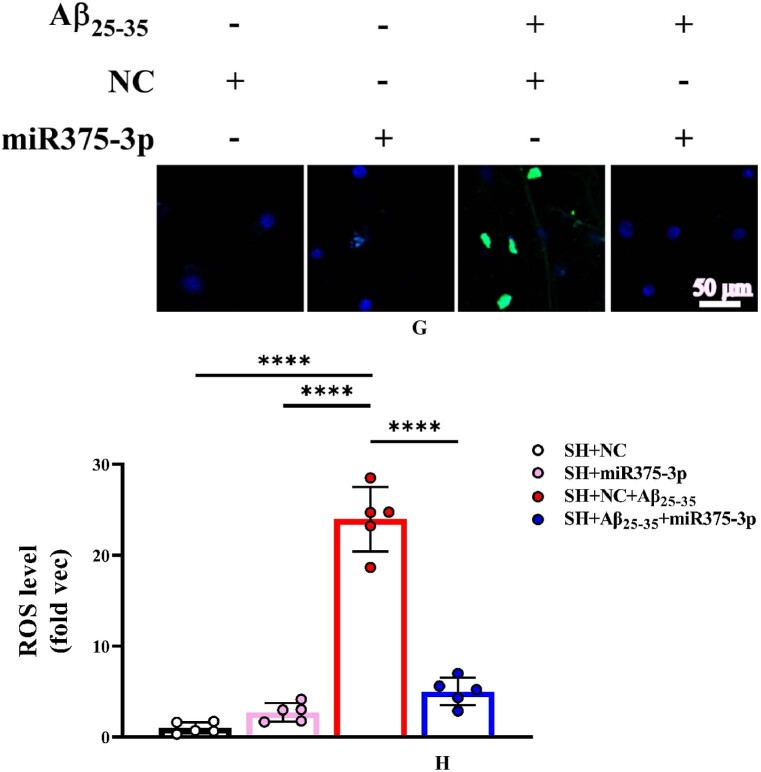
Cellular ROS immunofluorescence staining results (400 × magnification); Scale bar = 20 μm (G). Fluorescence intensity was quantified to determine ROS levels (H). ROS: reactive oxygen species.

**Fig. 2I and J:**
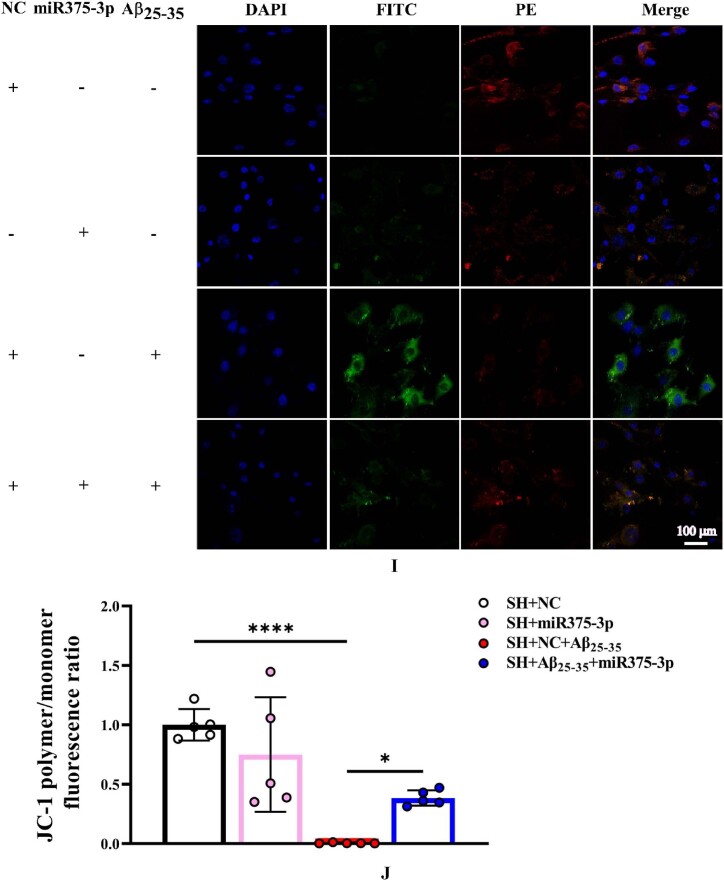
JC-1 probe staining of cellular monomers (Alexa Fluor 488 nm) and polymers (Alexa Fluor 633) (200 × magnification): scale bar of 100 μm (I). Fluorescence intensity was quantified to assess the JC-1 polymer/monomer ratio and the results are presented relative to the SH + NC group (J).

**Fig. 2K–N:**
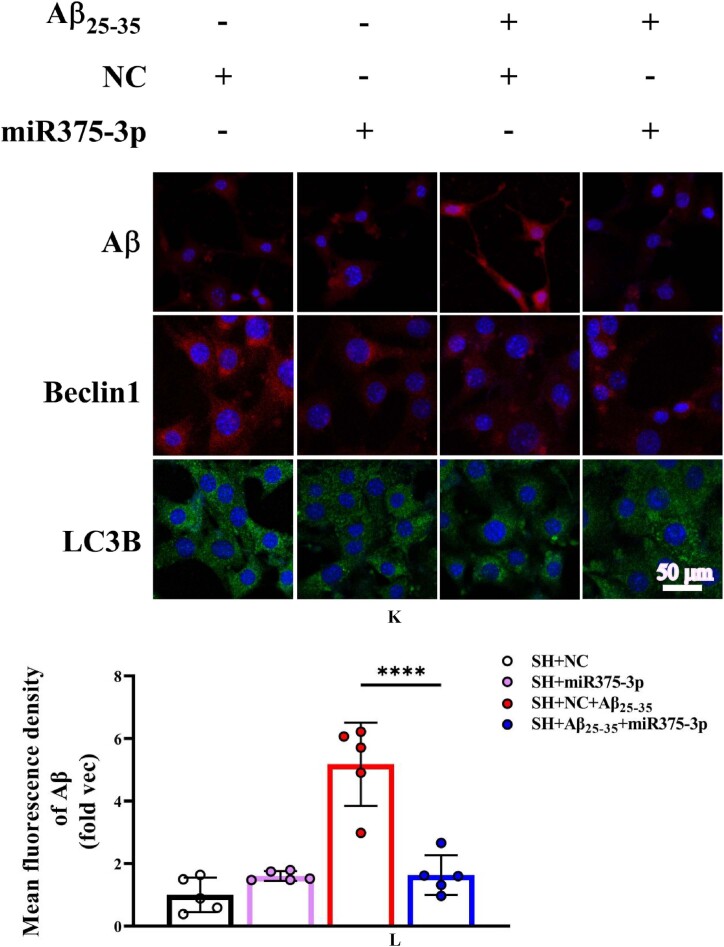

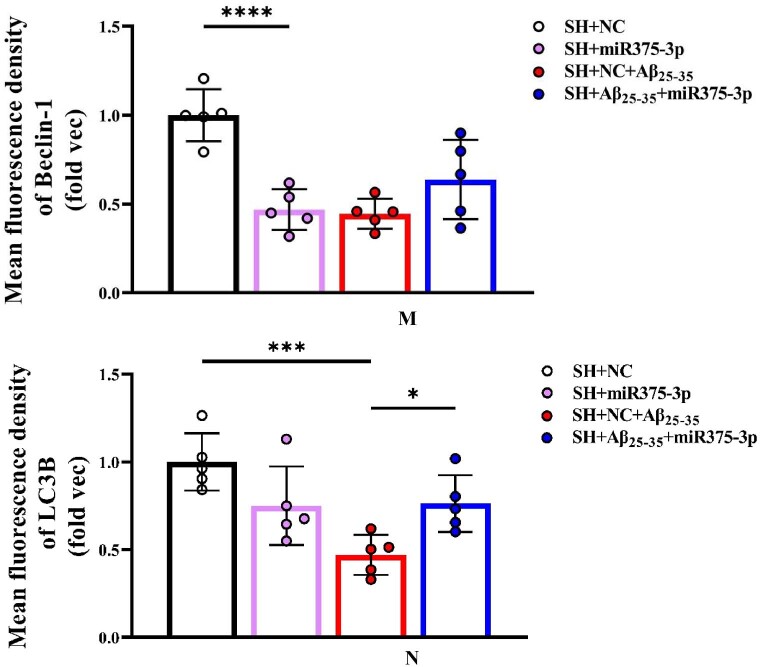
Fluorescence staining results for Aβ, Beclin1, and LC3B. (K) The fluorescence intensity of cells stained with anti-Aβ and anti-Beclin1 was detected at 633 nm, and that of anti-LC3B stained cells was detected at 488 nm; Scale bar = 50 μm. The fluorescence intensities of Aβ (L), Beclin1 (M), and LC3B (N) were quantified and compared across groups. All data are presented as the mean ± SEM of at least three independent experiments and were compared using one-way ANOVA with Tukey's multiple comparisons test. *∗p < 0.05, ∗∗p < 0.01, ∗∗∗p < 0.001, ∗∗∗∗p < 0.0001*.

### 3.3 The combination of rapamycin and miR375-3p-mimic alleviates Aβ_25-35_-induced apoptosis by promoting autophagy

To further elucidate the relationship and mechanism between autophagy and apoptosis in this study, we examined the effects of autophagy activator rapamycin and apoptosis inhibitor z-VAD-FMK. Z-VAD-FMK is a broad-spectrum inhibitor of caspases, which can suppress cell apoptosis caused by caspase activation. Rapamycin is an inhibitor of mTOR, which can promote autophagy by inhibiting the mTORC1 complex. As Fig. 3 A–C, Fig. 3D–FA–F indicates, Aβ25-35-induced decrease in Beclin1 and LC3B was caspase-dependent, which was evident by the significant increase in these proteins after the addition of the caspase inhibitor z-VAD-FMK, whereas the effects of the autophagy activator rapamycin on Beclin1 and LC3B were inconsistent. Therefore, in the subsequent studies, we mainly used rapamycin combined with miR375-3p to intervene in AD cells and mouse models to clarify the regulatory role of miR375-3p on autophagy.

**Fig. 3 A–C:**
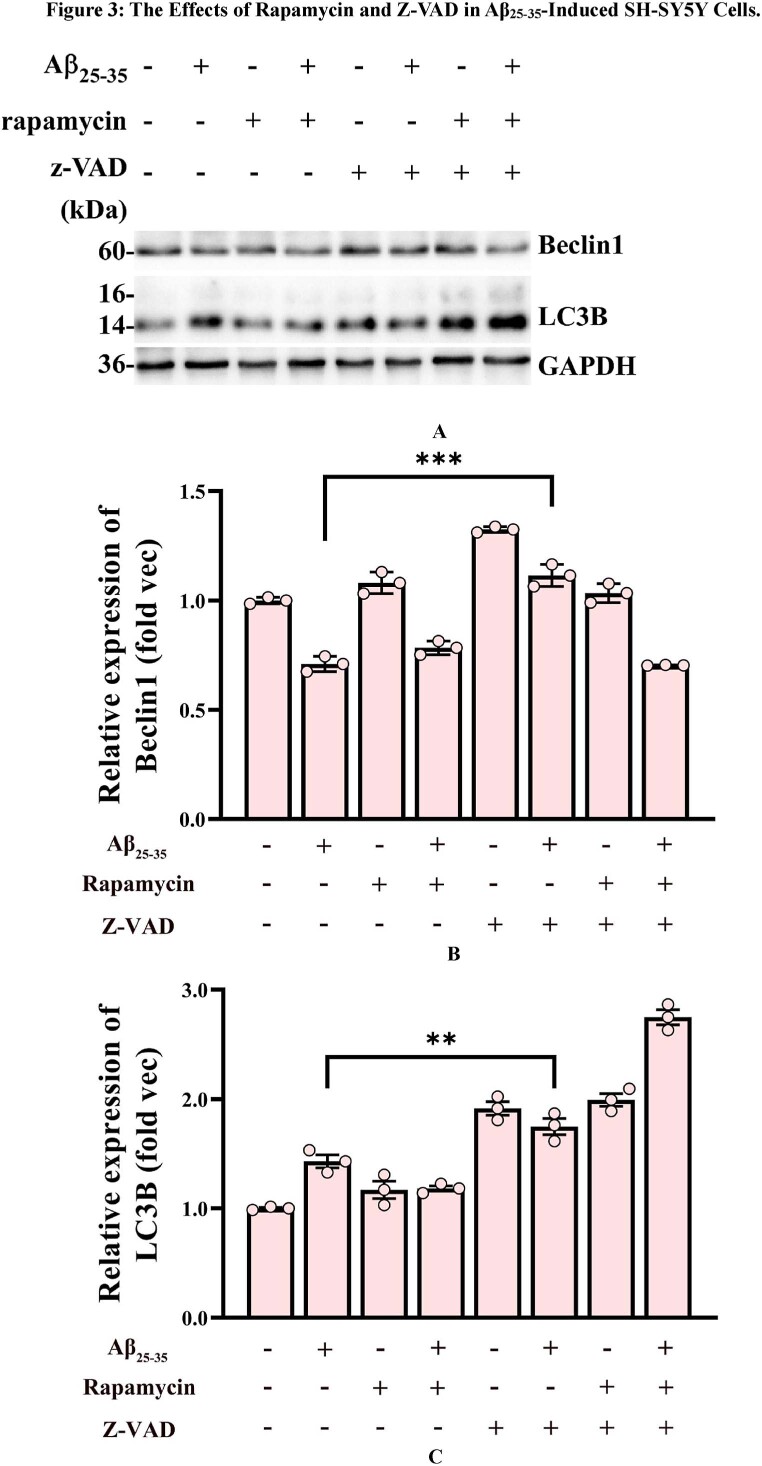
Western blot analysis of cellular Beclin1 and LC3B protein expression. (A) Western blot analysis of Beclin1 and LC3B levels was performed using adhered SH-SY5Y cells treated with Rapamycin and Z-VAD for 1 h, followed by Aβ_25-35_ induction for 24 h. (B and C) Grayscale values for Beclin1 (B) and LC3B (C) in each group were quantified using ImageJ GAPDH was used as the internal reference.

**Fig. 3D–F:**
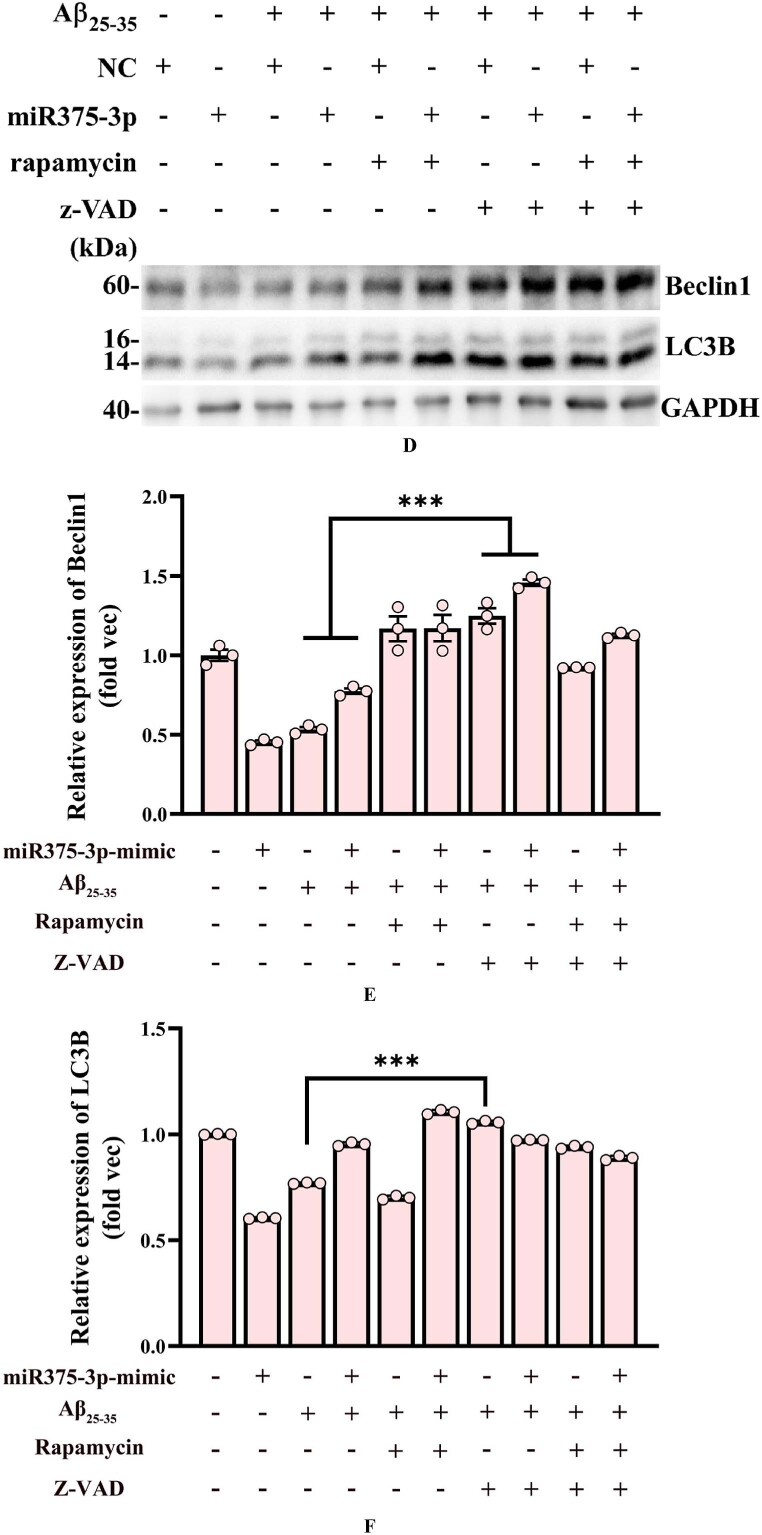
Western blot analysis of Beclin1 and LC3B protein expression in miR-375-3p overexpressing SH-SY5Y cells. (D) Beclin1 and LC3B expression in SH-SY5Y cells was assessed after cells were transfected with either single-strand NC or miR-375-3p in the presence of Rapamycin and Z-VAD for 1 h, followed by Aβ_25-35_ induction for 24 h. (E and F) Grayscale values for Beclin1 (E) and LC3B (F) in each group were quantified using ImageJ. GAPDH was used as the internal reference.

Tunel and ROS experiments further clarified the role of rapamycin. Tunel analysis indicated a significantly reduced amount of DNA fragmentation in the presence of rapamycin after Aβ_25-35_ induction. MiR375-3p-mimic in combination with rapamycin showed stronger inhibition of Aβ-induced apoptosis (Fig. 3G and H). These results were consistent with the detection of ROS levels (Fig. 3I and J).

**Fig. 3G and H:**
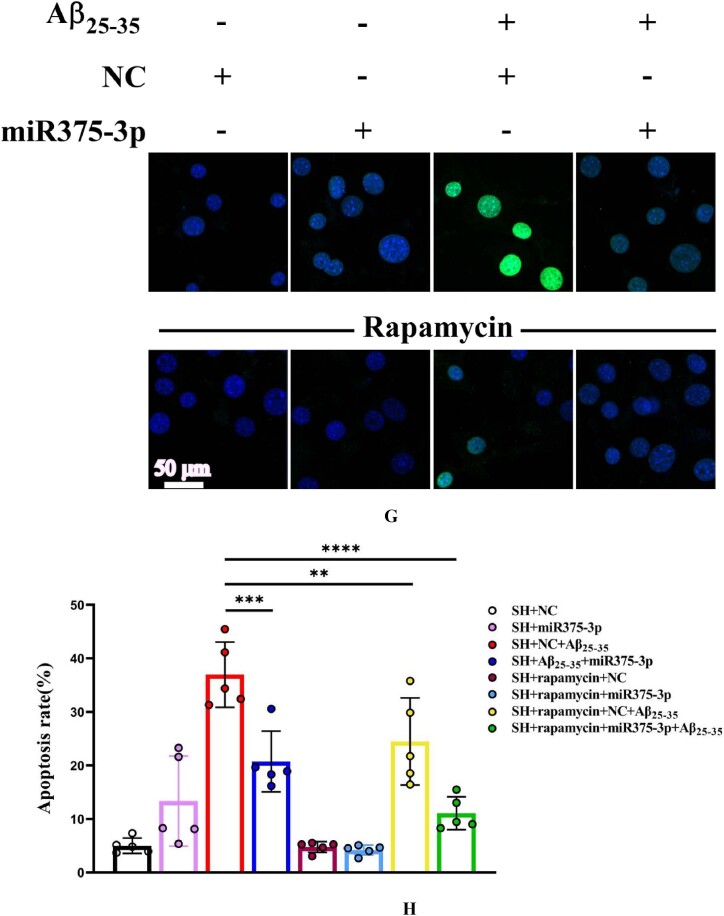
Tunel staining analysis of SH-SY5Y cell's apoptosis levels. Cells were first treated with Rapamycin for 1 h, followed by overexpression of strand NC or miR-375-3p for 24 h, and then the addition of Aβ_25-35_ for 24 h. (G) A laser confocal microscope with an Alexa Fluor 488 channel was used to assess the apoptosis rate of each group's cells, which were fixed and permeabilized before Tunel probe treatment for 1 h and then stained with DAPI for 5 min. Scale bar = 50 μm. (H) The fluorescence intensity in each group was quantified using Image-Pro Plus 6.0 to calculate the apoptosis rate.

**Fig. 3I and J:**
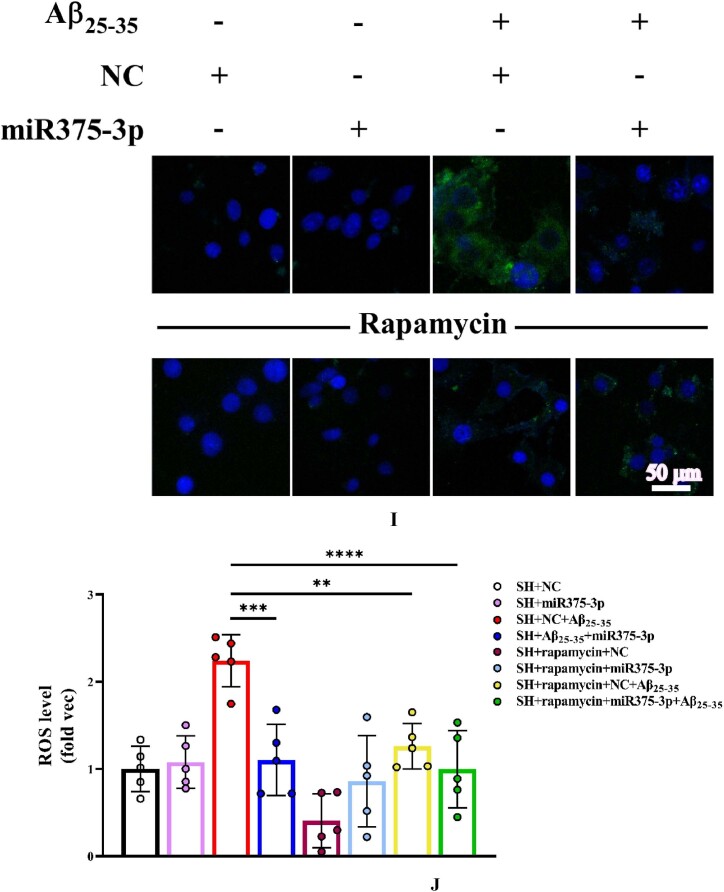
(I) ROS probe to detect intracellular ROS levels. Briefly, cells from each group were treated with the ROS probe for 30 min, washed, and then stained with DAPI for 5 min. The excitation light intensity was measured at 488 nm; Scale bar = 50 μm. (J) The fluorescence intensity in each group was quantified to determine the levels of reactive oxygen species. ROS: Reactive oxygen species.

Western blot results demonstrated that the addition of rapamycin increased the expression of MFN2 and Grp75 proteins, and had a slight restorative effect on Beclin1. Moreover, rapamycin enhanced LC3B expression. In addition, it was observed that MiR375-3p can protect against Aβ_25-35_-induced BDNF degradation. However, the addition of rapamycin did not further enhance the effects of miR375-3p, and its impact on PS1 was not significant (Fig. 3K and L).

**Fig. 3K and L:**
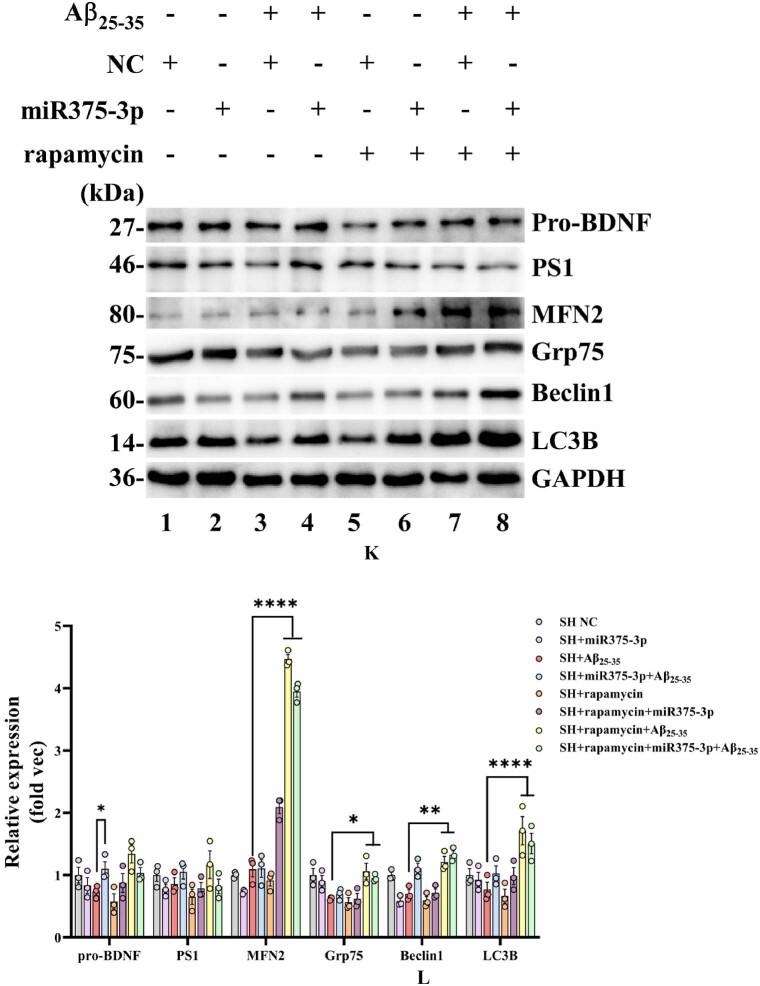
Western blot analysis of protein expression in Aβ_25-35_-induced SH-SY5Y cell after miR375-3p overexpression and rapamycin treatment. (K) Protein expression levels of LC3B, PS1, pro-BDNF, MFN2, Grp75, and Beclin1 in each group of cells. (L) Grayscale values of each group were quantified and analyzed using ImageJ. GAPDH was used as an internal reference.All data are presented as the mean ± SEM of at least three independent experiments and were compared using one-way ANOVA with Tukey's multiple comparisons test. *∗p < 0.05, ∗∗p < 0.01, ∗∗∗p < 0.001, ∗∗∗∗p < 0.0001*.

### 3.4 Assessment of the *in vivo* toxicity of rapamycin and miR375-3p-mimic-agomir

To evaluate the *in vivo* biosafety of rapamycin combined with miR375-3p-mimic-agomir, rapamycin, and miR375-3p-mimic-agomir were administered separately *via* intraperitoneal and tail vein injections, respectively, into wild-type and APP/PS1 Tg mice. After completing the injection cycle (Fig. 4A), tissues were stained with hematoxylin and eosin (H&E), which indicated no significant damage to the heart, liver, spleen, lungs, or kidneys of both wild-type and APP/PS1 Tg mice (Fig. 4B, Supplementary Fig. 1F). Mouse weight, phenotype, and survival status were normal, indicating that co-treatment of rapamycin and miR375-3p-mimic-agomir had relatively safe effects *in vivo.*

**Fig. 4A:**
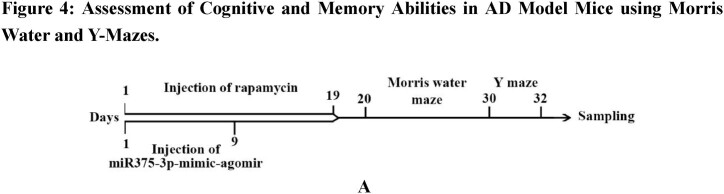
Design of the injection schedule and behavioral testing procedure. The APP/PS1 transgenic mice (28 weeks old) and wild-type (26 weeks old male) mice **were** used for this assay.

**Fig. 4B:**
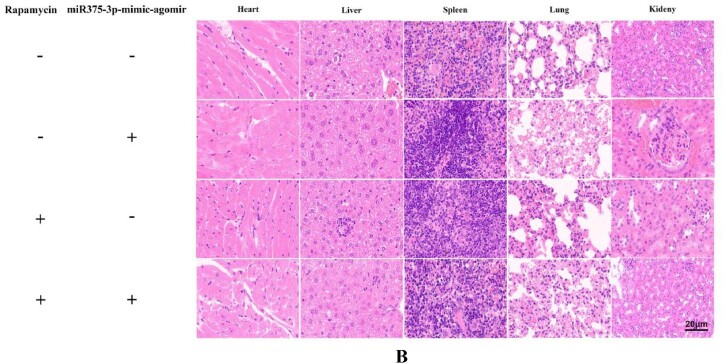
Rapamycin and miR-375-3p-mimic-agomir were administered through intraperitoneal and tail vein injections in APP/PS1 Tg mice. After treatment **completion**, organ samples were collected, and tissue sections were prepared for H&E staining analysis; Scale bar = 20 μm.

### 3.5 The combination of rapamycin and miR375-3p-mimic-agomir can improve cognitive and memory deficits in AD model mice

*In vivo,* imaging experiments identified the distribution of miR375-3p-mimic-agomir in wild-type and APP/PS1 Tg mice. It was revealed that after tail vein injection, miR375-3p-mimic-agomir-CY3 was primarily distributed in the limbs, brain, and ears, indicating its enrichment in the major lesion areas of APP/PS1 Tg mice (Fig. 4C, Supplementary Fig. 1E). After the treatment completion (Fig. 4A), the effects of mono- and co-treatment of rapamycin/miR375-3p-mimic-agomir on spatial memory and learning ability in AD model mice were assessed through Morris water and Y maze experiments.

**Fig. 4C:**
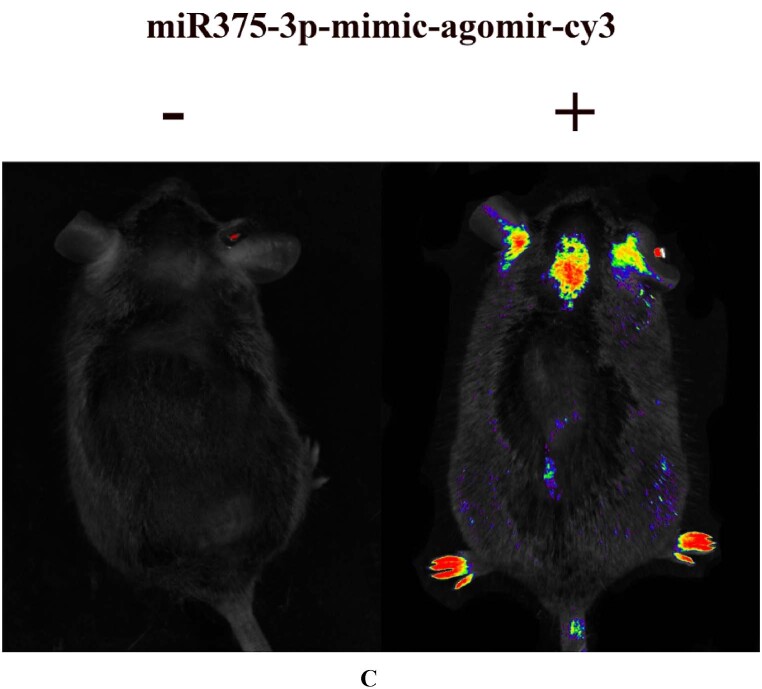
APP/PS1 transgenic mice tail veins were injected with 0.8 % physiological saline and miR-375-3p-mimic-agomir-cy3. After 24 h, the mice were **anesthetized**, and live imaging was conducted using a 633 nm channel.

The Morris water maze results revealed that the escape latency of the miR375-3p/AD group (Fig. 4D, blue line) was significantly shorter than AD and rapamycin/AD groups. Furthermore, the average escape latency during the 7-day experimental period was significantly reduced in miR375-3p/AD and rapamycin-miR375-3p/AD groups compared to the AD group (Fig. 4E, blue and green bars), while the rapamycin/AD group did not show a significant memory improvement (Fig. 4E, yellow bars). Moreover, based on 7th-day escape latency results, the miR375-3p/AD and rapamycin-miR375-3p/AD groups indicated significantly shorter escape latencies, approaching those of the wild-type group, in contrast to the AD and rapamycin/AD groups (Fig. 4F).

**Fig. 4D–G:**
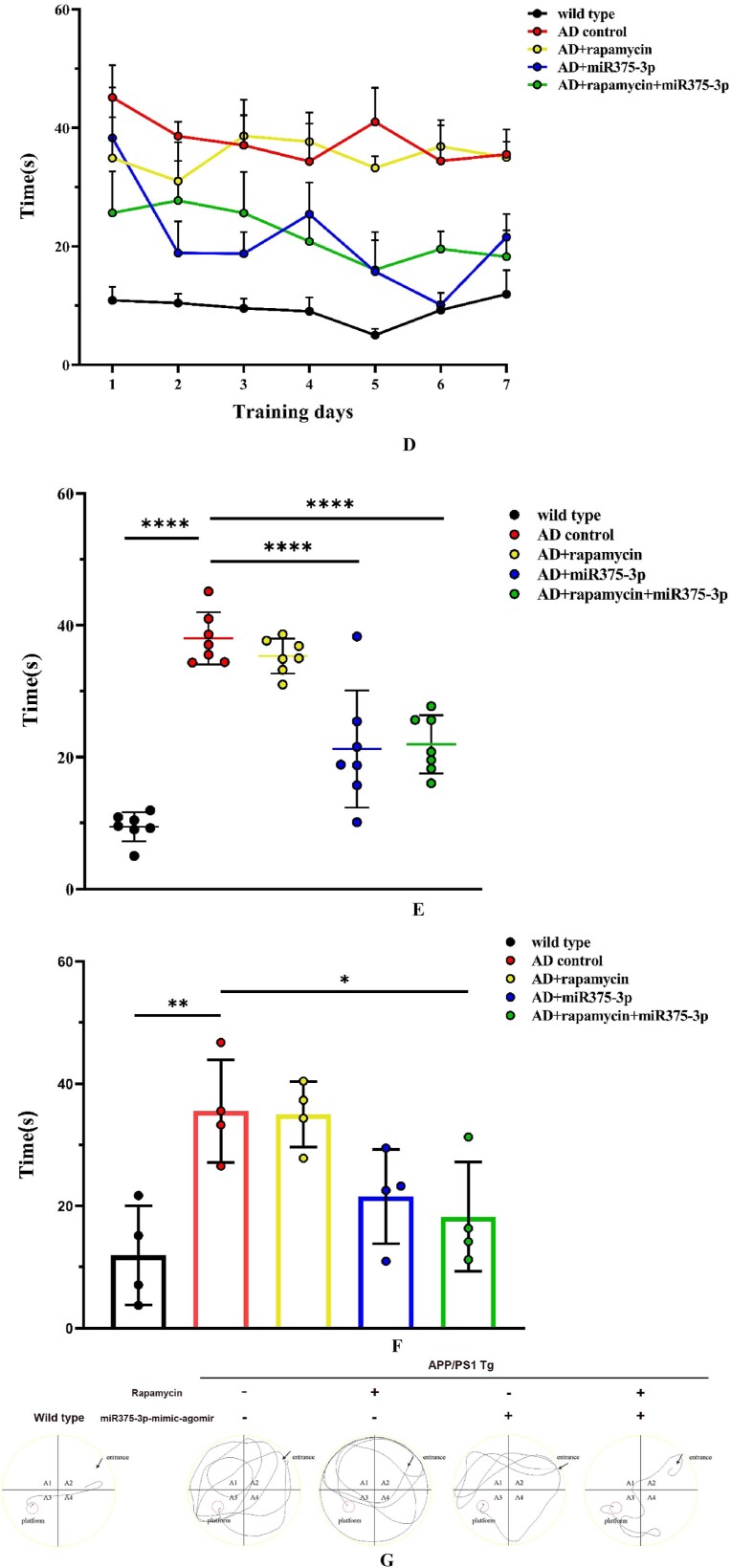
Morris Water Maze testing of memory in APP/PS1 transgenic mice. (D) The time curve indicates the time each group of mice took to reach the hidden platform during the experimental stages. (E) Cumulative escape latency for each group during the experimental stages. (F) Escape latency for each group on the final day of the experimental stage. (G) Representative images of probe test movement trajectories. (H–J) Y-Maze analysis to test the cognitive abilities in APP/PS1 transgenic mice.

The miR375-3p/AD and rapamycin-miR375-3p/AD groups selected shorter routes to find the hidden platform, displaying stronger search strategies compared to the AD and rapamycin/AD groups (Fig. 4G). Furthermore, the results from the Y maze experiments were similar to those from the Morris water maze experiments. Moreover, spontaneous alternation experiments in the Y maze revealed that Compared to AD control, the mice in the other four groups had stronger rationality in their exploratory sequence, indicating a better recovery of spatial cognitive abilities (Fig. 4H). In the novel arm exploration experiment, the miR375-3p/AD and rapamycin-miR375-3p/AD groups indicated longer exploration times and distances compared to the AD group (Fig. 4I, Fig. 4JI and J, blue and green bars), while the rapamycin/AD group (Fig. 4I, Fig. 4JI and J, yellow bars) showed no significant difference compared to the AD group. These data indicated that miR375-3p-mimic-agomir intervention or co-treatment with rapamycin can significantly alleviate cognitive impairment and memory deficits in APP/PS1 transgenic mice.

**Fig. 4H:**
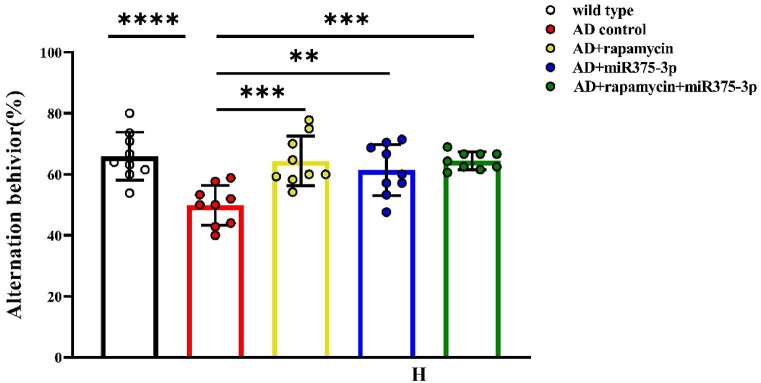
Percentage of correct alternations during the 10-min spontaneous alternation test.

**Fig. 4I:**
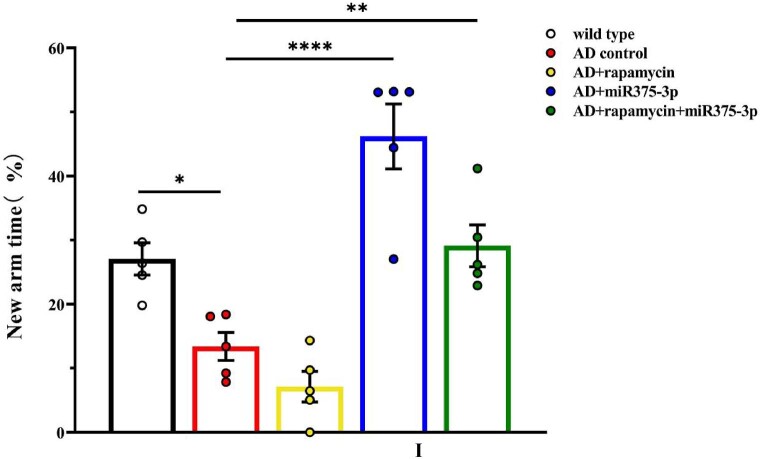
Time spent in the new arm exploration for each group.

**Fig. 4J:**
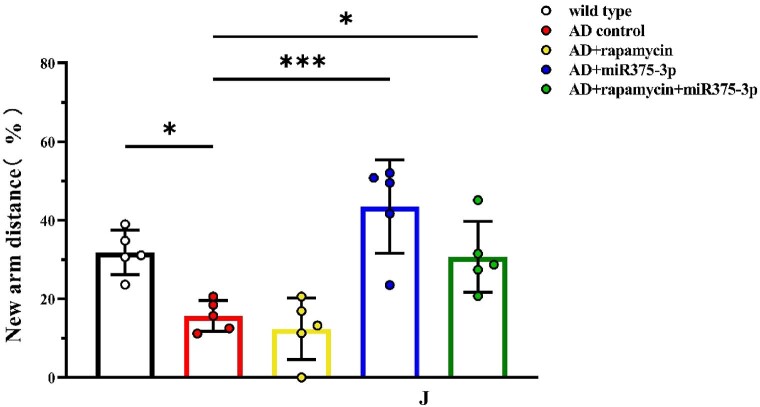
Distance traveled in the new arm exploration for each group (n = 5/group). All data are presented as the mean ± SEM of at least three independent experiments and were compared using one-way ANOVA with Tukey's multiple comparisons test. *∗p < 0.05, ∗∗p < 0.01, ∗∗∗p < 0.001, ∗∗∗∗p < 0.0001*.

### 3.6 The combination of rapamycin and miR375-3p-mimic-agomir reduces Aβ deposition in the brains of AD mice and repairs neural damage

Following behavioral analysis, mice were euthanized, and the brain's frontal and temporal lobes were collected for Western blot experiments. The hippocampal sections were prepared for immunohistochemistry and immunofluorescence staining. Immunohistochemical staining of the APP/PS1 Tg mice's hippocampal region revealed (Fig. 5A and B) that the AD group had the highest number of Aβ-positive plaques, which were spherical, dense, and had high brightness. Whereas the AD + rapamycin + miR375-3p and AD + miR375-3p groups had fewer Aβ-positive plaques, which were relatively dispersed. Among them, the AD + rapamycin + miR375-3p group had the fewest Aβ-positive plaques, while that of the AD + rapamycin group did not differ significantly from the AD group. These data indicated that the combined use of rapamycin and miR375-3p-mimic-agomir can most effectively reduce Aβ deposition in the brains of APP/PS1 Tg mice among all experimental groups. Immunohistochemical staining of the hippocampal region (Supplementary Figs. 1G and H) that rapamycin and miR375-3p-mimic-agomir did not affect extracellular Aβ generation in wild-type mice.

**Fig. 5A and B:**
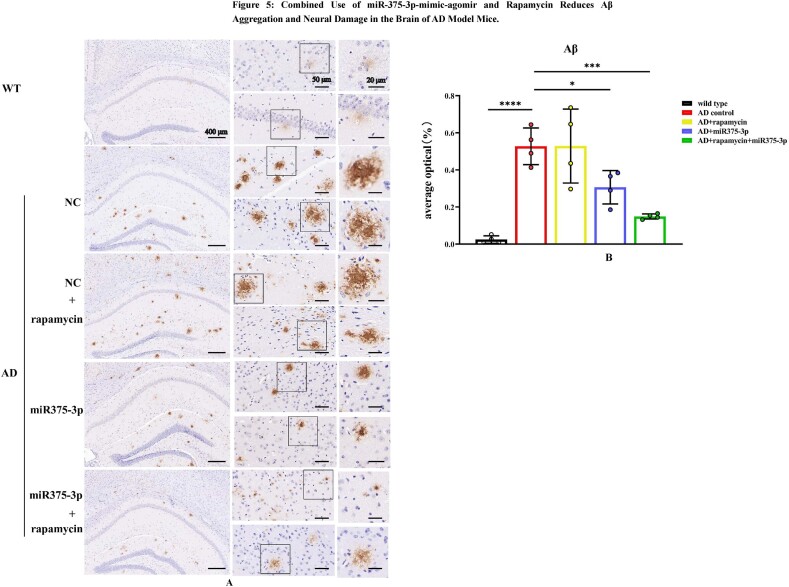
Immunohistochemical staining images indicating Aβ aggregation in the hippocampal region of APP/PS1 Tg mice brains. (A) Immunohistochemistry analysis of Anti-Aβ stained sagittal brain tissue sections acquired from whole-brain samples from each group of mice. (B) Image-Pro Plus 6.0 was used to quantitatively process and analyze Aβ-positive expression plaques in each group.

The number of Nissl bodies is an indicator of neural damage within the brain. Results from Nissl body staining experiments (Fig. 5C and D) indicated that the rapamycin-miR375-3p/AD and miR375-3p/AD groups had significantly increased number of Nissl bodies compared with the AD group. This suggested that neural damage was repaired. However, there was no significant difference between the rapamycin/AD and AD groups. Furthermore, the Tunel analysis validated these findings. The apoptosis rate in the brains of mice with co-treatment of rapamycin and miR375-3p-mimic-agomir was reduced from 63.24 ± 16.16 % to 25.7 ± 7.05 % compared to the AD group. When rapamycin or miR375-3p-mimic-agomir was used individually, the apoptosis rates were reduced to 35.73 ± 5.06 % and 35.95 ± 7.94 %, respectively (Fig. 5E and F). These results indicated that the co-treatment of rapamycin and miR375-3p-mimic-agomir can reverse cell apoptosis in the brains of APP/PS1 transgenic mice, and repair neural damage. Moreover, miR375-3p-mimic-agomir mono-treatment also indicated a similar effect, although it was not as effective as the co-treatment. The use of rapamycin alone had a positive impact on cell apoptosis in the brain but had no significant effect on the clearance of Aβ plaques and the restoration of Nissl bodies.

**Fig. 5C and D:**
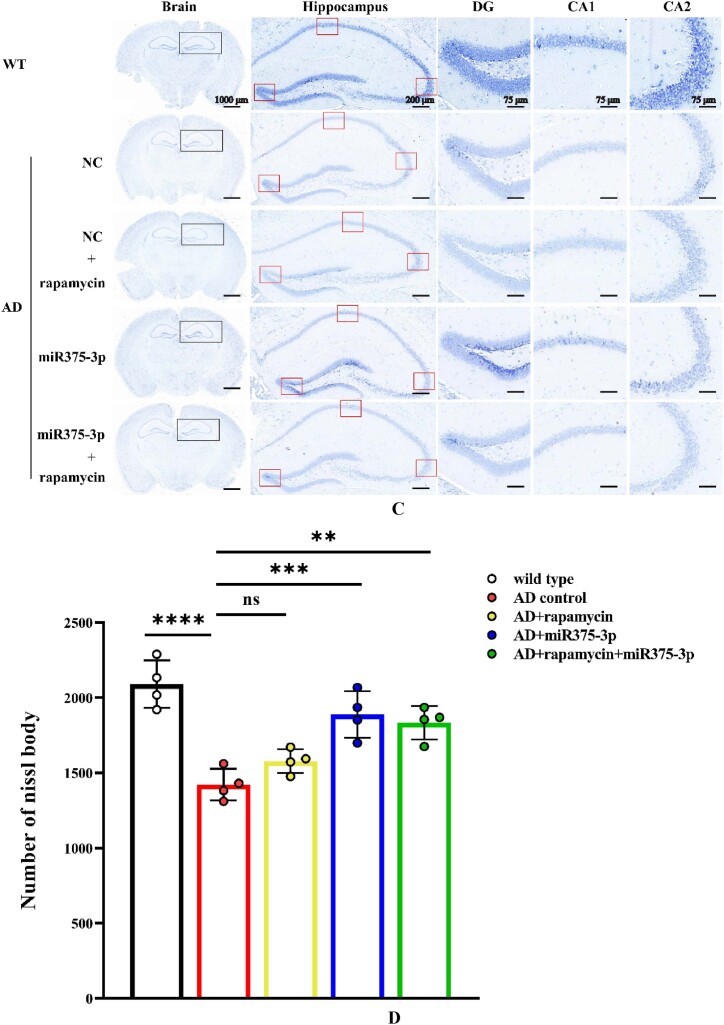
Nissl staining to assess the number of Nissl bodies in the brains of APP/PS1 transgenic mice. (C) The total number of Nissl bodies in the hippocampus and cerebral cortex after brain sections were stained with Nissl solution. (D) Cell counting was performed to determine the number of Nissl bodies in the cerebral cortex of each sample.

**Fig. 5E and F:**
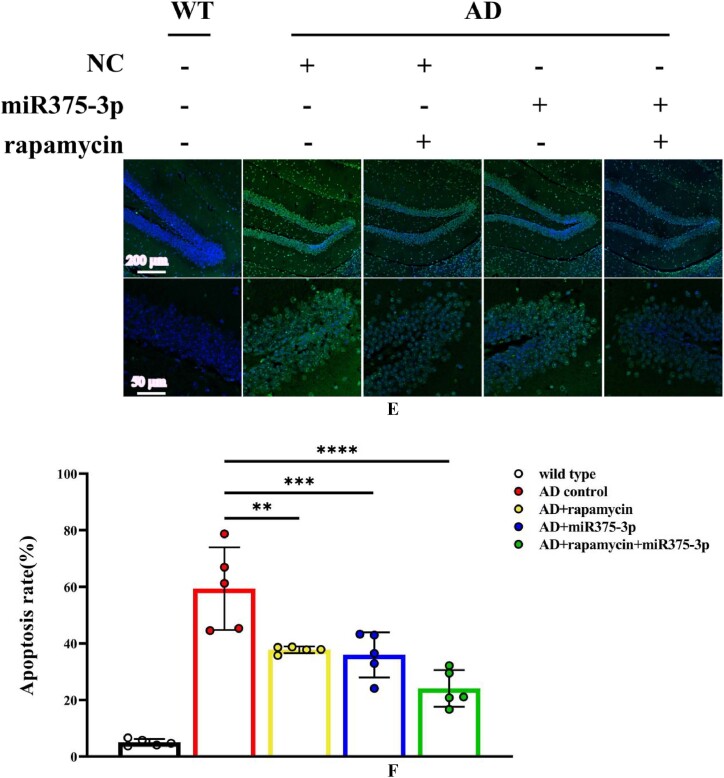
Tunel staining was performed to detect the rate of cell apoptosis in the hippocampal region of APP/PS1 Tg mice brains. (E) Brain sections were treated with a Tunel probe for 60 min, and results were analyzed using a laser confocal microscope with an Alexa Fluor 488 channel. (F) Image-Pro Plus 6.0 was used to quantify the fluorescence intensity in brain sections from each group to calculate the apoptosis rate. All data are presented as the mean ± SEM of at least three independent experiments and were compared using one-way ANOVA with Tukey's multiple comparisons test. *∗p < 0.05, ∗∗p < 0.01, ∗∗∗p < 0.001, ∗∗∗∗p < 0.0001*.

### 3.7 The combination of rapamycin and miR375-3p-mimic-agomir can reduce neuropathological changes at both transcription and translation levels in the AD mouse brain

In APP/PS1 Tg mice, single miR375-3p-mimic-agomir injection indicated similar results as observed in the co-treatment group. Western blot results from the frontal and temporal lobes showed that miR375-3p intervention blocked AD-related PS1 overexpression, APP degradation, CTFα, and β generation, as well as decreased BDNF protein levels, restored the expression levels of SYN and autophagy markers (Beclin1 and LC3B), Grp75, and Mfn2 (Fig. 6A and B). Furthermore, the western blot results from the frontal and temporal lobes of wild-type mice showed that miR375-3p intervention had no significant effect on the protein expression of Syn and Beclin1; however, inhibited LC3 processing. On this basis, rapamycin intervention had no significant effect on the expression of Syn and Beclin1 protein expression but could promote LC3B-II release. These results indicated that rapamycin could restore the down-regulation of autophagy induced by miR375-3p under normal physiological conditions. (Supplementary Fig. 1A and B).

**Fig. 6A and B:**
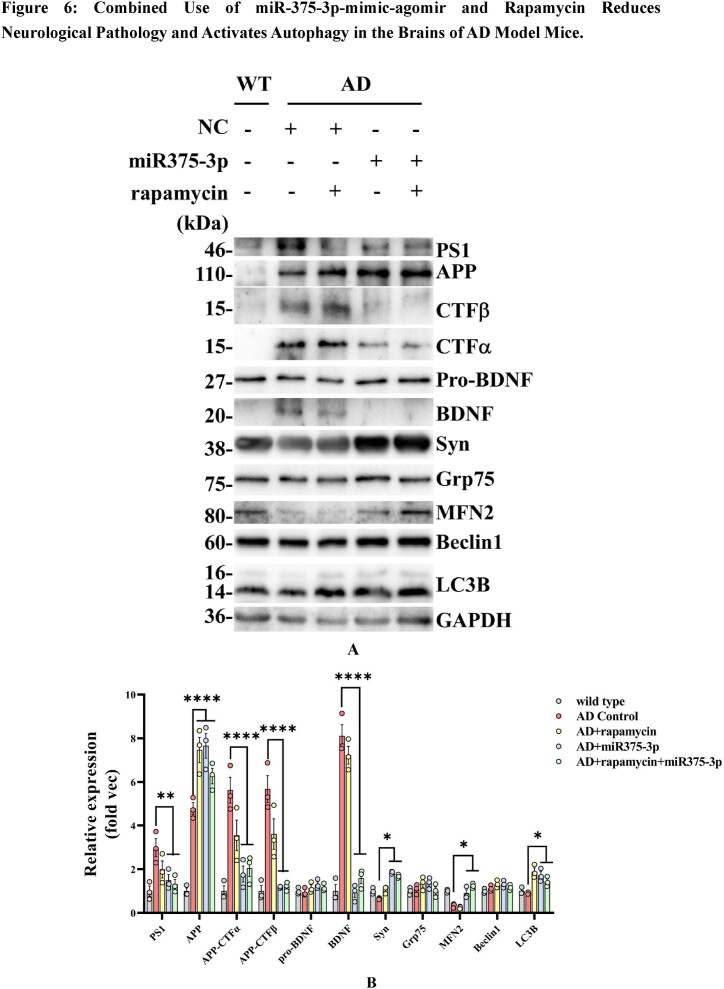
Western blot detection of protein expression in the parietal and temporal lobes of the brains of various groups of APP/PS1 Tg mice. (A) Protein expression of AD pathological markers, such as PS1, APP, CTFα, CTFβ, BDNF, and Syn, as well as autophagy markers including Mfn2, Beclin1, Grp75, and LC3B. (B) The grayscale values of each group were quantified using ImageJ. GAPDH was employed as an internal reference.

The hippocampal region immunofluorescence staining confirmed western blot results in APP/PS1 Tg mice. Compared to the AD group, the fluorescence intensity of pro-BDNF in the miR375-3p injection group and the rapamycin + miR375-3p combined injection group was significantly increased, and the positive expression morphology was closer to that of wild-type mice (Fig. 6C and D). These results were consistent for Syn (Fig. 6E and F). Moreover, the protein expression of Beclin1 (Fig. 6G and H) and LC3B (Fig. 6I and J) significantly increased, while that of PS1 significantly decreased (Fig. 6K and L) in the rapamycin, miR375-3p, and rapamycin + miR375-3p combined groups compared to the AD group. Immunohistochemical staining results showed that compared to the AD group, the positive expression rate of Beclin-1 in the AD + rapamycin + miR375-3p group significantly increased, but there was no statistical difference between the AD + rapamycin and AD + miR375-3p groups (Fig. 6M and N). Immunohistochemical results from the hippocampal region of wild-type mice showed that compared to the control group, there was no significant difference in Beclin1 positive expression in the rapamycin, miR375-3p, and combined treatment groups (Supplementary Fig. 1K and L). The LC3B expression was downregulated in the miR375-3p group, while it was recovered in the rapamycin and co-treatment groups, which was consistent with Western blot results (Supplementary Fig. 1I and J).

**Fig. 6C–L:**
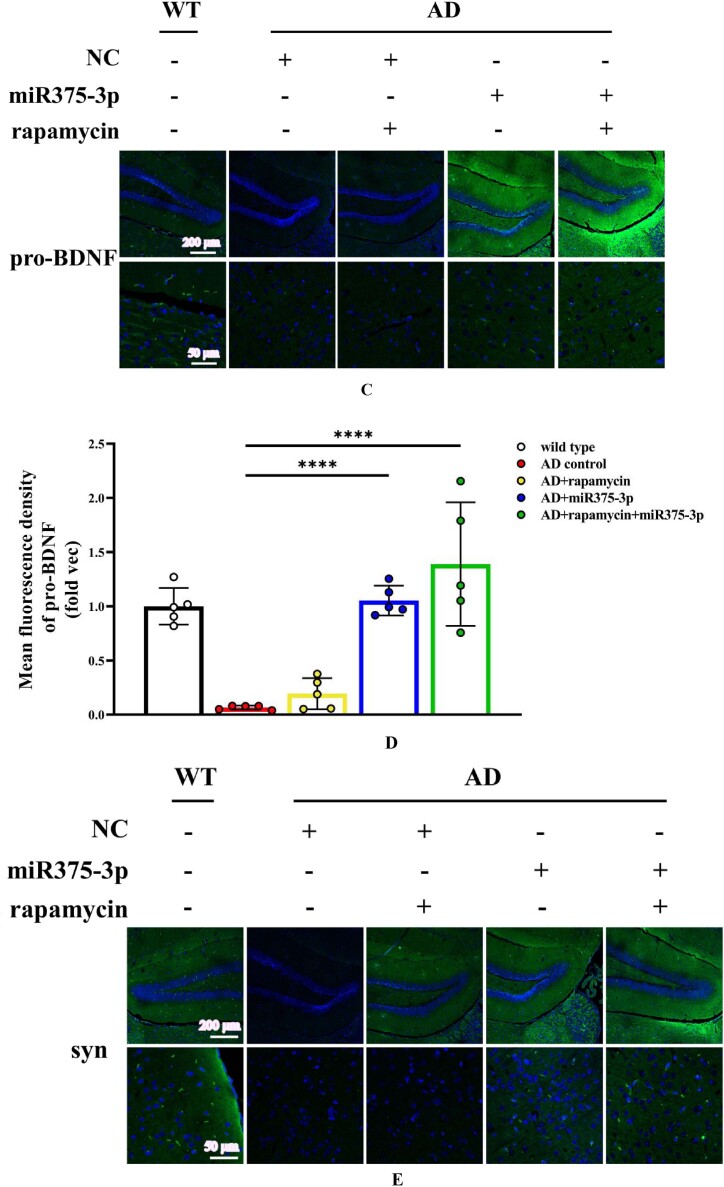

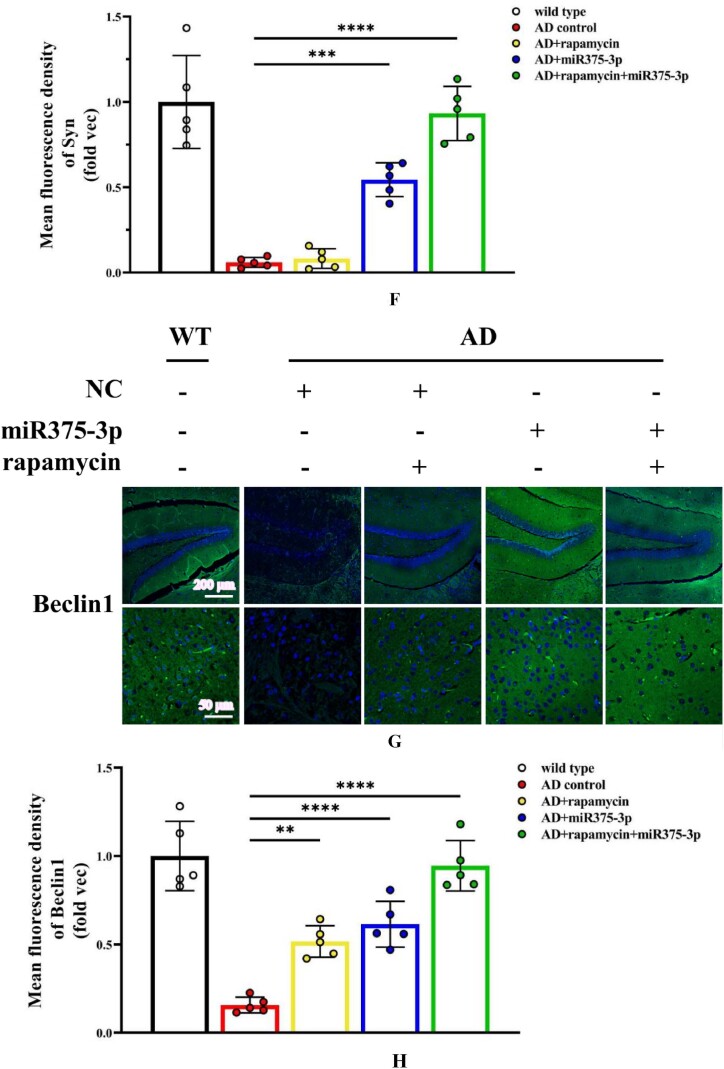

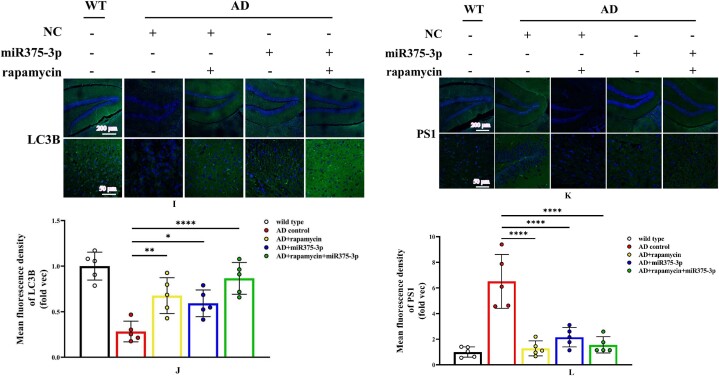
Immunofluorescence staining was performed to detect the protein expression levels of BDNF and Syn in the hippocampal region of APP/PS1 Tg mice. (C, E, G, I, and K) Brain sections from each group were stained with Anti-BDNF (C), Anti-Syn (E), Anti-Beclin1 (G), Anti-LC3B (I), and Anti-PS1 antibodies (K) as well as FITC-conjugated secondary antibodies. (D, F, H, J, and L) The fluorescence intensity of each group was quantified and analyzed using image-pro-plus 6.0. The results were normalized relative to the expression levels of BDNF, Syn, Beclin1, LC3B, and PS1 in wild-type mice.

**Fig. 6M and N:**
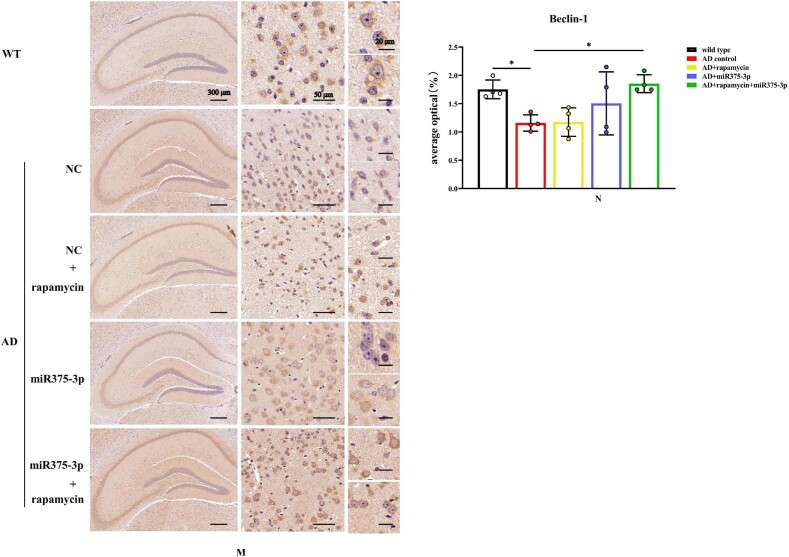
Immunohistochemistry was performed to detect the protein expression of Beclin1 in the hippocampal region of APP/PS1 Tg mice. (M) Brain sections were stained using Anti-Beclin1 for immunohistochemistry. (N) Image-Pro Plus 6.0 was used to quantify and analyze the positivity rate of Beclin1 in each group.

qRT-PCR results showed that co-treatment of rapamycin and miR375-3p-mimic-agomir as well as mono-treatment of miR375-3p-mimic-agomir inhibited the transcription levels of PS1 and promoted the expression of BDNF and Syn, while the expression level of APP approached that of wild-type mice. Rapamycin mono-treatment inhibited PS1 and APP expressions but indicated no significant difference in other indicators compared to the AD group (Fig. 6O). Furthermore, in autophagy regulation, compared to the AD group, co-treatment of rapamycin and miR375-3p-mimic-agomir as well as mono-treatment of miR375-3p-mimic-agomir promoted the expression of LC3B, Beclin-1, Mfn2, and Pink-1 at the transcription level, indicating the stimulation of macroautophagy and mitochondrial autophagy. Rapamycin mono-treatment increased the expression of LC3B; however, no significant effect was observed on Mfn2, Pink-1, and Beclin-1 (Fig. 6P). Fluorescence quantitative PCR results from wild-type mice showed that miR375-3p intervention had no significant effect on the transcriptional level of Beclin1, but had a certain inhibitory effect on LC3B expression, and rapamycin addition effectively alleviated LC3B downregulation (Supplementary Fig. 1S).

**Fig. 6O and P:**
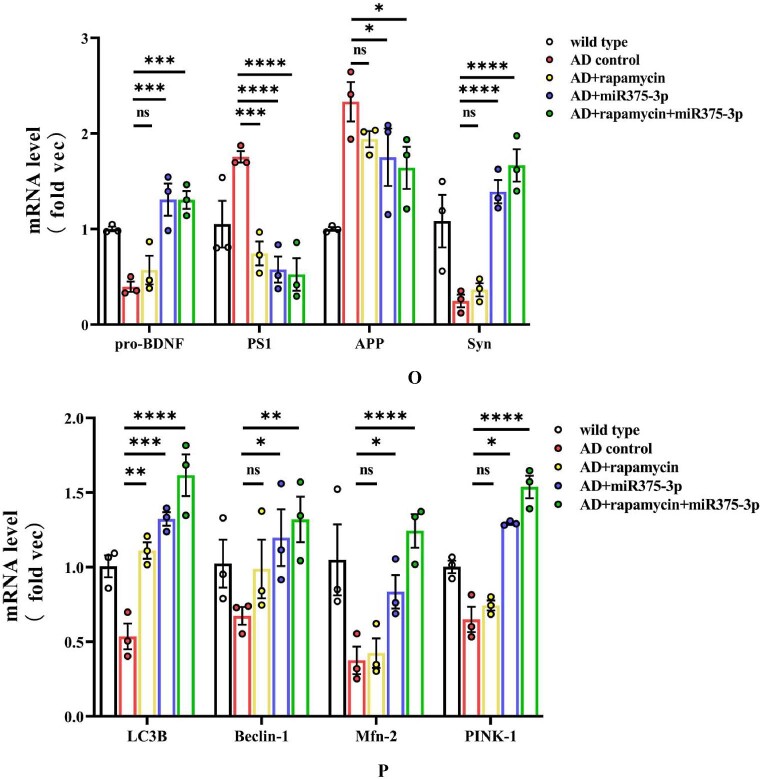
qRT-PCR analysis of the mRNA expression levels in the mice. (O) qRT-PCR results of mRNA levels of AD markers: APP, PS1, BDNF, and Syn. Briefly, total RNA was extracted from the blood of each mouse group, reverse-transcribed, and then used as a template for qRT-PCR. (P) qRT-PCR results of mRNA levels of autophagy markers: LC3B, Beclin1, Pink1, and MFN2. All data are presented as the mean ± SEM of at least three independent experiments and were compared using one-way ANOVA with Tukey's multiple comparisons test. *∗p < 0.05, ∗∗p < 0.01, ∗∗∗p < 0.001, ∗∗∗∗p < 0.0001*.

In summary, in AD, both rapamycin and miR375-3p-mimic-agomir can affect the neurological pathology of APP/PS1 Tg mice at transcription and translation levels, and their co-treatment can inhibit the neuro-pathological features of APP/PS1 Tg mice, promote synaptic formation, and repair autophagy disorders. During normal physiological conditions, miR375-3p overexpression can inhibit autophagy flux at the transcription and translation levels in wild-type mice, while the addition of rapamycin can effectively alleviate this condition.

### 3.8 miR375-3p promotes autophagy in AD mice by inhibiting mTOR phosphorylation

To further elucidate the mechanism of action of miR375-3p, the association of the mTOR pathway with AD was examined. Western blot results showed that after Aβ_25-35_ treatment for different periods, the phosphorylation levels of mTOR (S2448) and p70S6 (T389) in SH-SY5Y cells significantly increased, while that of pAKT (S473) was not significantly affected at 18 and 24 h. Furthermore, a slight increase in AKT phosphorylation activity was observed at 48 h (Fig. 7A and B). Moreover, Aβ_25-35_ treatment for 24 h induced mTOR and p70S6 phosphorylation, ULK1 dephosphorylation, and inhibited ATG14 expression (Fig. 7C lane 3). Rapamycin blocked the phosphorylation of p70S6 and inhibited the activity of mTORC1 by binding to FKBP12 [34]. Therefore, when rapamycin was used to treat Aβ_25-35_-induced SH-SY5Y cells, dephosphorylation of mTOR and p70S6 was observed, where ULK1 (S638) was only slightly dephosphorylated (Fig. 7C lane 7). In addition, after co-treatment of miR375-3p-mimic and rapamycin, phosphorylation of p-mTOR and pAKT, and dephosphorylation of ULK1 was still detected; however, the phosphorylation of p70S6 was blocked, and ATG14 expression was restored (Fig. 7C lane 6). However, after Aβ_25-35_ treatment, co-treatment of miR375-3p-mimic and rapamycin could block the phosphorylation of mTOR and AKT (Fig. 7C lane 8).

**Fig. 7A and B:**
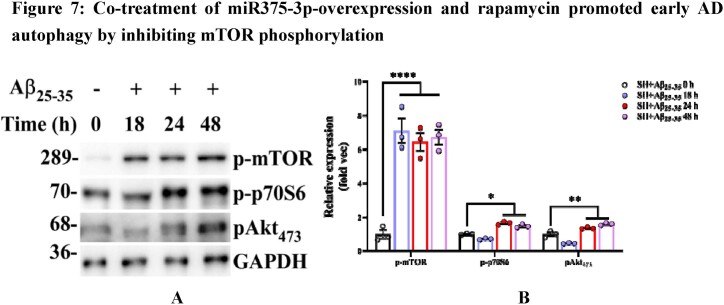
Western blot analysis was conducted to assess the activation of mTOR pathway-related proteins under different Aβ_25-35_ induction time conditions. (A) Phosphorylation activation status of mTOR, p70S6, and AKT in SH-SY5Y cells. (B) Grayscale values of each group were quantified and analyzed using ImageJ. GAPDH was used as an internal reference.

**Fig. 7C and D:**
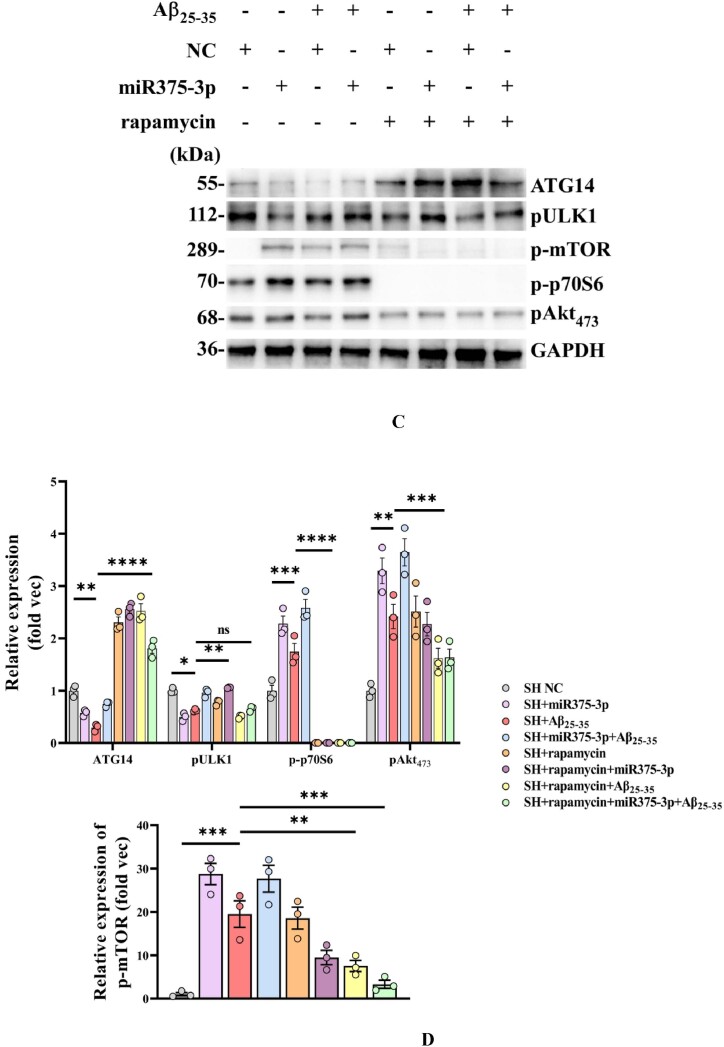
Western blot evaluation of mTOR pathway-related protein expression in various groups of SH-SY5Y cells. (C) Protein expression of phosphorylated mTOR, ULK1, p70S6, and AKT in SH-SY5Y cells. (D) Grayscale values of each group were quantified using ImageJ with GAPDH as an internal reference.

Western blot results from the frontal and temporal lobes showed that the phosphorylation level of Akt in APP/PS1 Tg mice was lower than that in wild-type mice. Furthermore, in the AD + rapamycin + miR375-3p group, p-mTOR was inhibited, ULK-1 phosphorylation levels were significantly increased, and ATG14 expression showed no significant difference compared to the AD group (Fig. 7E and F). These results were consistent with the data acquired from the immunofluorescence, which indicated that compared to the AD group, ATG14 protein expression was significantly downregulated in the AD + rapamycin and AD + miR375-3p groups, whereas there was no statistical difference in the AD + rapamycin + miR375-3p group (Fig. 7G and H), indicating that the combined action of rapamycin and miR375-3p-mimic-agomir can inhibit mTOR phosphorylation in the brain of APP/PS1 Tg mice, thereby affecting downstream autophagy. Moreover, the results of the western blot showed that compared to the control wild-type group, miR375-3p overexpression could activate mTOR phosphorylation and promote downstream p70S67 phosphorylation, with no significant effect on AKT phosphorylation. Whereas rapamycin blocked the phosphorylation of mTOR and p70S67, while AKT phosphorylation levels remained unchanged, indicating that mTORC1 activity was inhibited, while mTORC2 remained unaffected (Supplementary Fig. 1A and C).

**Fig. 7E and F:**
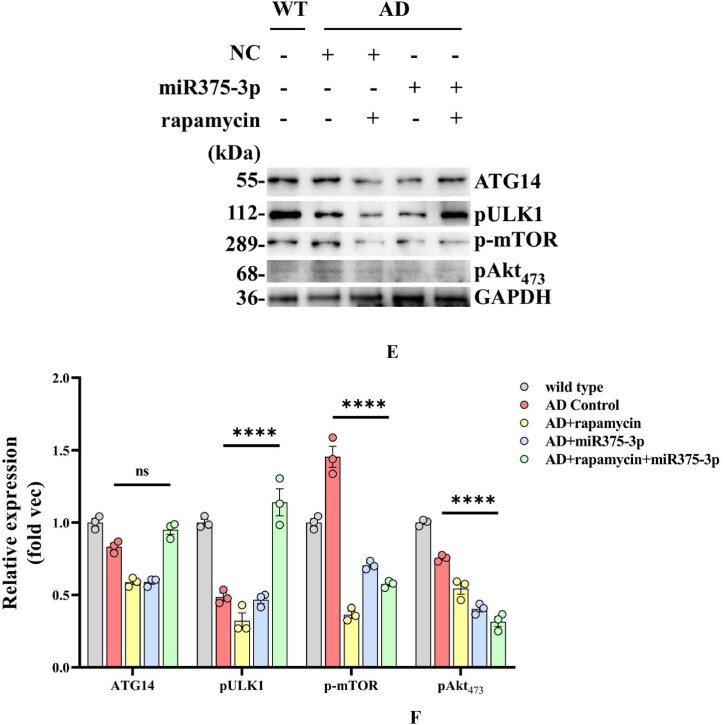
Western blot analysis was conducted to examine the activation of mTOR pathway-related proteins in the parietal and temporal lobes of the brains of APP/PS1 Tg mice. (E) Protein expression levels of phosphorylated mTOR, ULK1, AKT, and ATG14. (F) Grayscale values of each group were quantified and analyzed using ImageJ. GAPDH was used as an internal reference.

**Fig. 7G and H:**
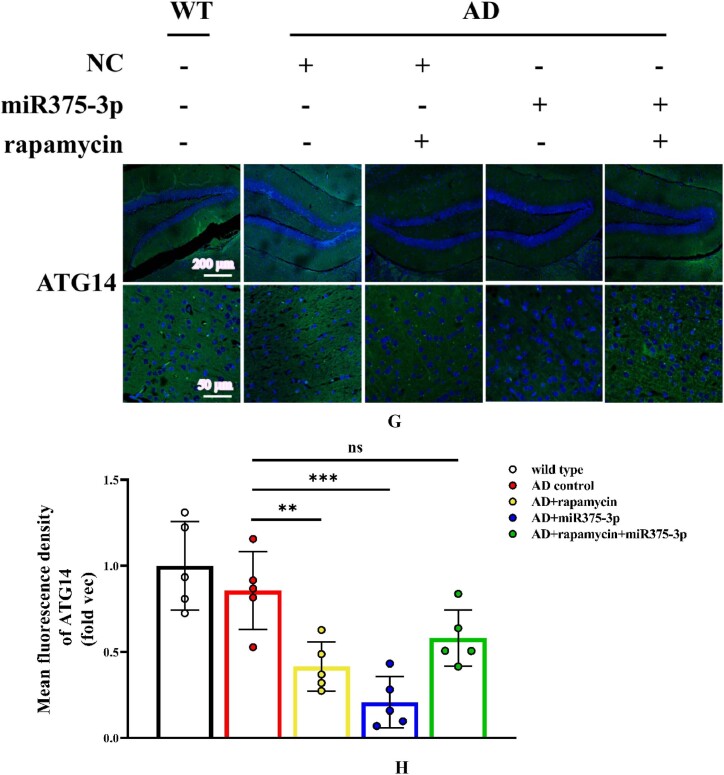
Immunofluorescence staining images indicate the protein expression levels of ATG14 in the hippocampal region of APP/PS1 Tg mice. (G) Tissue sections were stained using Anti-ATG14 and FITC-conjugated secondary antibodies. (H) The fluorescence intensity of each group was quantified and analyzed using image-pro-plus 6.0. The results were normalized relative to the expression levels of ATG14 in wild-type mice. All data are presented as the mean ± SEM of at least three independent experiments and were compared using one-way ANOVA with Tukey's multiple comparisons test. *∗p < 0.05, ∗∗p < 0.01, ∗∗∗p < 0.001, ∗∗∗∗p < 0.0001*.

### 3.9 Rapamycin and miR375-3p-mimic can regulate microglial activation

In early-stage AD, microglia often play a neuroprotective role by promoting the clearance of Aβ and Tau proteins [35]. According to bioinformatics predictions, M2-polarized microglia marker, CD68, is a target gene of miR375-3p (Fig. 8A) and its overexpression in SH-SY5Y cells (Fig. 8B and C) and wild-type mice (Fig. 8D and E) inhibited the expression of the CD68 protein, consistent with cell immunofluorescence staining results (Fig. 8F and G; lanes 1 and 2). However, during Aβ_25-35_ treatment, miR375-3p-mimic promoted CD68 protein expression in SH-SY5Y cells (Fig. 8F and G; lanes 3 and 4). To further clarify the role of microglial cells, whether astrocytes are associated was determined. Western blot results showed that miR375-3p-mimic had little effect on GFAP, indicating the non-significant influence of astrocytes on this process. Furthermore, after Aβ_25-35_ induction, both rapamycin and miR375-3p-mimic promoted the expression of M2-polarized microglia marker - CD80 and quiescent microglia markers - Iba1 in SH-SY5Y cells, and the effects were more pronounced in the co-treatment group (Fig. 8H and I; lanes 3/4 and 7/8). Western blotting of HMC3 cells showed that miR375-3p overexpression could promote the expression of CD80 and CD68, and the expression level of Iba1 was down-regulated (Supplementary Fig. 2A and B). These results indicate that miR375-3p can induce M1 and M2 polarization in microglial cells. Meanwhile, overexpression of miR375-3p combined with rapamycin can form an environment that induces microglia activation *in vitro*.

**Fig. 8A:**
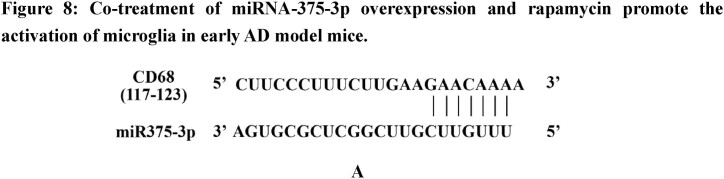
Prediction of the interaction site between miR-375-3p and CD68 protein.

**Fig. 8B–E:**
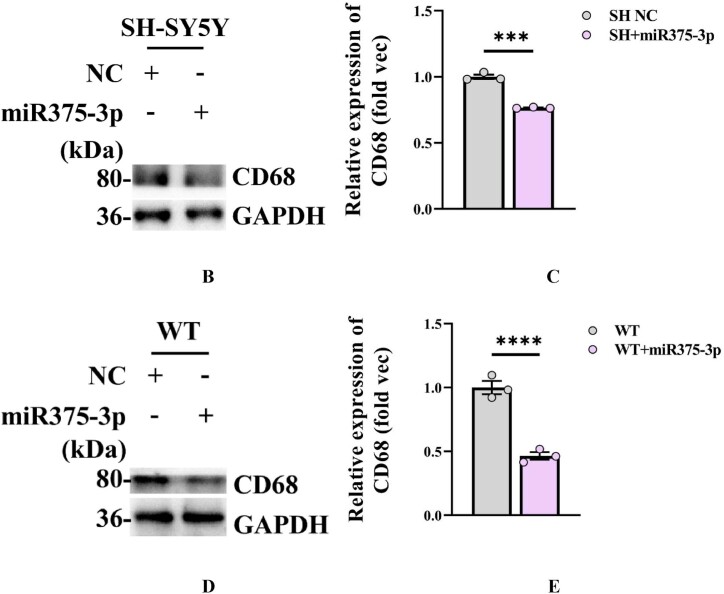
Western blot analysis was performed to detect CD68 protein expression in SH-SY5Y cells (B) as well as in the parietal and temporal lobes of the brains of wild-type mice after (D) miR375-3p overexpression. Grayscale values of CD68 in each group were quantified using ImageJ. GAPDH levels in SH-SY5Y cells (C) and wild-type mice (E) were assessed and set as an internal reference.

**Fig. 8F and G:**
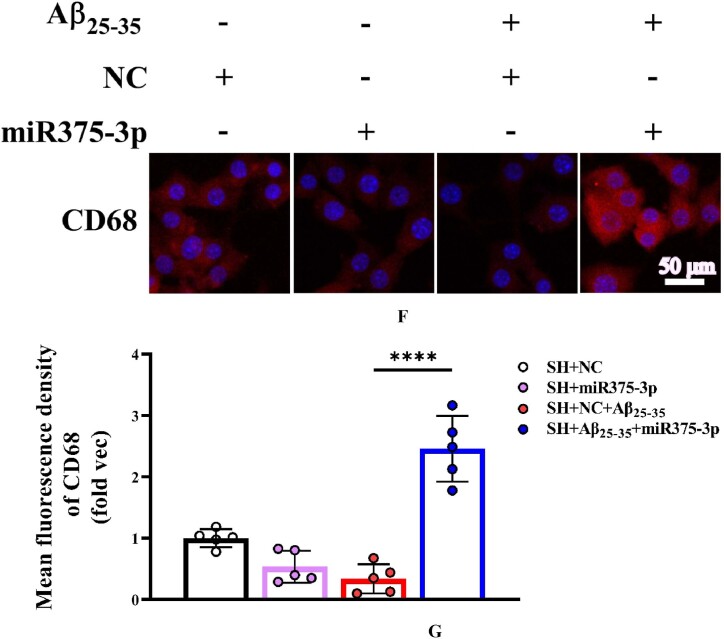
Immunofluorescence staining was conducted to detect the protein expression levels of CD68 in various groups of SH-SY5Y cells. (F) Staining was performed using Anti-CD68 and PE-conjugated secondary antibodies (400 × ); Scale bar = 50 μm. (G) The fluorescence intensity of each group was quantified and analyzed using image-pro-plus 6.0. The results were normalized relative to the expression levels of CD68 in SH + NC as the folder.

**Fig. 8H and I:**
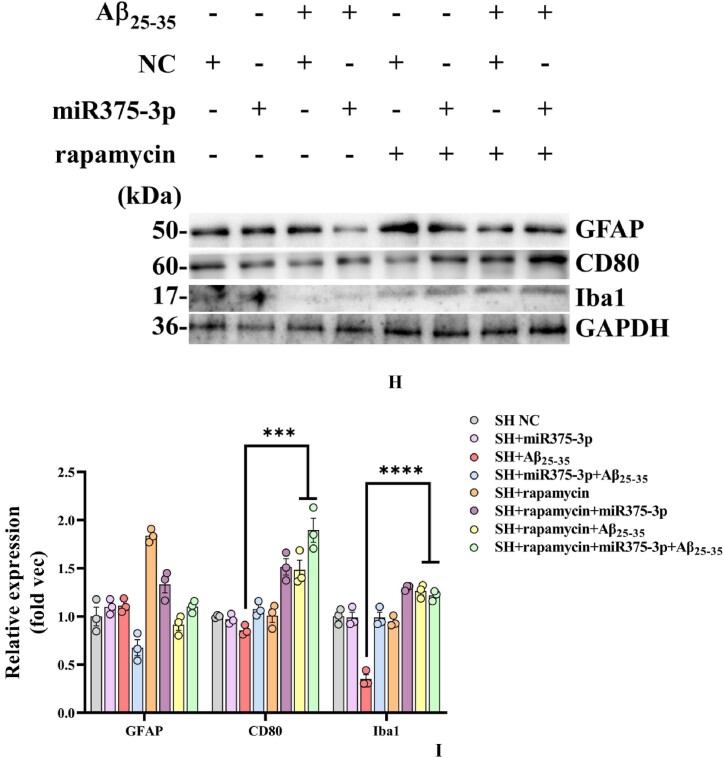
Western blot analysis was conducted to evaluate the expression of glial cell markers in various groups of SH-SY5Y cells. (H) Protein expression levels of CD80, Iba1, and GFAP. (I) Grayscale values of each group were quantified and analyzed using ImageJ. GAPDH was employed as an internal reference.

In APP/PS1 Tg mice, western blot results showed that the protein expression of CD80, Iba1, and CD68 in the AD group was lower than those in the WT group (Fig. 8J and K; lanes 1 and 2). However, after rapamycin or miR375-3p-mimic-agomir intervention, their expression significantly increased (Fig. 8J and K; lanes 3 and 4), and the co-treatment of rapamycin and miR375-3p-mimic-agomir had a more pronounced effect (Fig. 8J and K; lanes 5), indicating that the addition of rapamycin and miR375-3p-mimic-agomir could simultaneously induce M1 and M2 polarization of microglial cells. These data were consistent with immunofluorescence results indicating that compared to the AD group, the fluorescence intensity of CD80 and Iba1 expression was significantly increased in the three drug-treated groups, with the AD + rapamycin + miR375-3p group showing the most pronounced upregulation (Fig. 8L–O). Furthermore, immunohistochemical results further validated these data, with significantly higher positive expression rates of CD80 and Iba1 in the AD + miR375-3p, rapamycin, and AD + rapamycin + miR375-3p groups than in the AD experimental group (Fig. 8P–S). qRT-PCR results showed that rapamycin and miR375-3p-mimic-agomir promoted the transcriptional expression of CD68, CD80, and Iba1; however, significantly downregulated pro-inflammatory factor Interleukin-6 (IL-6) in the AD + rapamycin + miR375-3p group (Fig. 8T). This suggests that although co-treatment of rapamycin with miR375-3p-mimic-agomir activates M1 polarization, the corresponding inflammatory response is not exacerbated. TMEM119, Interleukin-1β (IL-1β), and CD206 are markers of resting, M1-type, and M2-type microglia, respectively. In addition, ELISA revealed that the expression of TMEM119 and CD206 antigens was upregulated while that of IL-1β was decreased in AD + miR375-3p and AD + rapamycin + miR375-3p groups compared with the AD group and all with statistical differences. The levels of IL-1β antigens in the rapamycin group showed no significant difference, while the level of TMEM119 and CD206 increased, which was consistent with qRT-PCR results (Fig. 8U–W).

**Fig. 8J and K:**
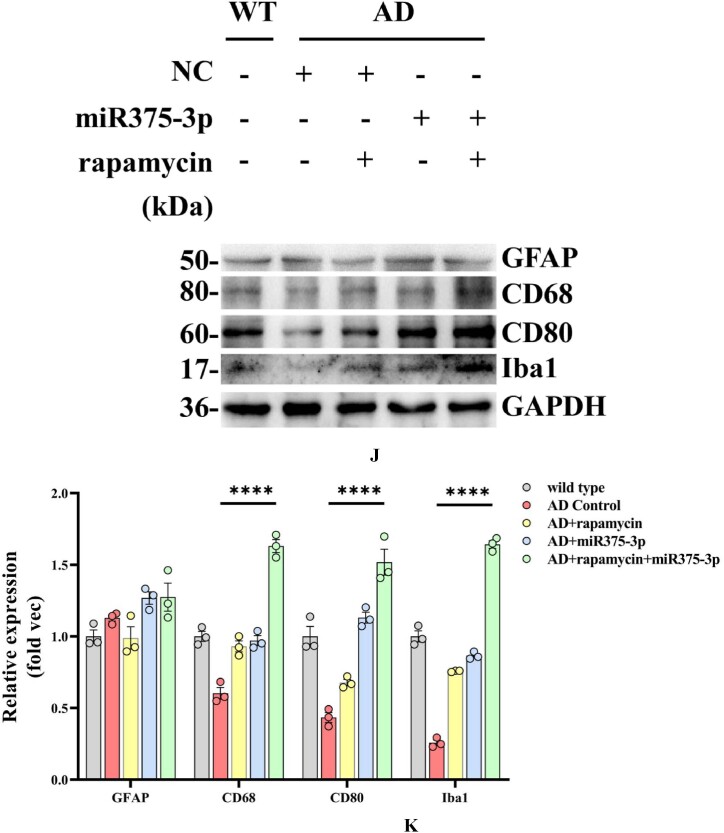
Western blot analysis was performed to assess the expression of glial cell marker proteins in the parietal and temporal lobes of the brains of APP/PS1 Tg mice. (J) Protein expression levels of GFAP, CD80, CD68, and Iba1. (K) Grayscale values of each group were quantified and analyzed using ImageJ. GAPDH was employed as an internal reference.

**Fig. 8L–O:**
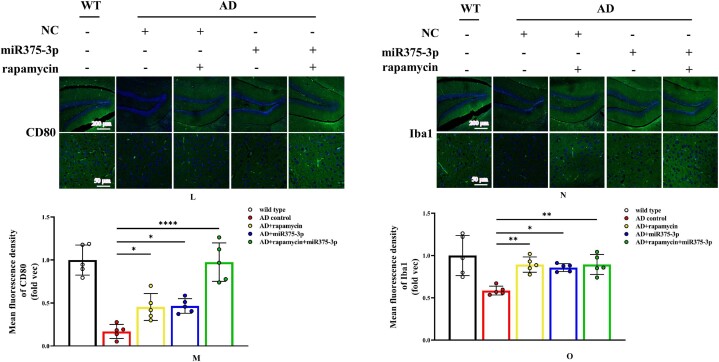
Immunofluorescence staining was conducted to detect the protein expression levels of CD80 and Iba1 in the hippocampal region of APP/PS1 Tg mice. (L and N) Staining was performed using Anti-CD80 (L) and Anti-Iba1 (N) antibodies and FITC-conjugated secondary antibodies (100 × , 400 × ), scale bar: 200 μm, 50 μm. (L and N) The fluorescence intensity of each group was quantified using image-pro-plus 6.0 and analyzed, with results displayed relative to the expression levels of CD80 (M) and Iba1 (O) in wild-type mice as the folder.

**Fig. 8P–S:**
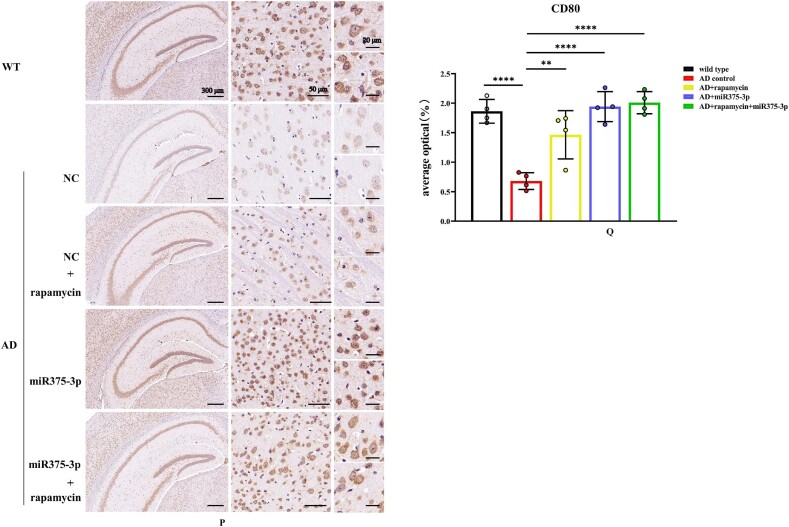

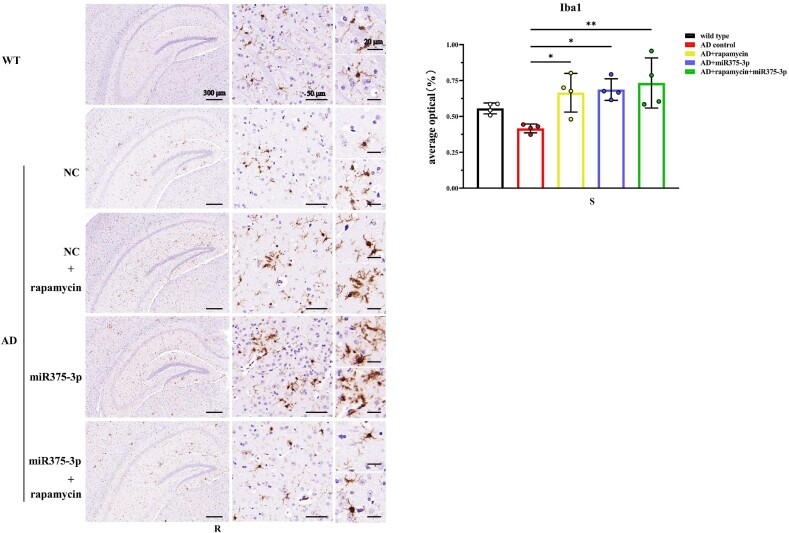
Immunohistochemical staining images indicating the distribution and positive expression of CD80 and Iba1 in the hippocampal region of APP/PS1 Tg mice. (P and R) Brain tissue sections were stained using Anti-CD80 (P) and Anti-Iba1 (R) antibodies for immunohistochemistry. (Q and S) The positive expression patches of CD80 (Q) and Iba1 (S) in each group were quantified and analyzed using image-pro-plus 6.0.

**Fig. 8T:**
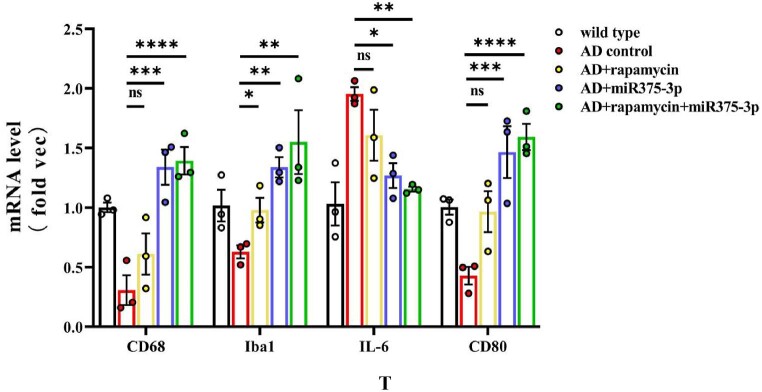
qRT-PCR to measure the mRNA expression levels of CD68, CD80, Iba1, and IL-6 in the mice.

**Fig. 8U–W:**
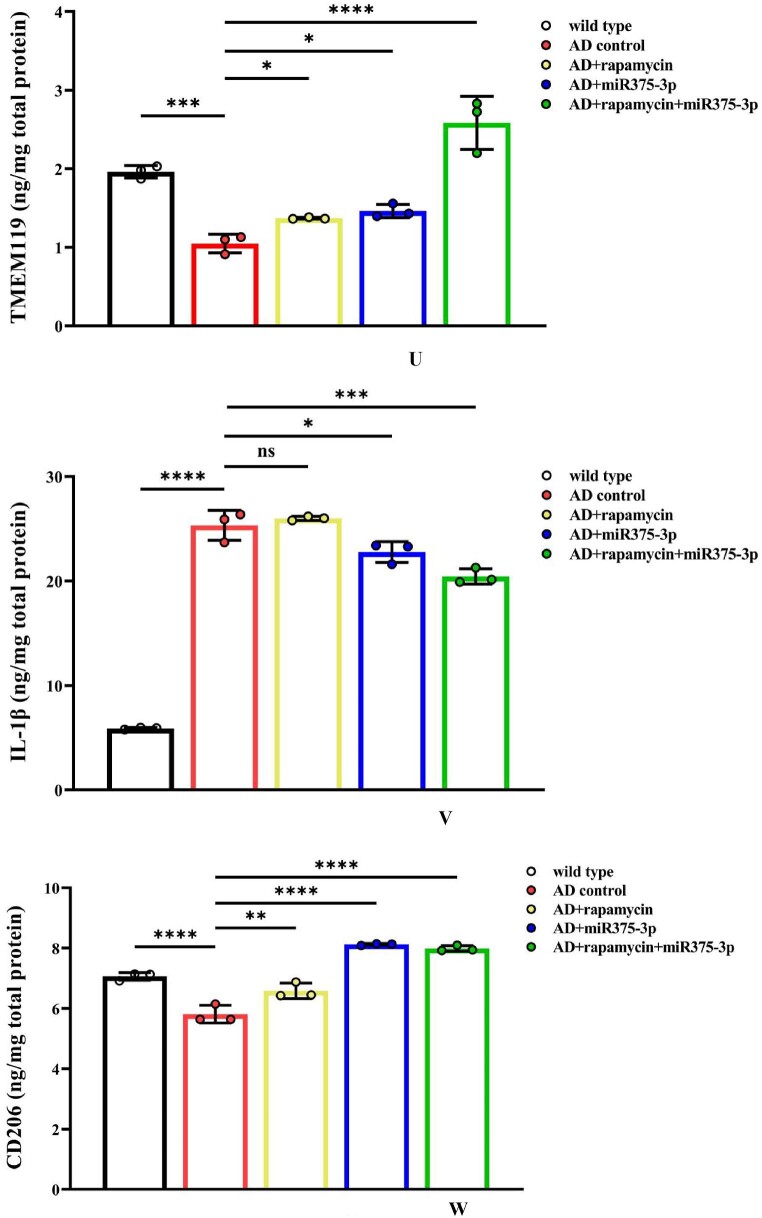
Elisa was performed to detect the protein levels of TMEM119 (U), IL-1β (V), and CD206 (W) in the brains of each group of mice. For each group, three replicates were set. Standard curves were drawn using Origin8.0. Data were summarized and analyzed using image-pro-plus 6.0.

In wild-type mice, western blot results from the frontal and temporal lobes showed that overexpression of miR375-3p can promote the protein expression of Iba1 and CD80, while downregulating the expression of CD68. Addition of rapamycin on this basis upregulated the expression of CD68, while the expression of GFAP, CD80, and Iba1 remained largely unchanged (Supplementary Fig. 1A and 1D). Immunohistochemical results from the hippocampal region showed that compared to the negative control group, overexpression of miR375-3p could promote the positive expression of Iba1 and CD80, and downregulate the positive expression of CD68. The expression of CD68 was reversed after the addition of rapamycin, with no significant effect on CD80 and Iba1 (Supplementary Fig. 1M, 1N, 1O, 1P, 1Q, 1R). The results of fluorescent quantitative PCR were also consistent (Supplementary Figure 1T).

The above experimental results indicated that rapamycin and miR375-3p-mimic treatments can induce the stress state of microglial cells as well as M1 and M2 polarization in Aβ_25-35_-induced cells. *In vivo,* experiments show that rapamycin and miR375-3p-mimic can promote the early activation of microglial cells in the brain of APP/PS1 Tg mice and exert neuroprotective functions. This was evident from the increased M2-type microglial cell activation and the stable expression of inflammatory factors. Furthermore, in wild-type mice, rapamycin and miR375-3p-mimic mainly promoted the activation of resting and M1-type microglial cells, while no significant effect was observed on M2-type microglial cell activation.

## 4 Discussion

The exploration of pathogenic mechanisms and treatment strategies has always been a focal point in AD research. It has been indicated autophagy dysregulation and microglial cell activation are the hallmark events in AD progression [[36], [37], [38]]. This study initially employed a bioinformatics approach to analyze the AD sample dataset identified differential expression of miRNA-375-3p and predicted its target proteins, including PS1, BDNF, Syn, ATG14, and CD68. Furthermore, Experimental evidence revealed that miR375-3p in AD attenuates PS1 expression, improves the autophagic microenvironment, enhances mitochondrial function, reduces oxidative stress, and activates microglial cells.Moreover, miR375-3p-mimic-agomir mono-treatment or co-treatment of rapamycin and miR375-3p-mimic-agomir effectively restored Nissl body damage in the brains of APP/PS1 transgenic (TG) mice (Fig. 5C and D), primarily protected hippocampal neurons in APP/PS1 transgenic mice, especially in the dentate gyrus (DG) (Fig. 5C), which is essentially involved in memory formation. Damage to the DG region affects most hippocampus-dependent memory functions, while the remaining regions (CA1-CA3) can function independently under certain conditions. Furthermore, miR375-3p-mimic-agomir mono-treatment or the co-treatment effectively eliminated intracellular and extracellular Aβ aggregation (Figures A and B), and improved memory deficits in APP/PS1 transgenic mice. In addition, the co-treatment exhibited superior efficacy. However, mono-treatment of rapamycin, while influencing autophagy, did not significantly improve memory in APP/PS1 transgenic mice (Fig. 4D–G, Fig. 4H, Fig. 4I, Fig. 4J, Fig. 5C and DD–J).

In AD mice, miR375-3p-mimic-agomir successfully penetrated the brain *via* the blood-brain barrier (Fig. 4C). Although bioinformatics predicted various target proteins, it was revealed that miR375-3p was most crucial for PS1, whereas rapamycin had minimal effect on PS1 expression. Considering bioinformatics predictions, ATG14 plays an important role in the autophagy pathway, and we need to clarify the role of autophagy in the mechanism of miR375-3p′s impact on AD. Here, it was observed that the co-treatment of miR375-3p and rapamycin alleviated autophagic damage and mitochondrial dysfunction in AD (Fig. 6A and B, Fig. 6C–L, Fig. 6M and N J, and P). Furthermore, miR375-3p activated the mTOR pathway, including activation of mTORC1 and mTORC2. mTORC1 activation stimulated S6K, impeded ULK1 phosphorylation, and weakened autophagy. Whereas mTORC2 activation enhanced Akt phosphorylation, thereby promoting cell proliferation. miR375-3p treatment had a dual effect on cell growth, which was regulated based on different environments and stimuli (Fig. 7C and E, Supplementary figure 1A) [39]^.^ In the simulated environment of AD (with the addition of Aβ25-35 and in APP/PS1 TG mice), miR375-3p initially inhibited PS1 expression, weakens the cleavage of APP, but has weak effect on mTORC1 and p70S6K phosphorylation. Rapamycin mainly blocks the mTORC1 pathway, enhancing the effect of miR375-3p on Beclin1. Therefore, the co-treatment of rapamycin and miR375-3p has a slight advantage in behavioral data and molecular mechanisms.

The bioinformatic analysis also predicted CD68, which was significantly associated with microglial cells in this study. Furthermore, the impact of astrocytes was observed to be minimal (Fig. 7A and B, Fig. 7C and DB and C). This study primarily involves two phenotypes of microglial cell activation: M1 and M2. M1 (classical activation) is predominantly involved with the release of pro-inflammatory factors and the promotion of inflammatory responses, whereas M2 (alternative activation) is associated with neurorepair, phagocytosis, and tissue regeneration, and is considered beneficial in AD [40]. The literature has revealed that over-expression M2 phenotype or increased M1 phenotype inhibition in AD mice can worsen the disease [18,19,41], highlighting the importance of balancing M1/M2 phenotypes [42]. Here, both *in vitro* and *in vivo* analyses confirmed that in AD conditions, co-treatment of miR375-3p and rapamycin significantly enhanced Iba1 expression (Fig. 7A and B, Fig. 7C and D, Fig. 7E and F, Fig. 7G and HB, C, M, N, Q, and R), thereby initiating microglial cell activation. Moreover, the CD80+/CD68+ ratio remained largely unchanged (Fig. 7C), indicating the preservation of M1/M2 balance in activated microglial cells and significant enhancement of neuroprotection against AD neurons. Recently, much research has been conducted on microglia, among which microglial classification based on LDAM, DAM, and MGnD is the focus of our attention and the object of our follow-up research.

This study found that miRNA-375-3p alleviates AD symptoms by blocking the expression of PS1 protein, but miRNA-375-3p itself causes minor autophagy damage, which is unfavorable for alleviating AD symptoms. This study alleviates the unfavorable aspect of miRNA-375-3p by combining rapamycin, which blocks the autophagy damage caused by miRNA-375-3p and enhances the exosome pathway to enhance the activation of microglia by miRNA-375-3p from this perspective. Therefore, the addition of rapamycin not only alleviates the unfavorable aspects of miRNA-375-3p but also enhances its effects on microglia. The key role of PS1 in AD is also a key factor in highlighting the important role of miRNA-375-3p in AD and the limitation of miRNA-375-3p in AD, which is not universal. The role of miRNA-375-3p in different environments may be different.

## 5 Conclusions

In summary (Fig. 8X), miR375-3p plays a crucial role in neuronal protection and memory deficit restoration in early AD. Furthermore, rapamycin addition significantly enhances miR375-3p efficacy. This multi-target treatment strategy can effectively cross the blood-brain barrier and exhibits excellent biocompatibility, therefore, it can serve as a potential new target. this research provides directions for the diagnosis and development of early-stage AD therapies.

**Fig. 8X:**
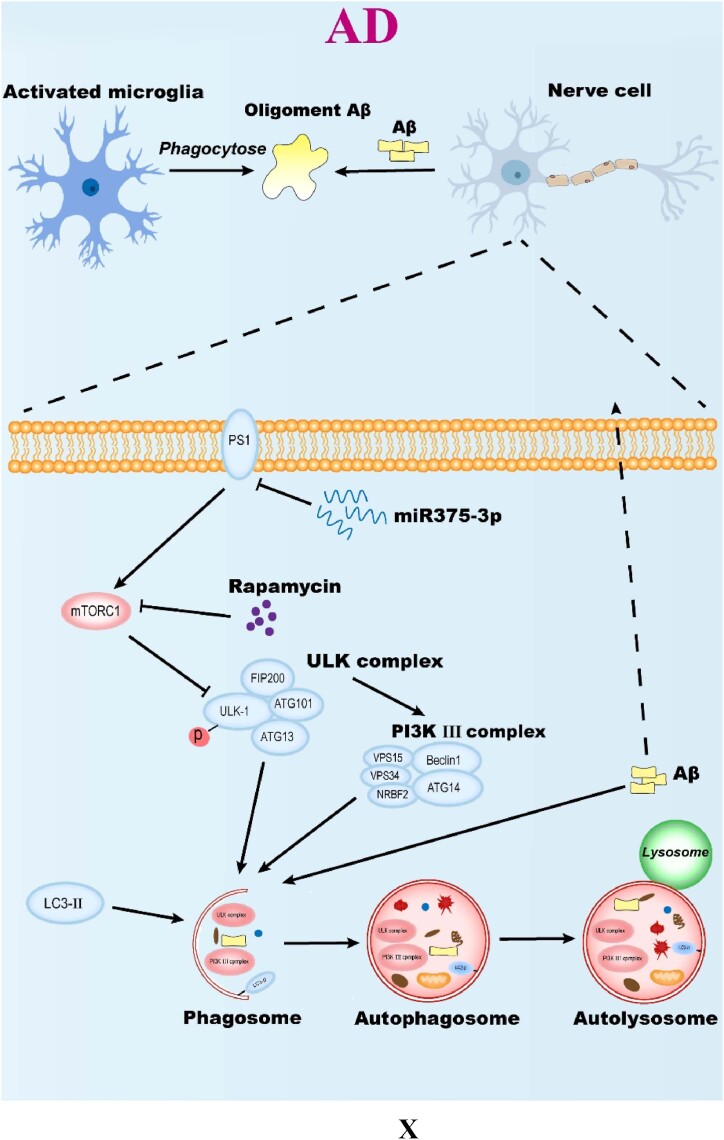
Molecular mechanism of overexpression of miR375-3p combined with rapamycin to clear Aβ deposition in AD environment. All data are presented as the mean ± SEM of at least three independent experiments and were compared using one-way ANOVA with Tukey's multiple comparisons test. *∗p < 0.05, ∗∗p < 0.01, ∗∗∗p < 0.001, ∗∗∗∗p < 0.0001*.

## Compliance with ethics requirements

All Institutional and National Guidelines for the care and use of animals (mice) were followed. Approval number：YNPZSY2023005. IACUC Issue No: (2023) YNPZSY (0201). Ethics committee: Welfare Ethics Committee, College of Life Sciences, Jilin University.

## Data availability statement

The authors confirm that the data supporting the findings of this study are available within the article.

## Funding

This work was supported by the 10.13039/501100012166National Key Research and Development Program of China, [grant number 2021YFA1500400], and Science and Technology Department of 10.13039/501100003807Jilin Province, [grant number YDZJ202301ZYTS536].

## CRediT authorship contribution statement

**Yuxiang Wang:** Writing – original draft, Data curation, Conceptualization. **Zixuan Xiao:** Data curation. **Hanlan Yin:** Data curation. **Zhichao Ren:** Formal analysis. **Xueting Ma:** Formal analysis, Data curation. **Yibo Wang:** Formal analysis. **Yan Zhang:** Formal analysis. **Xueqi Fu:** Funding acquisition. **Fuqiang Zhang:** Writing – original draft, Conceptualization. **Linlin Zeng:** Writing – review & editing, Funding acquisition, Conceptualization.

## Declaration of competing interest

The authors declare that they have no known competing financial interests or personal relationships that could have appeared to influence the work reported in this paper.
