# Supplementary material for: miRNA375-3p/rapamycin mediates the mTOR pathway by decreasing PS1, enhances microglial cell activity to regulate autophagy in Alzheimer's disease
Source: Heliyon. 2024 Sep 19;10(19):e37589. doi: 10.1016/j.heliyon.2024.e37589 (PMC11461998; doi:10.1016/j.heliyon.2024.e37589)
Supplement: Multimedia component 1 [file mmc1.doc]

**Supplementary Figure 1:**

**
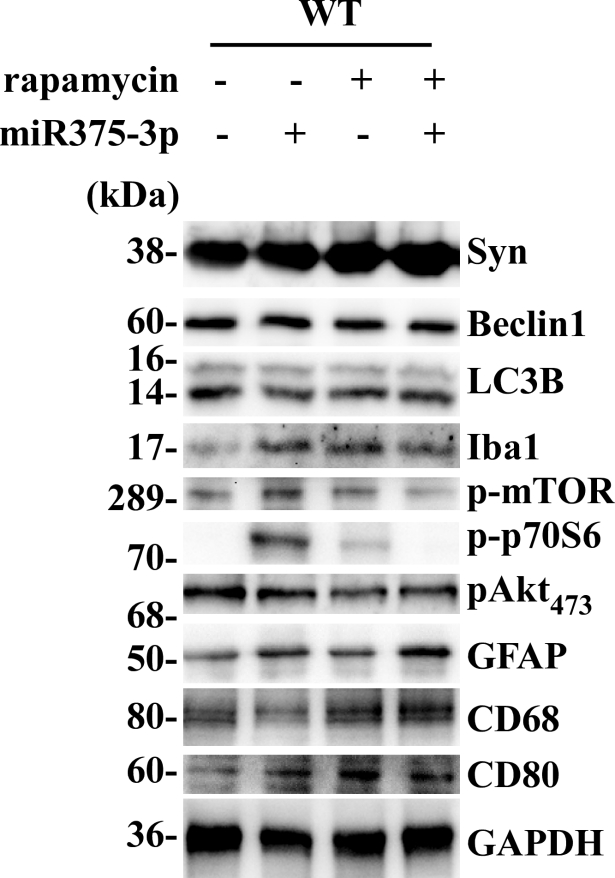
**

**Supplementary Figure 1A**

**
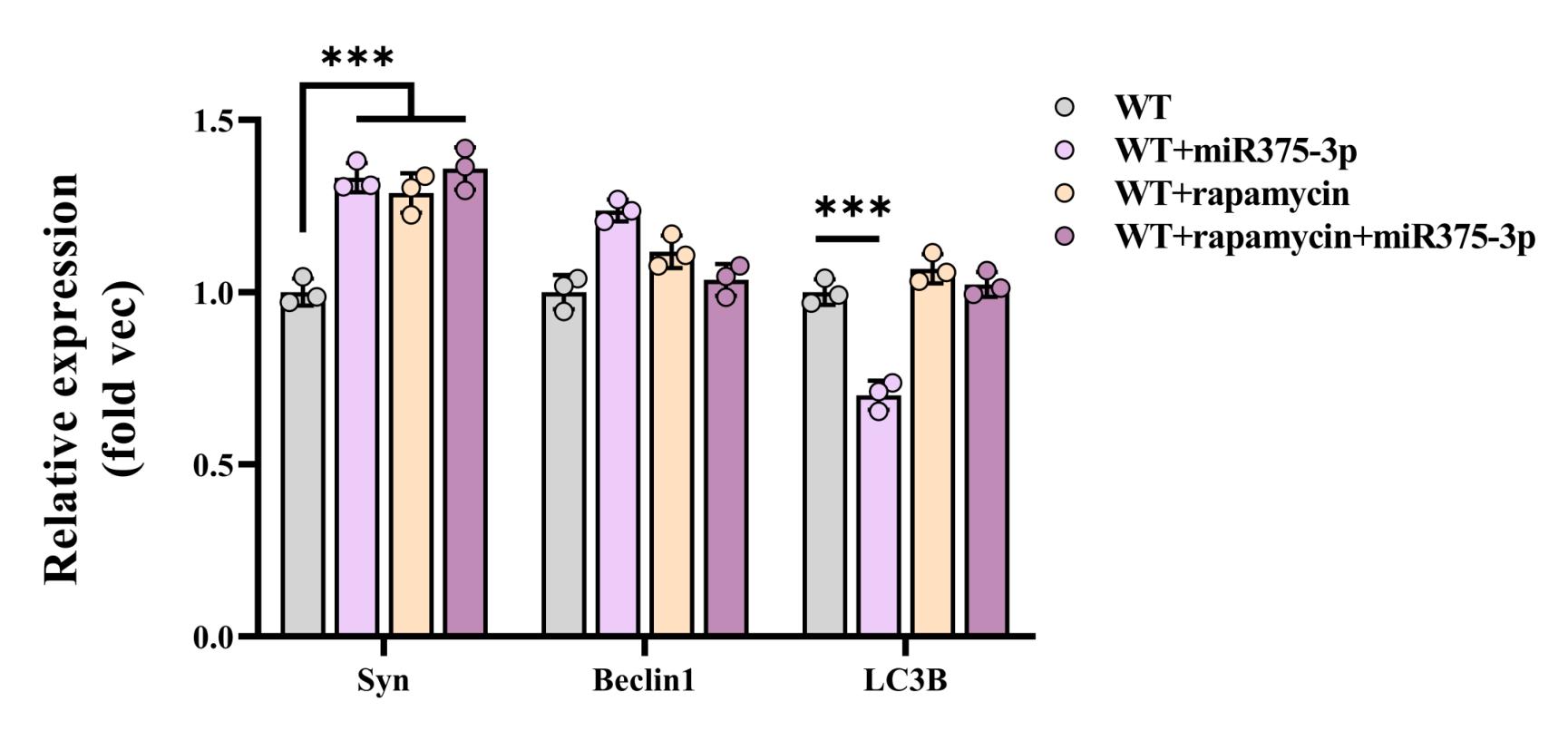
**

**Supplementary Figure 1B**

**
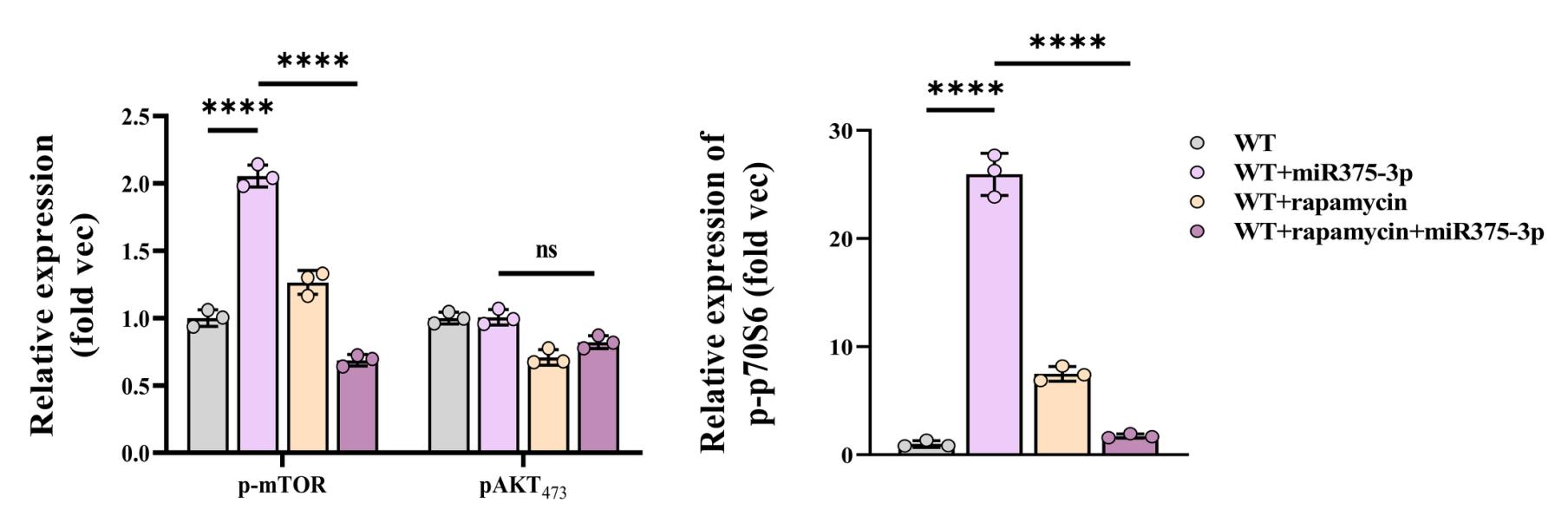
**

**Supplementary Figure 1C**

**
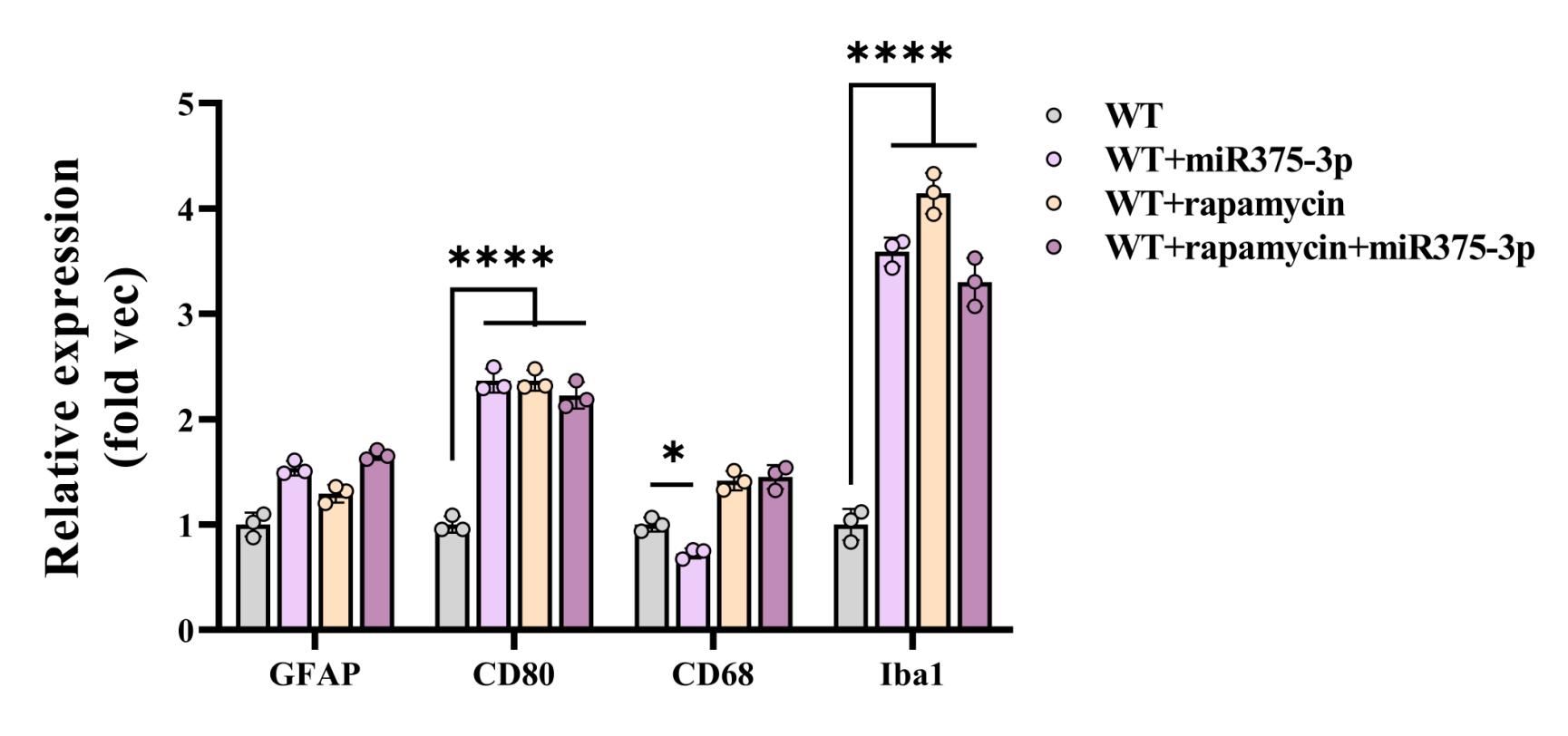
**

**Supplementary Figure 1D**

**Supplementary Figure 1A - D:** Western blot analysis was conducted to detect the protein expression in the parietal and temporal lobes of the brains of wild-type mice. (A) Protein expression levels of Syn, Beclin1, LC3B, p-mTOR, p-p70S6, p-AKT473, GFAP, Iba1, CD68, and CD80 are examined. Grayscale values of Syn, Beclin1, LC3B, (B) p-mTOR, p-p70S6, p-AKT_473_ (C), GFAP, Iba1, CD68, and CD80 (D) in each group are analyzed using imageJ, GAPDH was used as an internal reference.


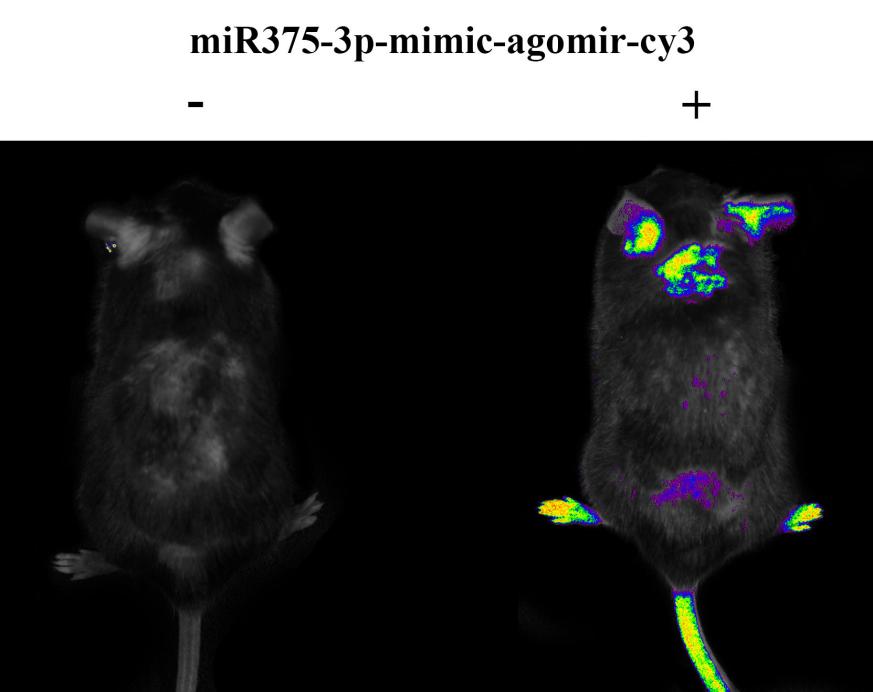


**Supplementary Figure 1E**

**Supplementary Figure 1E:** 0.8% saline and miR375-3p-mimic-agomir-cy3 were intravenously injected into wild-type mice, followed by anesthesia 24 hours later for live imaging observation using the 633 nm channel.


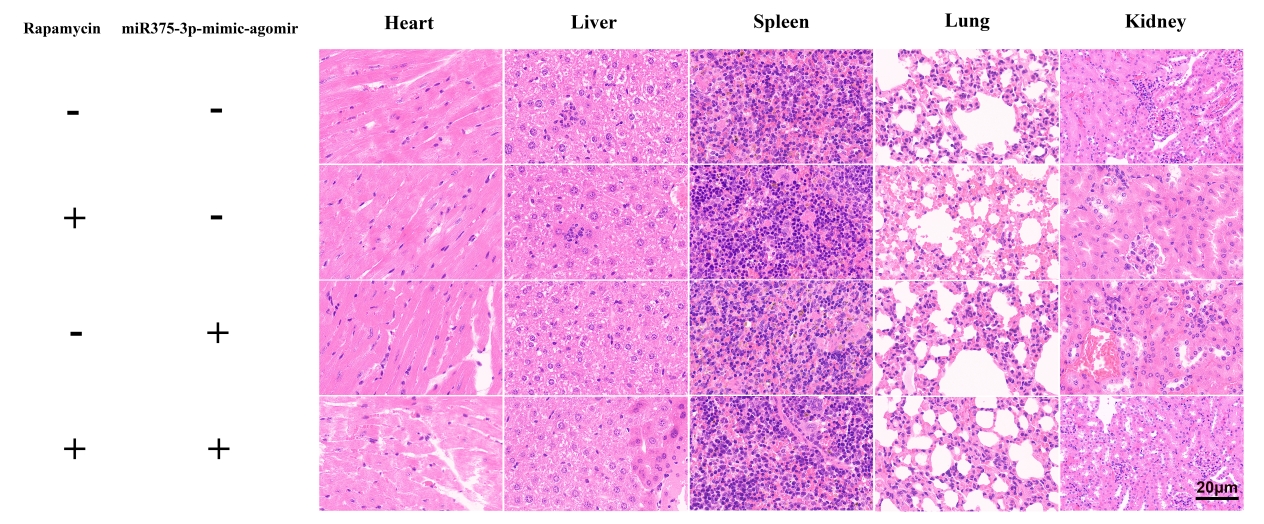


**Supplementary Figure 1F**

**Supplementary Figure 1F:** Rapamycin and miR375-3p-mimic-agomir were injected intraperitoneally and intravenously, respectively, into wild-type mice. After the injection period, organs were harvested, and tissue sections were prepared for HE staining analysis (400×): scale bar = 20 μm.


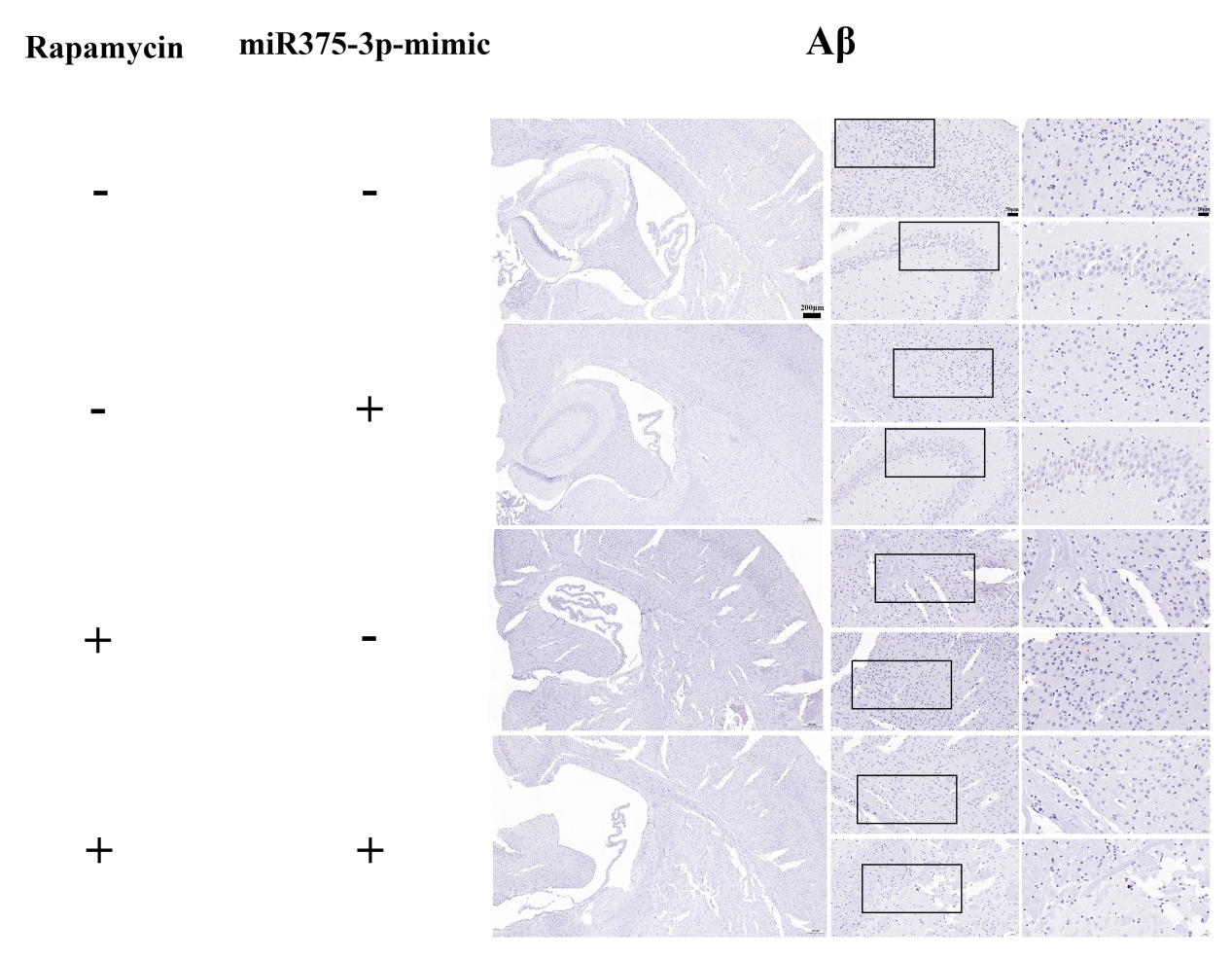


**Supplementary Figure 1G**

**
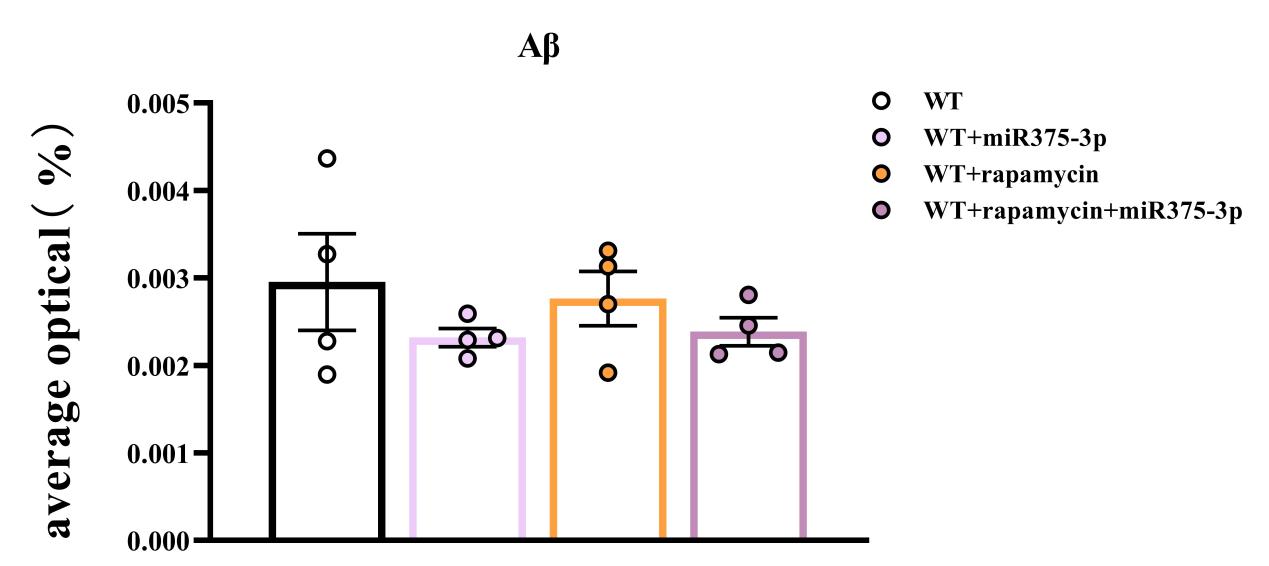
**

**Supplementary Figure 1H**


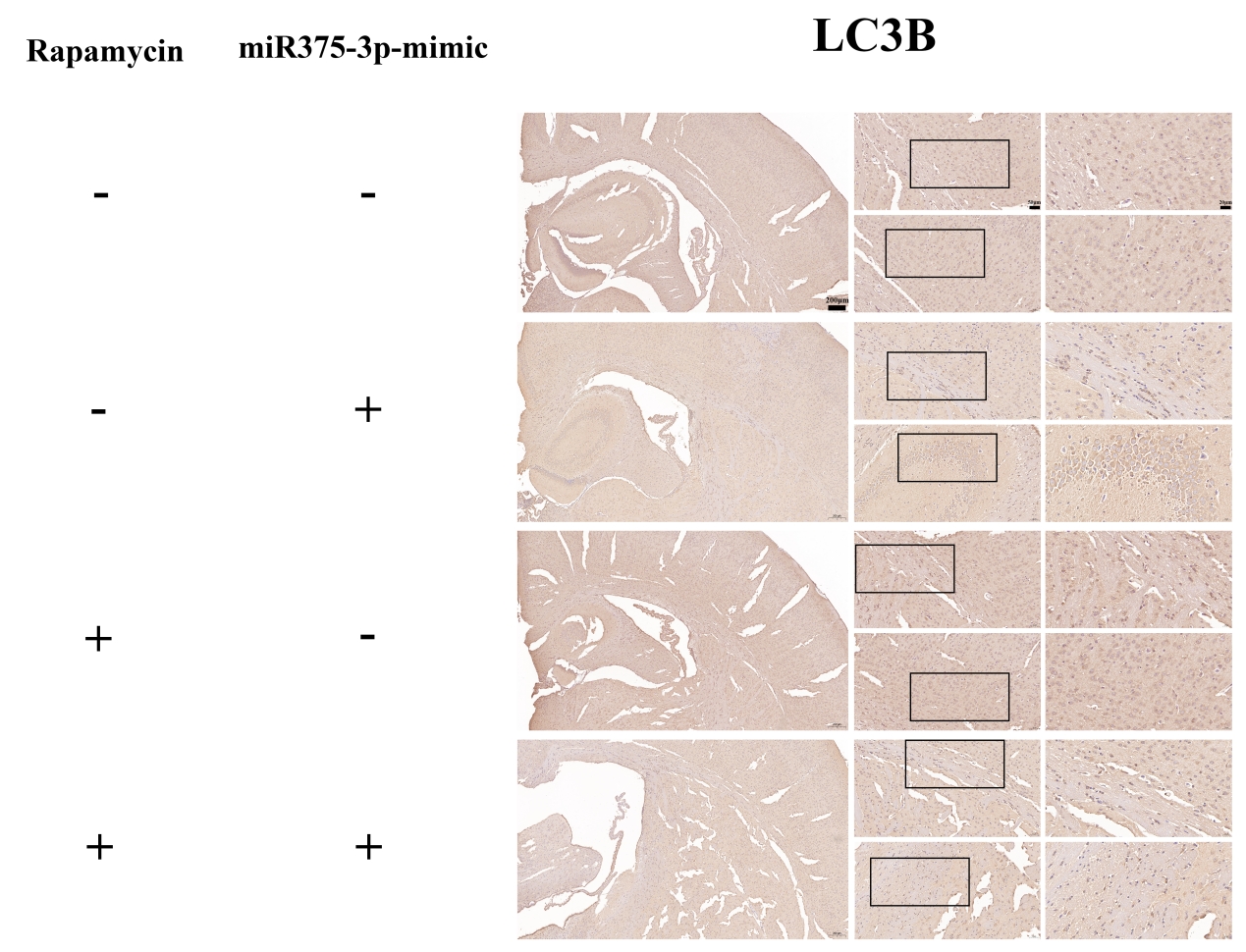


**Supplementary Figure 1I**

**
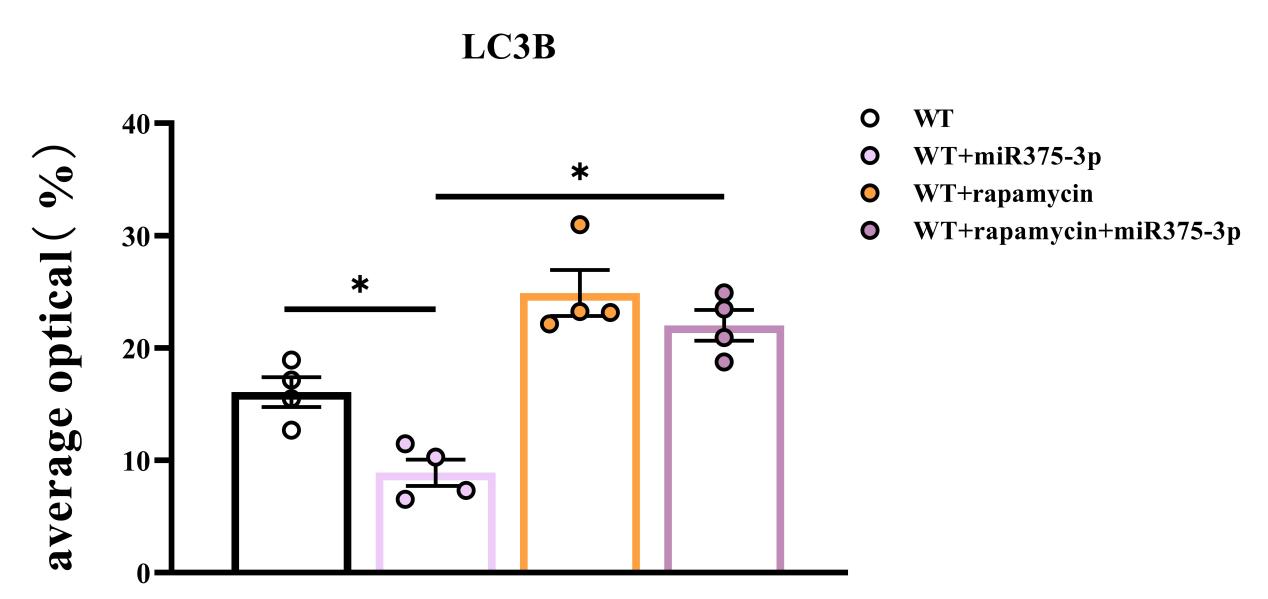
**

**Supplementary Figure 1J**


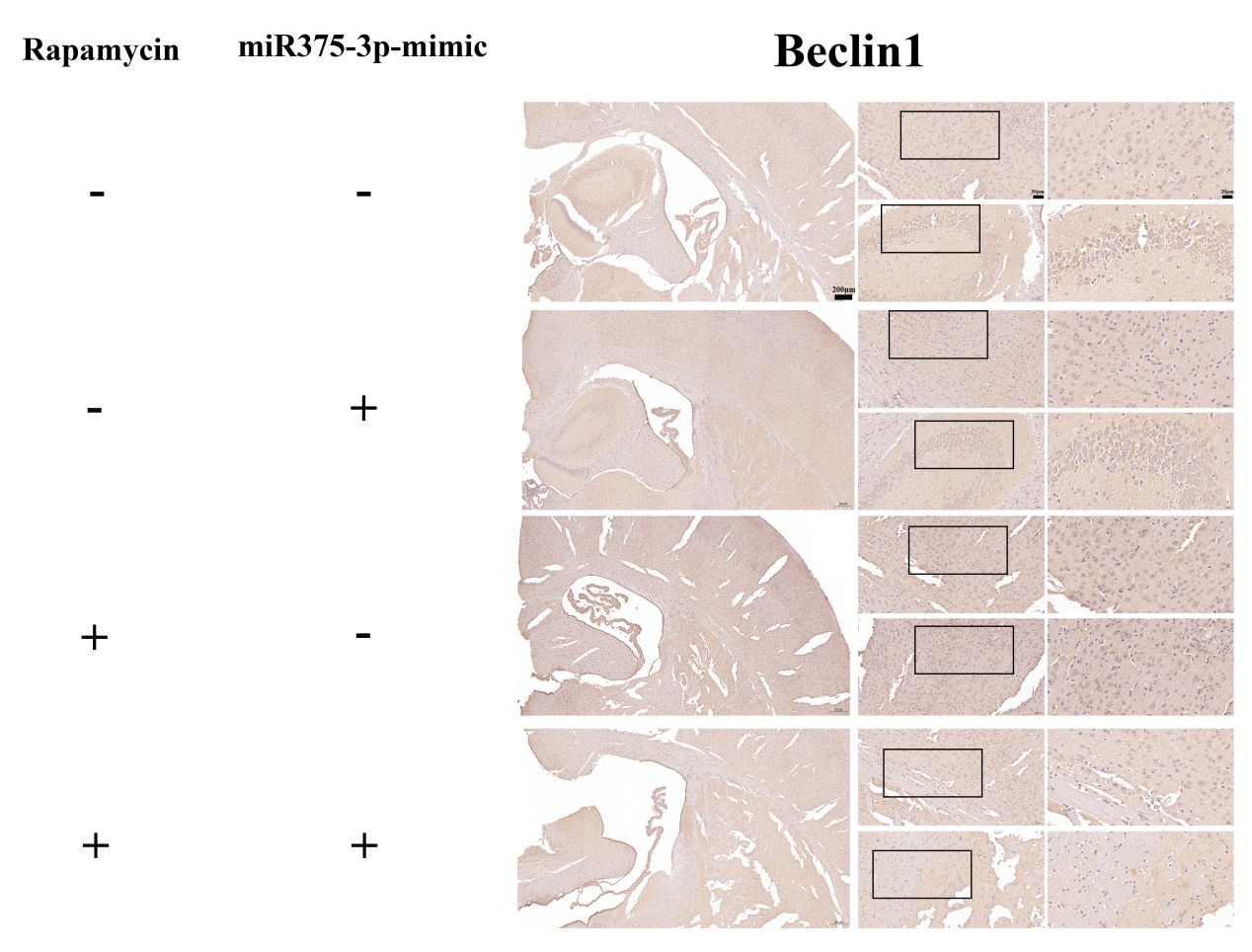


**Supplementary Figure 1K**

**
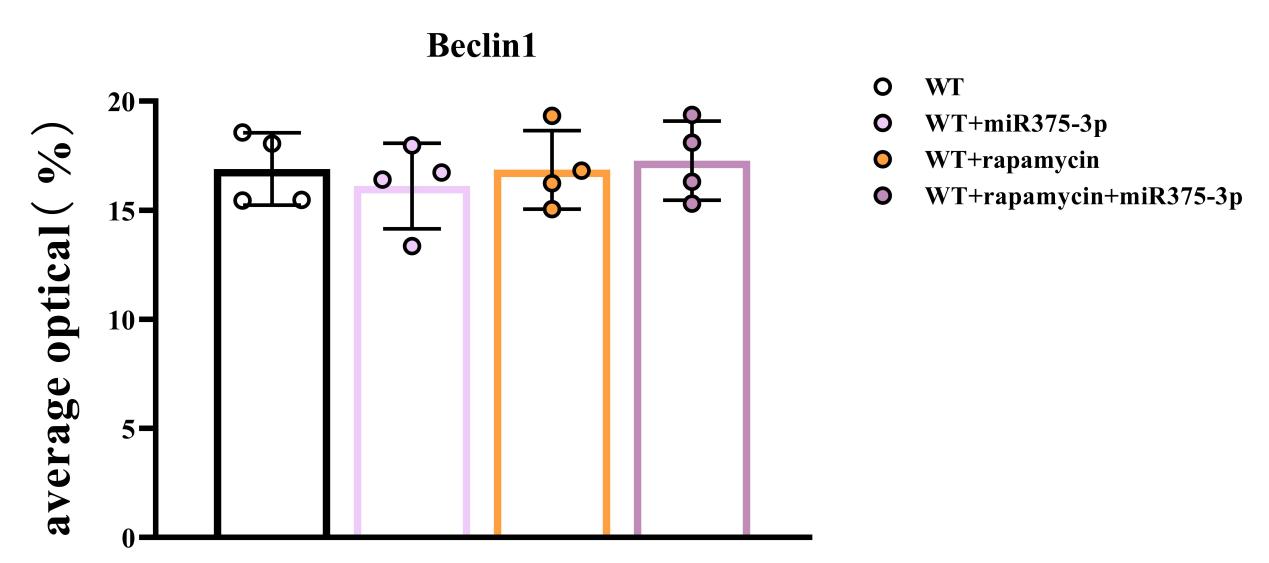
**

**Supplementary Figure 1L**


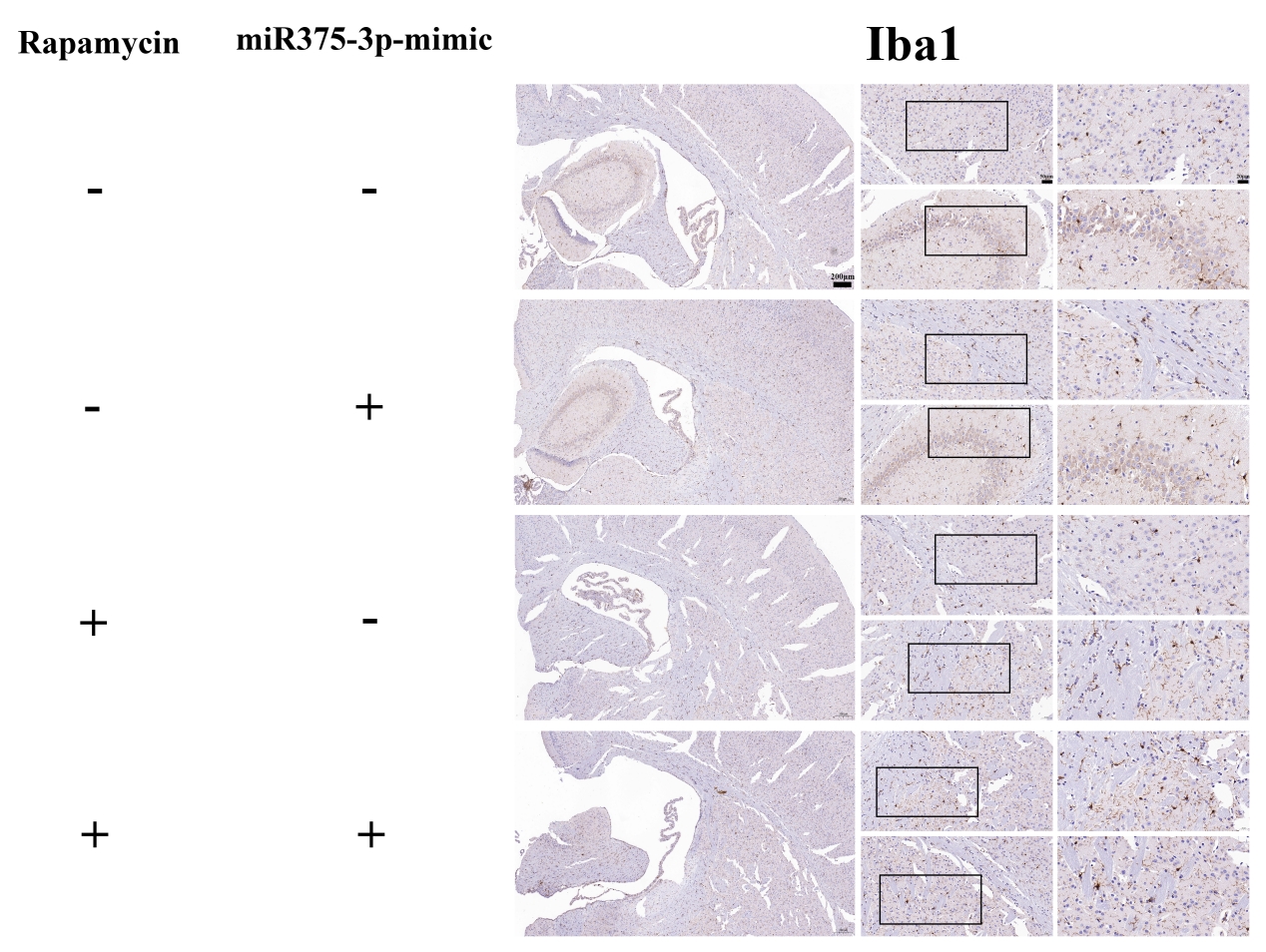


**Supplementary Figure 1M**

**
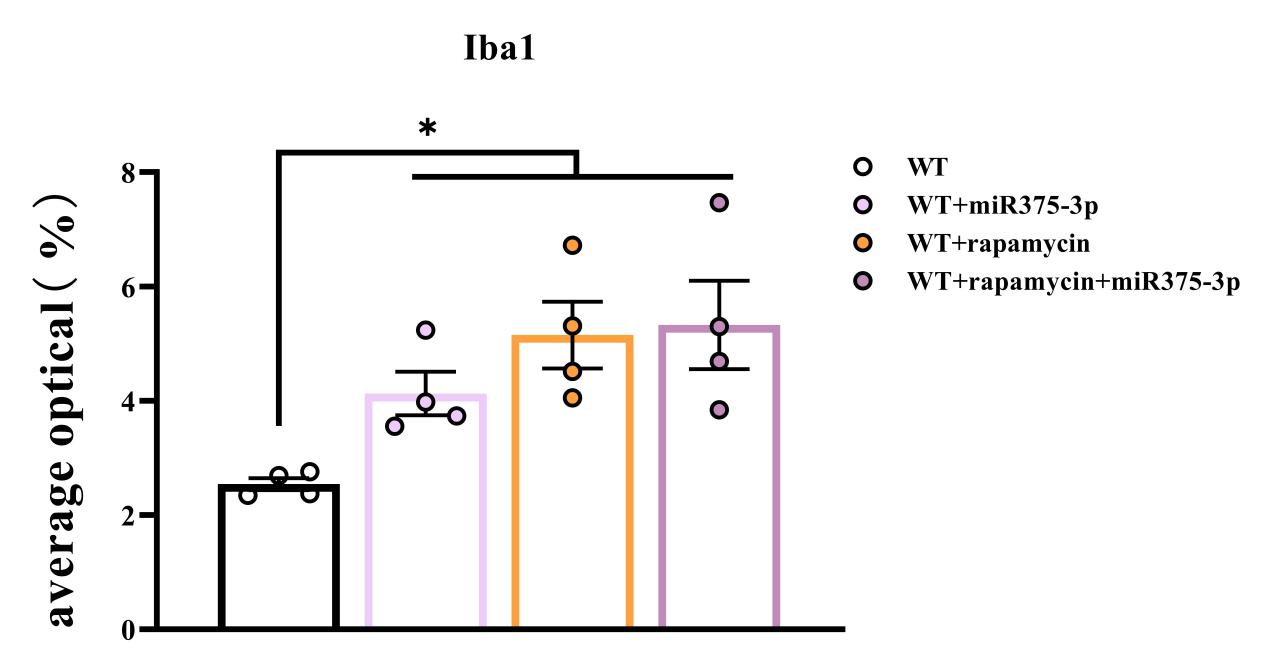
**

**Supplementary Figure 1N**


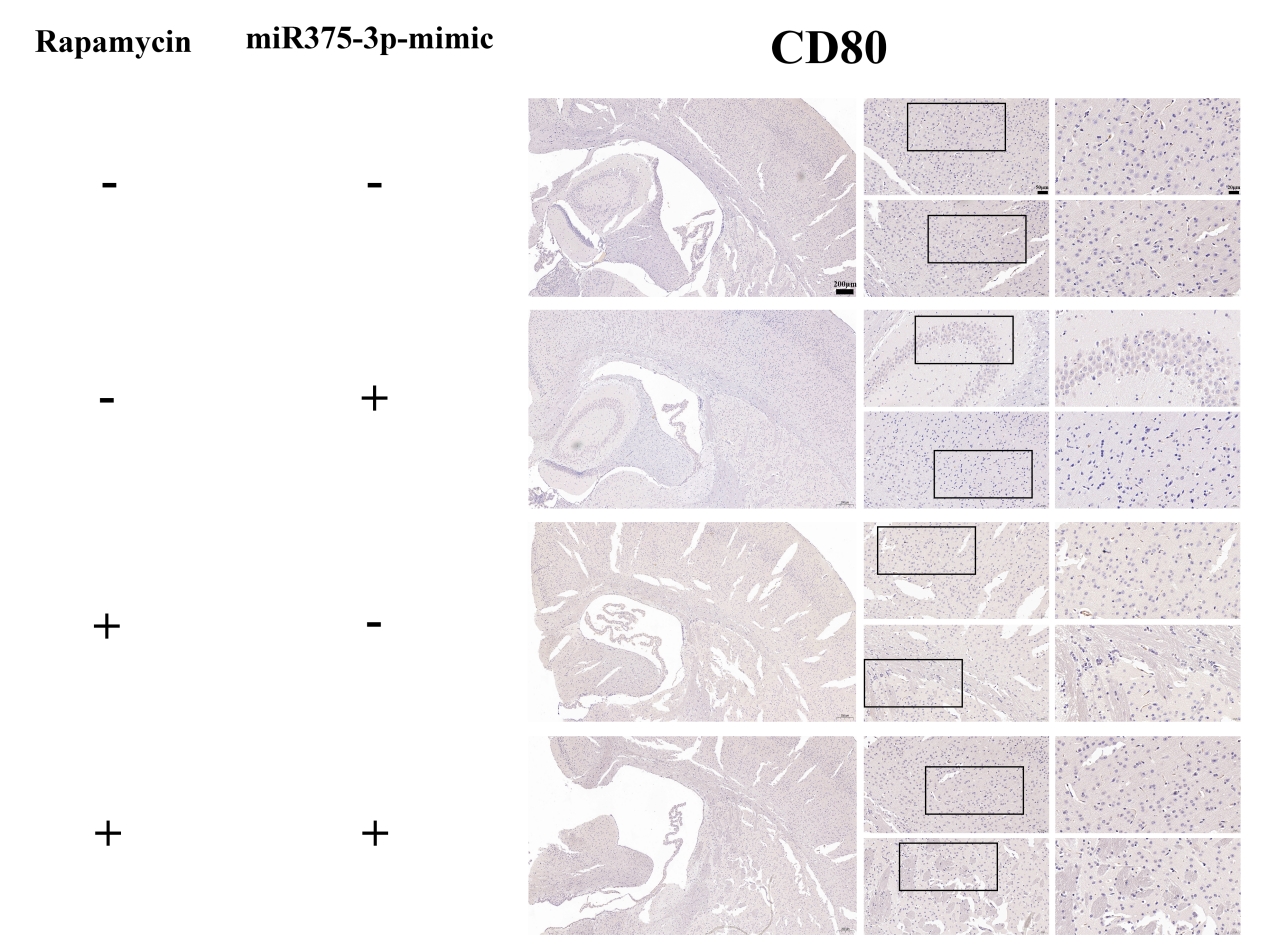


**Supplementary Figure 1O**


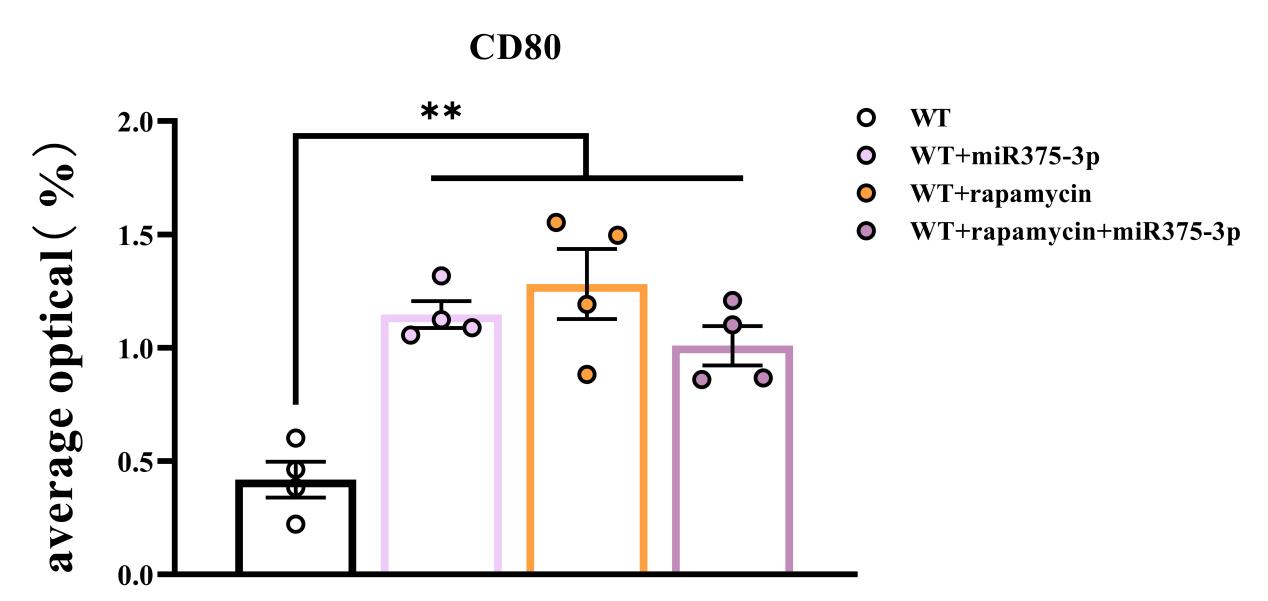


**Supplementary Figure 1P**


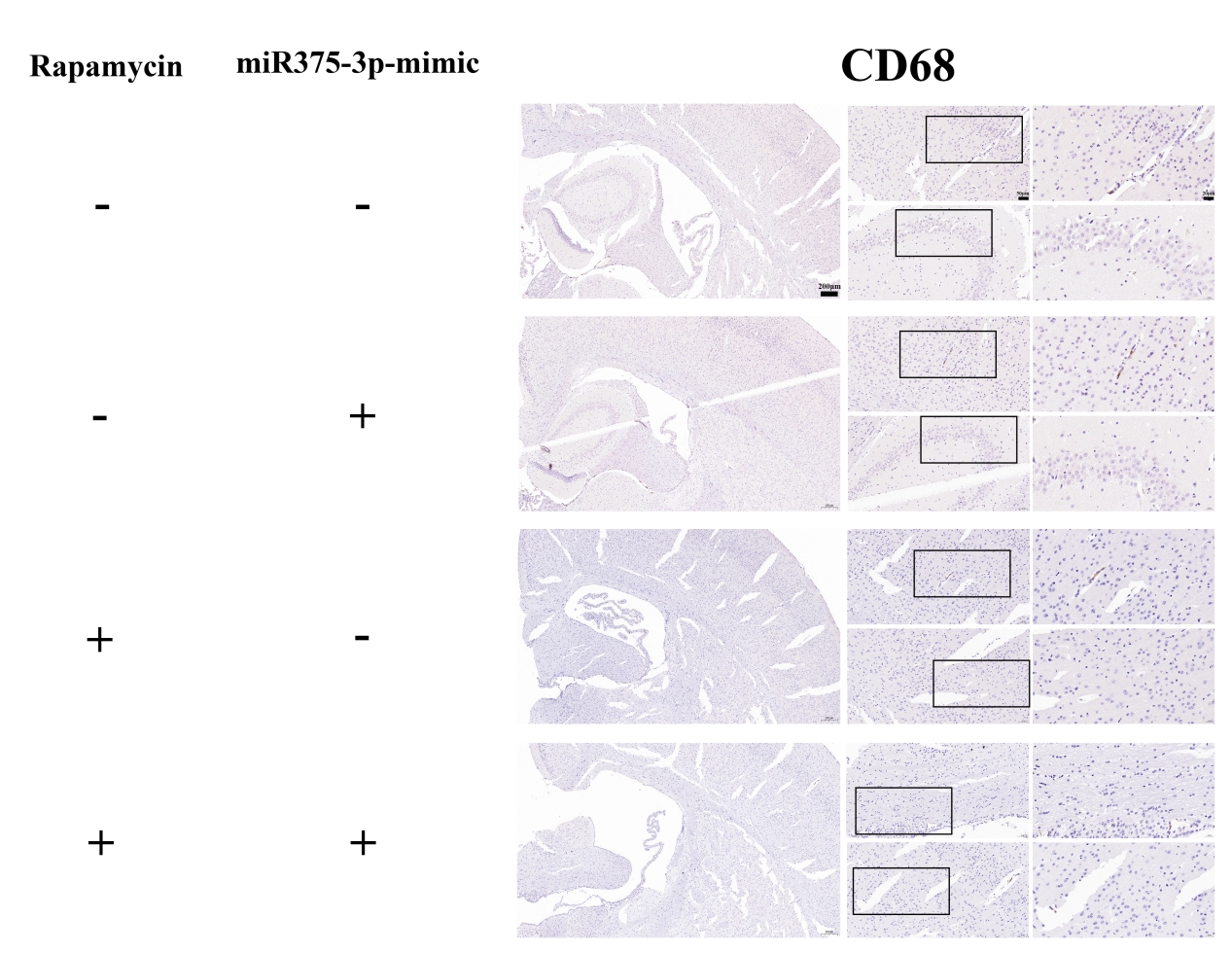


**Supplementary Figure 1Q**


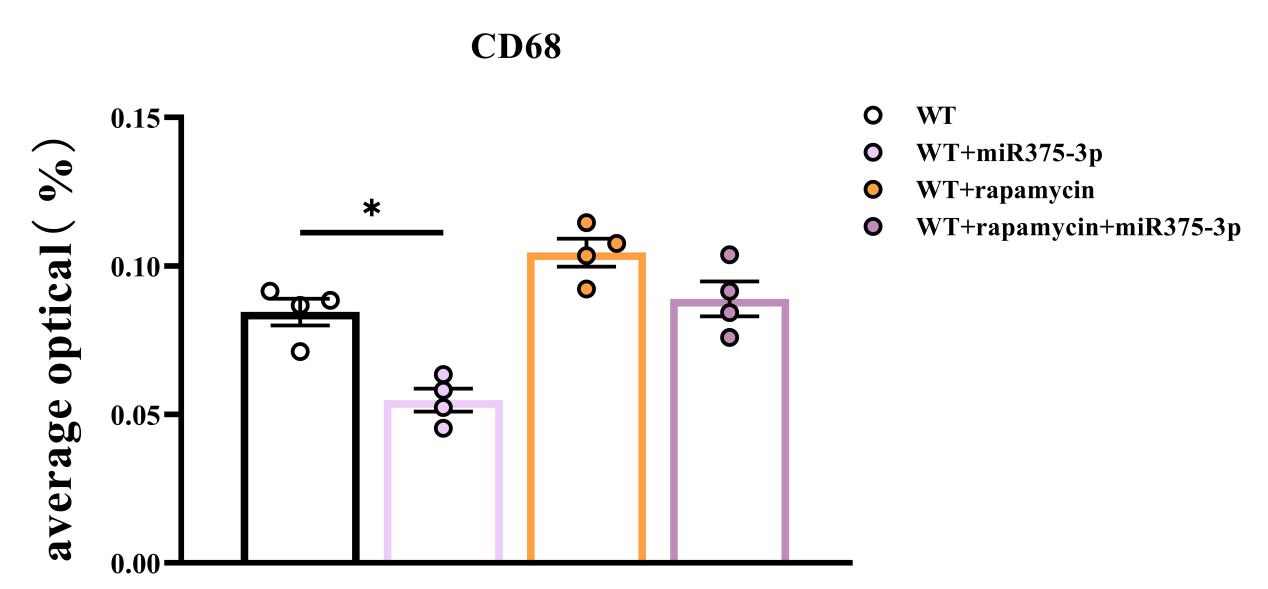


**Supplementary Figure 1R**

**Supplementary Figure 1G - R:** Immunohistochemical staining was performed to detect the protein expression of Aβ, LC3B, Beclin1, Iba1, CD80, and CD68 in the hippocampal region of WT mice. Whole brains from each group of mice were sectioned longitudinally, and immunohistochemical staining was performed using Anti-Aβ (G), Anti-LC3B (I), Anti-Beclin1 (K), Anti-Iba1 (M), Anti-CD80 (O), and Anti-CD68 (Q) antibodies. (H) The positive expression patches of Aβ (H), LC3B (J), Beclin1 (L), Iba1 (N), CD80 (P), and CD68 (R) in each group were quantified and analyzed using image-pro-plus 6.0.


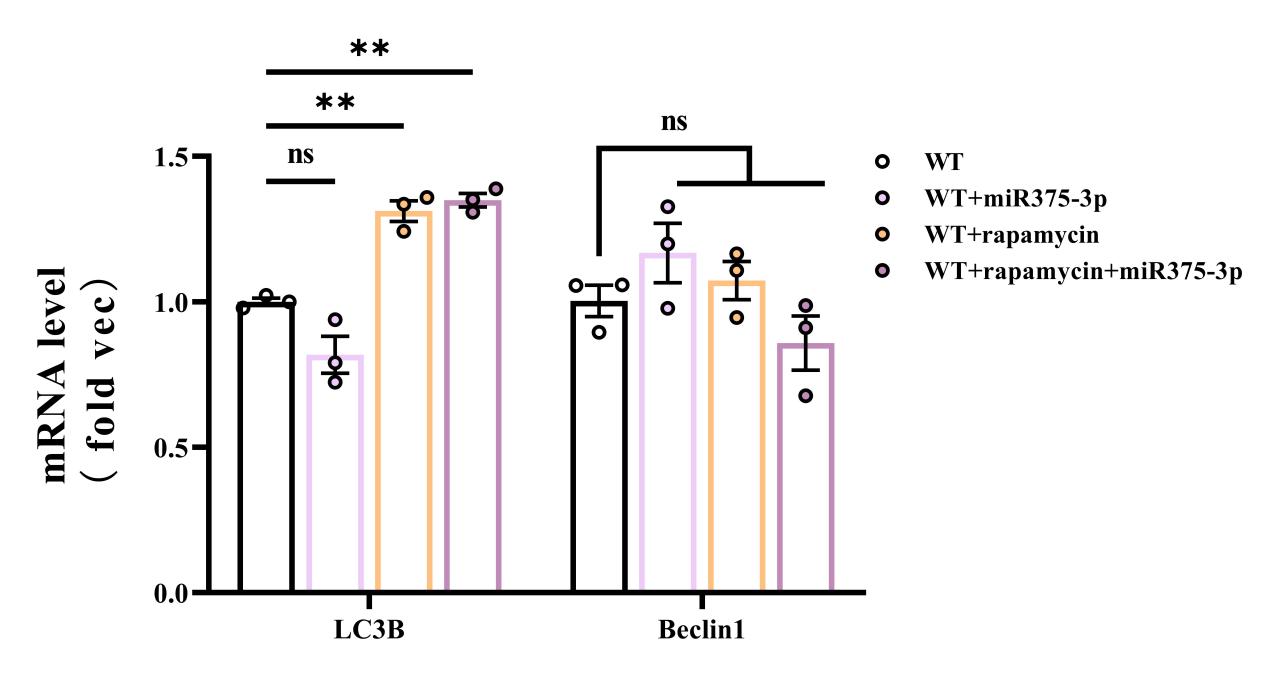


**Supplementary Figure 1S**

**
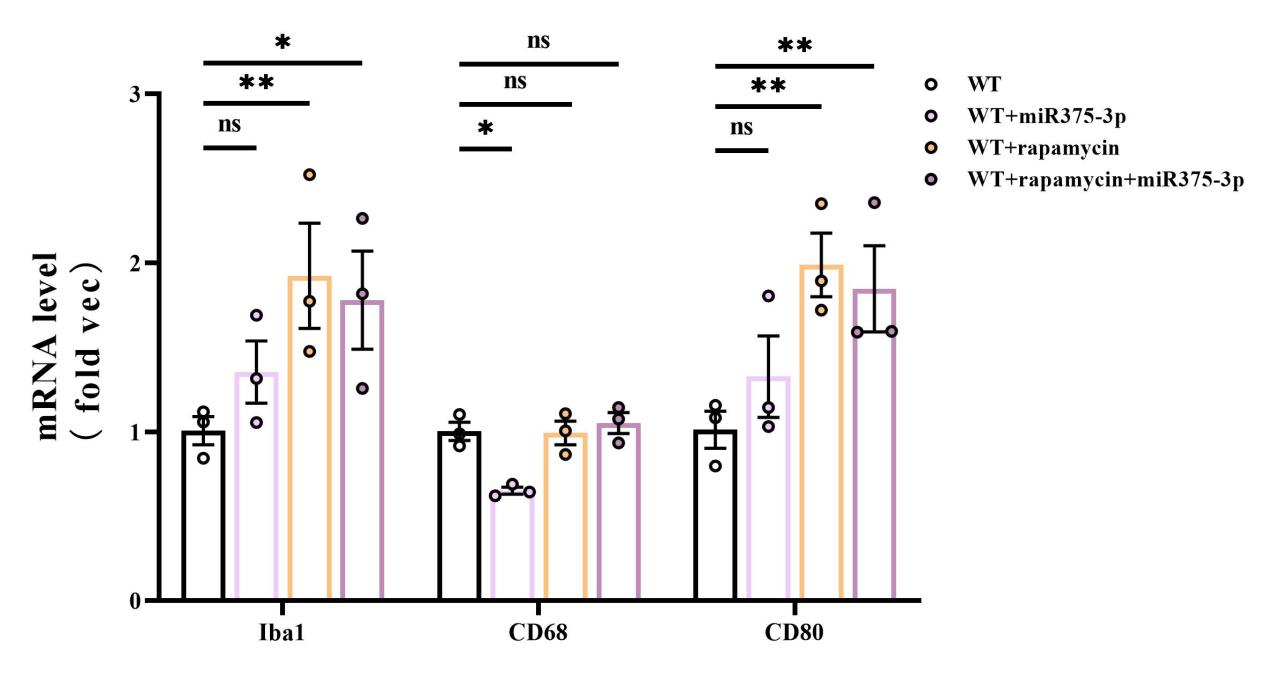
**

**Supplementary Figure 1T**

**Supplementary Figure 1S and T:** qRT-PCR was conducted to detect the mRNA expression levels in each group of wild-type mice. (S) Total RNA was extracted from the blood of each group of mice, reverse transcribed, and used as a template for qRT-PCR to detect the mRNA levels of autophagy markers: LC3B and Beclin1. (T) qRT-PCR was performed to detect the mRNA levels of CD68, CD80 and Iba1 in each group of mice.

**Supplementary Figure 2:**

**Supplementary Figure 2: miR375-3p overexpression promotes HMC-3 activation.**

**
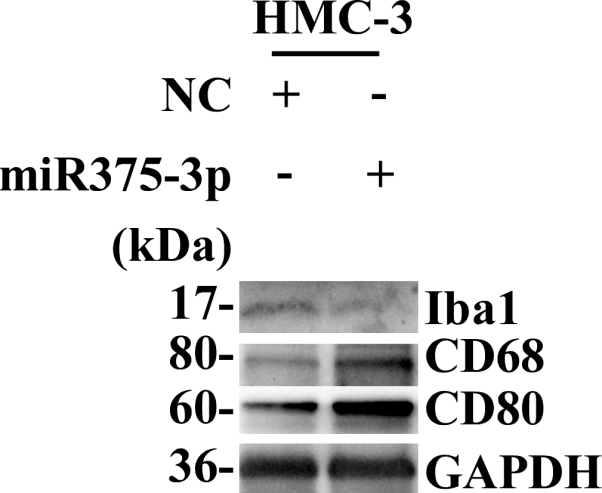
**

**Supplementary Figure 2A**

**
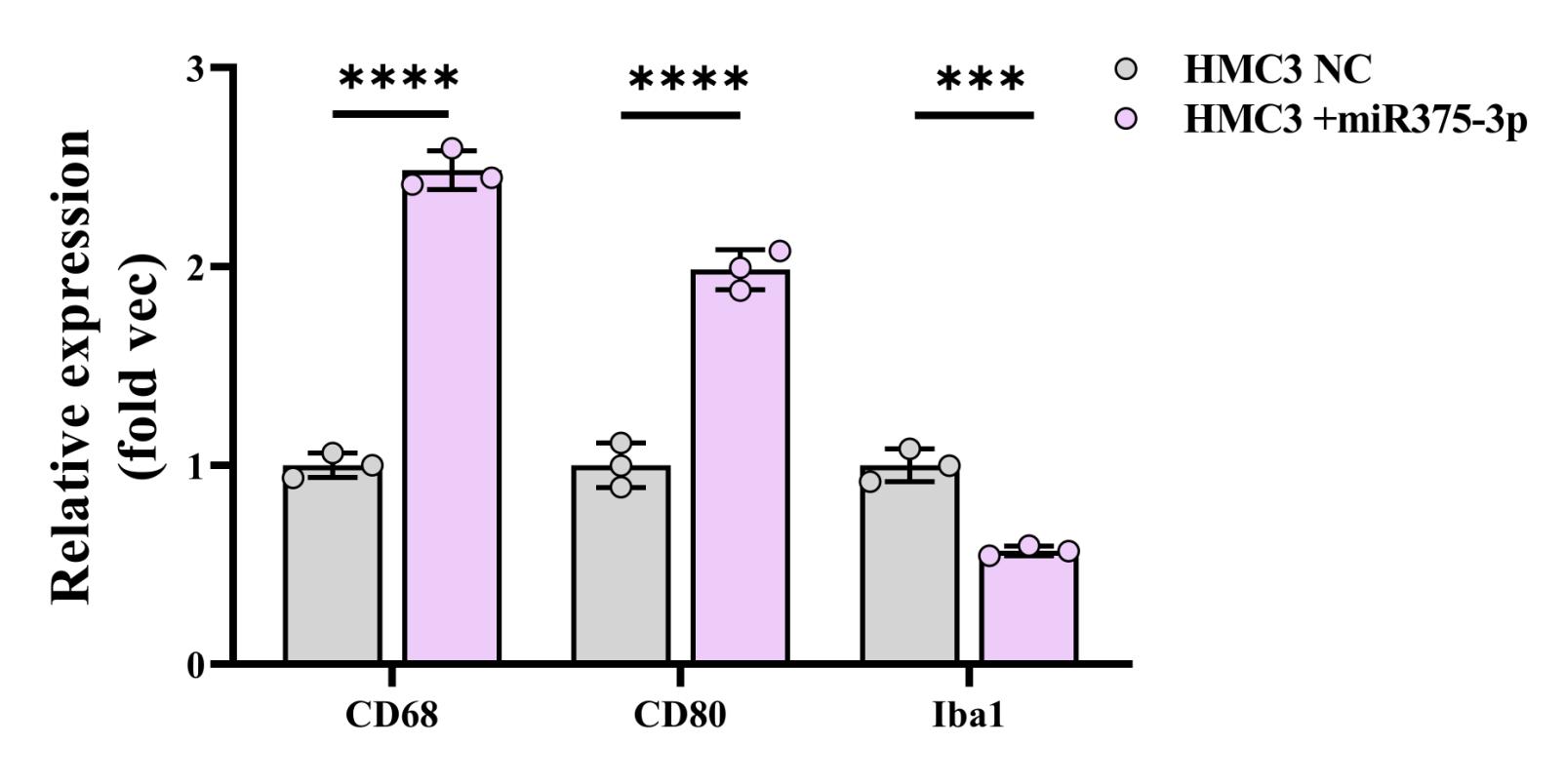
**

**Supplementary Figure 2B**

**Supplementary Figure 2A and B:** Western blot analysis was performed to detect the protein expression in various groups of HMC3 cells. (A) Protein expression levels of Iba1, CD68, and CD80 in each group of HMC3 cells are examined. (B) Grayscale values of each group are analyzed using imageJ, GAPDH was used as an internal reference.
